# Candidate genes for male and female reproductive traits in Canchim beef cattle

**DOI:** 10.1186/s40104-017-0199-8

**Published:** 2017-08-23

**Authors:** Marcos Eli Buzanskas, Daniela do Amaral Grossi, Ricardo Vieira Ventura, Flavio Schramm Schenkel, Tatiane Cristina Seleguim Chud, Nedenia Bonvino Stafuzza, Luciana Diniz Rola, Sarah Laguna Conceição Meirelles, Fabiana Barichello Mokry, Maurício de Alvarenga Mudadu, Roberto Hiroshi Higa, Marcos Vinícius Gualberto Barbosa da Silva, Maurício Mello de Alencar, Luciana Correia de Almeida Regitano, Danísio Prado Munari

**Affiliations:** 1 Departamento de Zootecnia, Universidade Federal da Paraíba (UFPB), Areia, Paraíba 58397-000 Brazil; 2 Fast Genetics, Saskatoon, SK S7K 2K6 Canada; 3 Beef Improvement Opportunities (BIO), Guelph, ON N1K 1E5 Canada; 4 Department of Animal and Poultry Science, University of Guelph, Centre for Genetic Improvement of Livestock (CGIL), Guelph, ON N1G 2W1 Canada; 5 Departamento de Ciências Exatas, Faculdade de Ciências Agrárias e Veterinárias, Universidade Estadual Paulista (Unesp), Jaboticabal, São Paulo 14884-900 Brazil; 6 Departamento de Zootecnia, Núcleo de Pesquisa e Conservação de Cervídeos, Faculdade de Ciências Agrárias e Veterinárias, Universidade Estadual Paulista (Unesp), Jaboticabal, São Paulo 14884-900 Brazil; 7 Department of Animal Science, Federal University of Lavras (UFLA), Lavras, Minas Gerais 37200-000 Brazil; 8 Department of Genetics and Evolution, Federal University of São Carlos (UFSCar), São Carlos, São Paulo 13565-905 Brazil; 9 Embrapa Agricultural Informatics, Campinas, São Paulo 13083-886 Brazil; 10 Embrapa Dairy Cattle, Juiz de Fora, Minas Gerais 36038-330 Brazil; 11 Embrapa Southeast Livestock, São Carlos, São Paulo 13560-970 Brazil

**Keywords:** Animal breeding, Composite breed, Genome-wide association, Genomic data, Single nucleotide polymorphism

## Abstract

**Background:**

Beef cattle breeding programs in Brazil have placed greater emphasis on the genomic study of reproductive traits of males and females due to their economic importance. In this study, genome-wide associations were assessed for scrotal circumference at 210 d of age, scrotal circumference at 420 d of age, age at first calving, and age at second calving, in Canchim beef cattle. Data quality control was conducted resulting in 672,778 SNPs and 392 animals.

**Results:**

Associated SNPs were observed for scrotal circumference at 420 d of age (435 SNPs), followed by scrotal circumference at 210 d of age (12 SNPs), age at first calving (six SNPs), and age at second calving (four SNPs). We investigated whether significant SNPs were within genic or surrounding regions. Biological processes of genes were associated with immune system, multicellular organismal process, response to stimulus, apoptotic process, cellular component organization or biogenesis, biological adhesion, and reproduction.

**Conclusions:**

Few associations were observed for scrotal circumference at 210 d of age, age at first calving, and age at second calving, reinforcing their polygenic inheritance and the complexity of understanding the genetic architecture of reproductive traits. Finding many associations for scrotal circumference at 420 d of age in various regions of the Canchim genome also reveals the difficulty of targeting specific candidate genes that could act on fertility; nonetheless, the high linkage disequilibrium between loci herein estimated could aid to overcome this issue. Therefore, all relevant information about genomic regions influencing reproductive traits may contribute to target candidate genes for further investigation of causal mutations and aid in future genomic studies in Canchim cattle to improve the breeding program.

**Electronic supplementary material:**

The online version of this article (doi:10.1186/s40104-017-0199-8) contains supplementary material, which is available to authorized users.

## Background

Cattle breeding programs in Brazil have given greater emphasis to the study and selection of reproductive traits due to their economic importance for the production system. In males, scrotal circumference traits are related to the reproductive potential of bulls, because testis size is associated with the production and quality of sperm and the production of sex hormones [1].

In general, female reproductive traits are difficult to measure and, in some cases, are strongly influenced by environmental factors. The reproductive performance of heifers depends on the age at which they calve for the first time; the ones that calve earlier have a more productive life [2]. In addition to first calving, another important factor is that the cow continues producing calves regularly to maintain its productivity and diminish calving interval [3]. Studies have reported that indirect selection of females based on the performance of bulls is possible, considering the favorable genetic correlations between scrotal circumference measures and age at calving [2, 4, 5].

Many achievements in animal breeding were obtained based on the classical approach, using information from phenotypes and pedigree. Nowadays, molecular data analyses are bringing new insights to the genetic architecture of species. Regarding reproductive traits of beef cattle, genome-wide association studies (GWAS) are useful tools for the identification of candidate genes that could be used to classify precocious or more fertile individuals [6, 7].

The identification of candidate genes provides a better understanding of the distribution of genes that affect traits of economic interest, as well as a basis for further studies to identify causal mutations. Despite its potential, important observations are needed for this approach. According to Tabor et al. [8], the difficulty of replication over time or across populations in this approach might indicate that more detailed studies are needed to certify its causality. Therefore, the aim of this study was to perform a GWAS to identify genomic regions and candidate genes to uncover the genetic architecture of scrotal circumference at 210 and 420 d of age and age at first and second calving and aid in the breeding process in Canchim cattle.

## Methods

### Canchim breed

The Canchim is a composite breed (62.5% Charolais and 37.5% Zebu) developed in the 1940s by the Brazilian Agricultural Research Corporation (Embrapa), located in São Carlos city, SP, Brazil [9]. Different crossbreeding schemes have been studied to produce Canchim cattle with different Charolais-Zebu proportions and to achieve greater genetic variability in the population [10–13]. One of these schemes was used to produce animals of the “MA” genetic group, which was derived from mating Canchim-Zebu animals (F1) with Charolais animals, resulting in approximate contributions of 65.6% Charolais and 34.4% Zebu [14].

The Canchim cattle represents about 3% of the beef cattle produced in Brazil [15]. Indicine breeds are much more representative as purebreds or crossbred animals, being responsible for 80% of the beef cattle industry in the country [16].

### Traits analyzed

The estimated breeding values (EBVs) used in our study considered the following traits: scrotal circumference at weaning adjusted for 210 d of age (SC210), scrotal circumference adjusted for 420 d of age (SC420), age at first calving (AFC), and age at second calving (ASC). Linear interpolation was previously used to adjust the scrotal circumferences for 210 and 420 d of age.

The EBVs were obtained from the genetic evaluation carried out by the Brazilian Agricultural Research Corporation (Embrapa) for the Canchim breed which considered 318,307 animals in the relationship matrix and 267,002 animals with valid records. In general, the traits analyzed by Embrapa for the genetic evaluation of Canchim that could be highlighted are body weight traits measured at various ages, reproductive traits of males and females, carcass quality, navel visual score of males and females, and hair coat. The EBVs were estimated by multi-trait analysis using the REMLF90 program [17], under an animal model. For the studied traits, the fixed effects considered in the contemporary groups were year and season of birth (January to March; April to September; and October to December), farm of birth, genetic group, and feeding system.

As described by Mokry et al. [18], the genotyped animals were chosen according to the EBV for some traits (ribeye area, back fat thickness, and productive and reproductive traits), accuracy, family size, and proportion of males and females. The mean EBV values for SC210, SC420, AFC, and ASC were 1.44 mm, 2.07 mm, −4.34 d, and −1.23 d, respectively. Minimum and maximum values for SC210, SC420, AFC, and ASC varied from −8.76 to 12.74, −17.41 to 21.80, −50.89 to 44.64, and −42.46 to 44.66, respectively. De-regressed proofs were not used due to limited data. Furthermore, de-regressed proofs calculated with low accuracies are expected to have a smaller genomic contribution due to Mendelian sampling and will have more noise added in de-regressed calculations [19].

### Genotypes and quality control

The BovineHD BeadChip SNP panel (Illumina Inc., San Diego, CA) was used to genotype 194 males and 205 females: 285 animals of the Canchim breed and 114 animals of the MA genetic group, calves of 49 bulls and 355 cows. The animals were born between 1999 and 2005 and come from seven farms in the States of São Paulo and Goiás. A detailed description of these animals was previously presented by Buzanskas et al. [20] and Mokry et al. [18]. Genotype quality control excluded SNPs with significant deviations (*P* < 10^–5^) from the Hardy-Weinberg equilibrium, SNPs with minor allele frequencies of less than 0.05, and call rate for SNPs and animals lower than 0.90. Only autosomal chromosomes and SNPs with known positions, according to the UMD_3.1 bovine genome assembly [21], were used for GWAS.

### Genome-wide association study

The Generalized Quasi-Likelihood Score (GQLS) method [22] was used for GWAS. In this method, a logistic regression was used to associate the EBVs (treated as a covariate) to the genotypes (treated as the response variable), one SNP at a time. The EBVs were represented by *X*_*i*_ = (*X*_1_,  … , *X*_*n*_)^'^, in which Xi represents the EBVs for the ith animal. The genotype of the animals was coded as “0”, “1” or “2” and, as the genotypes were represented by *Y*_*i*_ = (*Y*_1_,  … , *Y*_*n*_)^'^, in which *Y*_*i*_ = ½ * (number of alleles for animal genotype i), the respective proportions would be equal to 0, ½ and 1. The expected SNP allele frequency is represented by *μ*, in which *μ* = (*μ*_1_,  … , *μ*_*n*_)^'^ = *E*(*X*| *Y*), thus 0 < *μ*_*i*_ < 1.

To associate *μ*_*i*_ with *X*_*i*_, the following logistic regression was defined:

$$ {\mu}_i=E\left({Y}_i|{\mathrm{X}}_{\mathrm{i}}\right)=\frac{e^{\beta_0+{\beta}_1{X}_i}}{1+{e}^{\beta_0+{\beta}_1{X}_i}} $$

in which *β*_0_ is the constant term and *β*_1_ is the slope.

Under the null hypothesis, *μ*_*i*_ can be interpreted by ${\mu}_i=\frac{e^{\beta_0}}{1+{e}^{\beta_0}}$, for all *i* = 1 ,  …  , *n*. The mean vector of *Y*_*i*_ no longer depends on *X*_*i*_, which becomes *μ*_*i*_ = *E*(*Y*) = *μ*1, and “1” is a vector. The solution of the “quasi-likelihood score” results in an estimate for μ, such that ${\widehat{\mu}}_i={\left({1}^{\hbox{'}}{A}^{-1}1\right)}^{-1}{1}^{\hbox{'}}{A}^{-1}Y$, in which A ^− 1^ is the inverse of the relationship matrix of the individuals.

To obtain the GQLS, the statistic WG was calculated as in Feng et al. [22], as follows:

$$ {W}_G=\frac{2}{\widehat{\mu}\left(1-\widehat{\mu}\right)}{\left[{X}^{\hbox{'}}{A}^{-1}\left(Y-\widehat{\mu}1\right)\right]}^{\hbox{'}}\times {\left[{X}^{\hbox{'}}{A}^{-1}X-\left({X}^{\hbox{'}}{A}^{-1}1\right){\left({1}^{\hbox{'}}{A}^{-1}1\right)}^{-1}\left({1}^{\hbox{'}}{A}^{-1}X\right)\right]}^{-1}\times \left[{X}^{\hbox{'}}{A}^{-1}\left(Y-\widehat{\mu}1\right)\right] $$

Under the null hypothesis, WG follows a Chi-squared distribution with one degree of freedom [23], resulting in *P*-values for each SNP. This method does not account for the genetic variance explained by the marker. The GQLS provides the advantage of accounting for the population structure by means of the pedigree-based relationship among animals (A ^− 1^).

The false discovery rate (FDR) was used for multiple testing corrections [24] to verify significant SNPs. The *P*-values of each SNP were sorted in ascending order and the following formula applied:

$$ q< mP(i)/i $$

in which *q* is the desired level of significance, *m* is the total number of SNPs, and *P* is the *P*-value of the ith SNP. To account for multiple comparisons, a genome-wide and chromosome-wise FDR of 5% (significant association) and 10% (suggestive association) was applied. The main reason for considering a minimum FDR of 10% was to obtain a comprehensive number of SNPs, which might assist to comprehend the genetic architecture of the studied traits.

Pairwise linkage disequilibrium was estimated using the r^2^ measure [25] for the SNPs with significant and suggestive associations. The software Haploview [26] was used to estimate r^2^ and plot the results. Due to the high linkage phase consistency between Canchim and MA genetic group (above 0.80 up to 100 kb) [27], analyses were conducted considering Canchim and MA as one population.

### Gene mapping and in silico functional analyses

The SNPs associated with the EBVs for SC210, SC420, AFC, and ASC traits were surveyed to their corresponding genes or to surrounding genes. The National Center for Biotechnology Information (NCBI) database [28] and Ensembl Genome Browser [29] were accessed to proceed with in silico functional analyses of the genes. If the SNP was located in the intergenic region (i.e. not assigned to any gene), we observed, through the integrated maps of the NCBI variation viewer, for the closest gene(s) and calculated the distance(s).

The PANTHER tool [30] was used to access and gather biological processes and pathway associations. The AnimalQTLdb database [31] was used to verify previous reports of quantitative trait loci (QTL) in the surroundings of significant SNPs. The main reason to use these tools was to validate our findings.

## Results

**Fig. 1:**
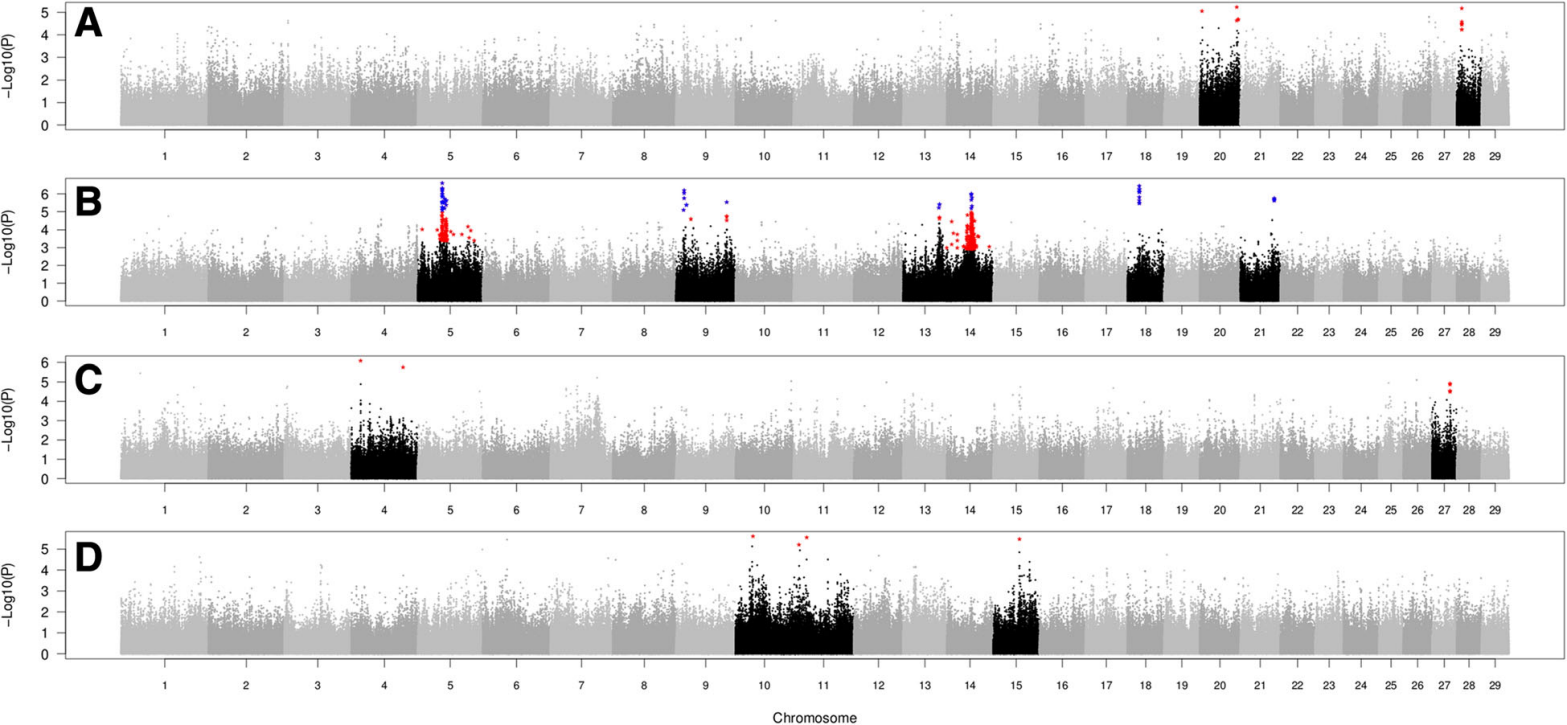
Manhattan plots for scrotal circumference at 210 d of age (**a**), scrotal circumference at 420 d of age (**b**), age at first calving (**c**), and age at second calving (**d**). Chromosomes with significant SNPs are highlighted in black. The significant SNPs, after false discovery rate correction of 10% (chromosome-wise), are highlighted in red. The significant SNPs, after false discovery rate correction of 10% (genome-wide), are highlighted in blue. On the y-axis are presented the –Log of the *P*-values for each SNP. On the x-axis are presented the autosomal chromosomes

A total of 672,778 SNPs and 392 animals were used for GWAS. The Manhattan plots for chromosome-wise (in blue) and genome-wide (in red) significantly associated SNPs after FDR correction for SC210, SC420, AFC, and ASC, respectively, are presented in Fig. 1. The greatest number of significant SNPs was observed for SC420 (435 SNPs), followed by SC210 (12 SNPs), AFC (six SNPs), and ASC (four SNPs) when considering FDR 10% for all traits.

In Table 1 are presented SNPs and genes identified for SC210, AFC, and ASC. For SC420, SNPs and genes are presented in Table 2. Due to the large number of SNPs associated with SC420, full information regarding genes, pseudogenes, and non-coding RNA are presented in Additional files 1, 2, and 3. For FDR 5%, a total of 249 (chromosome-wise) and 50 (genome-wide) SNPs were observed for SC420 (Additional file 1). Considering FDR 10%, the regions observed in Table 1 presented a suggestive association; therefore, the genes identified in these regions were surveyed.

**Table 1:** Chromosome-wise association for scrotal circumference (SC210), age at first (AFC) and second calving (ASC)

Trait	Genes	SNP Reference	Chr:Pos	Distance to gene	*P*-value (Min-Max)
SC210	*SMIM23*	rs135355728^a^	20:3.48	14.22	8.96E-06
SC210	*PAPD7*	rs137042056^a^, rs136276163^a^	20:66.52..66.53	48.62..38.66	5.85E-06-p2.32E-05
SC210	*ICE1*	rs41582170^b^	20:67.81	0.00	2.13E-05
SC210	*LOC101907249*	rs136535499^a^	20:69.05	240.87	2.13E-05
SC210	*EDARADD*	rs110746860^b^, rs110371081^b^, rs109902875^b^, rs110870694^b^, rs110610232^b^, rs134356559^b^, rs210911576^b^	28:9.14..28:9.16	0.00	6.71E-06-5.76E-05
AFC	*NXPH1*	rs133411648^a^	4:17.46	45.77	8.26E-07
AFC	*EXOC4*	rs110606254^a^	4:98.31	0.00	1.74E-06
AFC	*ZMAT4*	rs134390082^a^, rs137553882^a^, rs133519327^a^, rs135481346^a^	27:35.19..35.21	48.32..29.34	1.23E-05-3.18E-05
ASC	*FMN1*	rs134100268^b^	10:29.88	0.00	2.43E-06
ASC	*TMEM182*	rs43661848^a^	11:7.69	197.15	6.29E-06
ASC	*LOC790871*	rs136610615^a^	11:22.29	69.94	2.77E-06
ASC	*UBQLN3*	rs43031470^c^	15:48.70	0.00	3.39E-06

^a^intergenic region

^b^intron variant

^c^exon variant

Gene symbols, SNP reference number, and chromosomes (Chr) and positions (Pos, in megabase) were obtained from NCBI website. Distances to gene (kilobase) are presented from 5′ to gene direction. If distance equals zero (0.00), the SNP is on intragenic region. *P*-values are presented as the minimum (Min) and maximum (Max) significance obtained from the generalized quasi-likelihood method

**Table 2:** Highlighted genes from genome-wide (in bold) and chromosome-wise associations for scrotal circumference at 420 d of age

Symbol	SNP Reference	Chr:Pos	Distances to gene	*P*-value (Min - Max)
*RAP1B*	**rs110520377** ^a^, **rs133990240** ^a^, rs109547215^a^, **rs110160018** ^b^, **rs110261691** ^c^, **rs109099268** ^b^, **rs133124963** ^b^, **rs109023687** ^b^, **rs110034677** ^b^, **rs110091099** ^b^, **rs109950552** ^b^, **rs137658592** ^b^, rs133340933^b^, rs134626455^b^, rs110001336^b^, **rs109288126** ^b^, rs137319832^b^, **rs109506571** ^b^, **rs109248631** ^b^, **rs110027103** ^b^, **rs109561643** ^b^, **rs109210079** ^b^, **rs110160918** ^b^, rs134366426^b^, rs109273768^b^, rs109024096^b^, rs110798702^b^, **rs110625630** ^b^, **rs110219262** ^b^, **rs134311132** ^b^, **rs135497432** ^b^, **rs133173059** ^b^	5:45.35..45.40	8.58..0.00	2.47E-07 - 7.64E-05
*SRGAP1*	**rs110268648** ^b^, **rs109748105** ^b^, **rs134621421** ^b^	5:49.98..49.98	0.00	1.88E-06 - 6.55E-06
*FOXM1*	rs135705262^b^	5:107.39	0.00	4.00E-04
*TOP1*	**rs134822694** ^b^, **rs135287766** ^b^	13:70.39..70.40	0.00	3.86E-06
*STAU2*	rs137465376^b^, rs134711539^b^, rs137821036^a^	14:38.89..38.97	0.00..14.40	8.66E-05 - 5.00E-04
*PEX2*	rs137442228^d^, rs110035827^a^, rs41730291^a^	14:42.33..42.37	0.00..40.77	4.10E-04 - 9.80E-04
*FABP5*	rs109480456^a^, rs136045797^a^, rs133930486^e^, rs136613853^b^, rs137684819^c^, rs133054550^a^, rs133483556^a^, rs136236059^a^, rs136236059^a^, rs137344980^a^, rs135804214^a^, rs136785030^a^, rs135727060^a^, rs133436244^a^, rs134708967^a^, rs43103204^a^	14:46.63..46.73	15.48..0.00..83.31	1.55E-05 - 1.16E-03
*FABP12*	rs43765470^b^, rs43765465^a^, rs41730924^a^	14:46.89..46.92	0.00..28.04	3.48E-05 - 2.88E-04
*MED30*	rs135065691^c^, rs41734435^b^, rs135292147^a^	14:48.94..48.97	4.03..0.00..9.76	8.27E-04 - 9.48E-04
*TRHR*	rs133457508^e^	14:57.52	0.00	2.27E-04

^a^intergenic region ^b^intron variant ^c^downstream variant ^d^5′ UTR ^e^upstream variant

Gene symbols, SNP reference number, and chromosomes (Chr) and positions (Pos, in megabase) were obtained from NCBI website. Distances to gene (kilobase) are presented from 5′ to gene and 3′ to gene directions. If distance equals zero (0.00), the SNP is on intragenic region. *P*-values are presented as the minimum (Min) and maximum (Max) significance obtained from the generalized quasi-likelihood method

For SC210, chromosome-wise significantly associated SNPs (*P* ≤ 0.00001) were located on chromosomes 20 and 28. Five SNPs were located in the intergenic or intragenic regions of *SMIM23*, *PAPD7*, *ICE1*, and *EDARADD* genes, and non-coding RNA *LOC101907249* (Table 1 and Fig. 1a). On chromosome 28, seven SNPs were located in the *EDARADD* gene.

For SC420, chromosome-wise and genome-wise associations were observed on chromosomes 5, 9, 13, 14, 18, and 21 (Fig. 1b). The SNPs were close or within a total of 64 genes, 10 non-coding RNAs, 13 pseudogenes, and two transfer RNAs. Full information on SNPs, position, and genes are presented in the Additional files 1, 2, and 3.

**Fig. 2:**
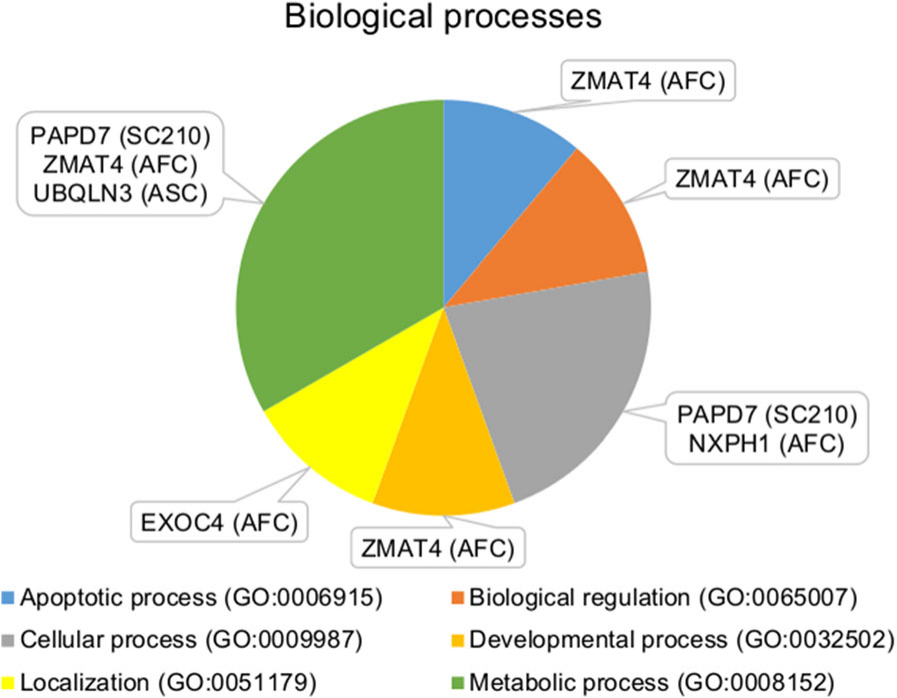
Biological processes for scrotal circumference (SC210) and age at first (AFC) and second (ASC) calving

Six significantly associated SNPs (*P* ≤ 0.00001) for AFC (Table 1 and Fig.1c) were located on chromosomes 4 (*NXPH1* and *EXOC4* genes) and 27 (*ZMAT4* gene). For ASC, four SNPs were significantly associated (*P* ≤ 0.000001) and located on chromosomes 10 (*FMN1* gene), 11 (*TMEM182* gene and *LOC790871* pseudogene), and 15 (*UBQLN3* gene). Biological processes for SC210, AFC, and ASC are presented in Fig. 2.

**Fig. 3:**
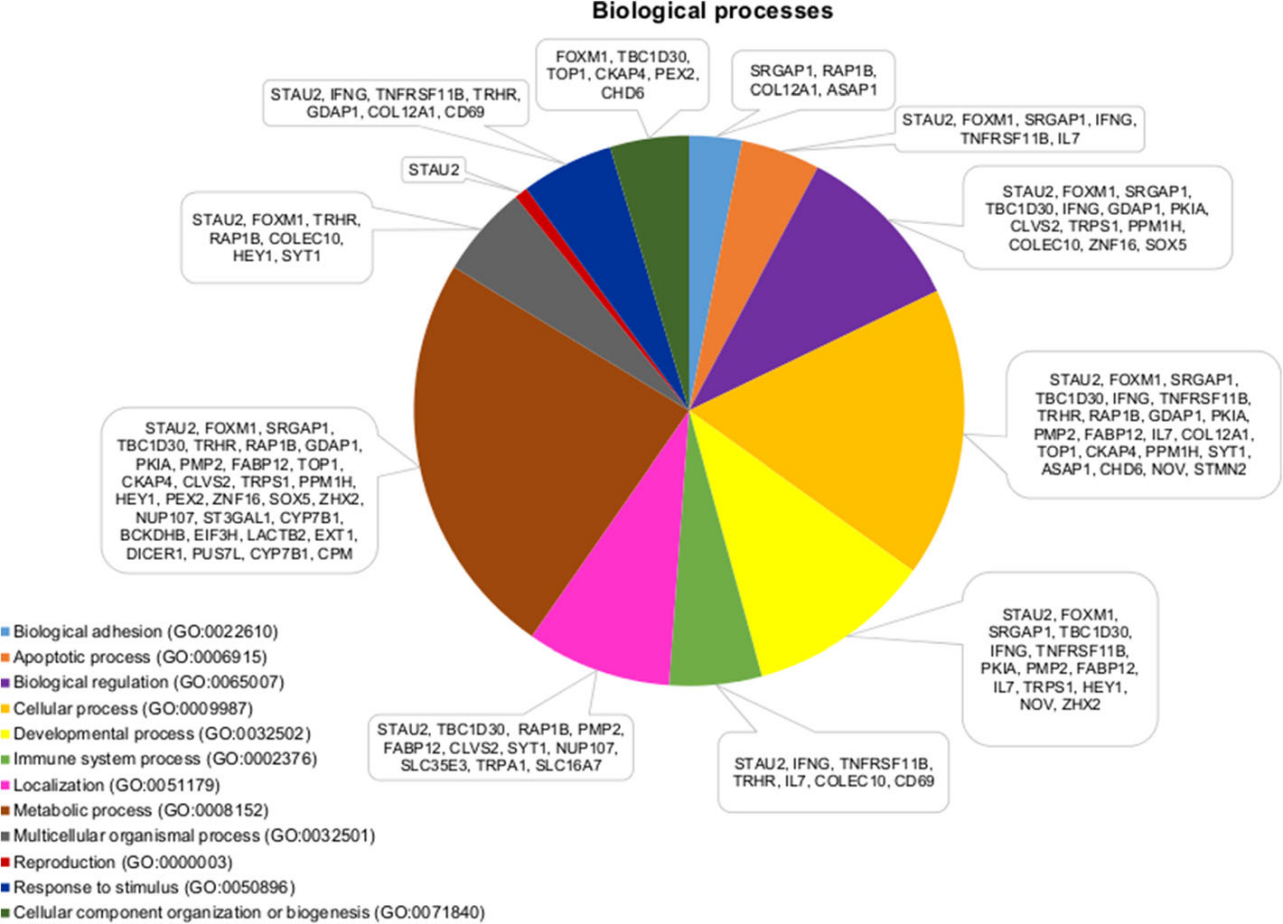
Biological processes for scrotal circumference at 420 d of age

**Fig. 4:**
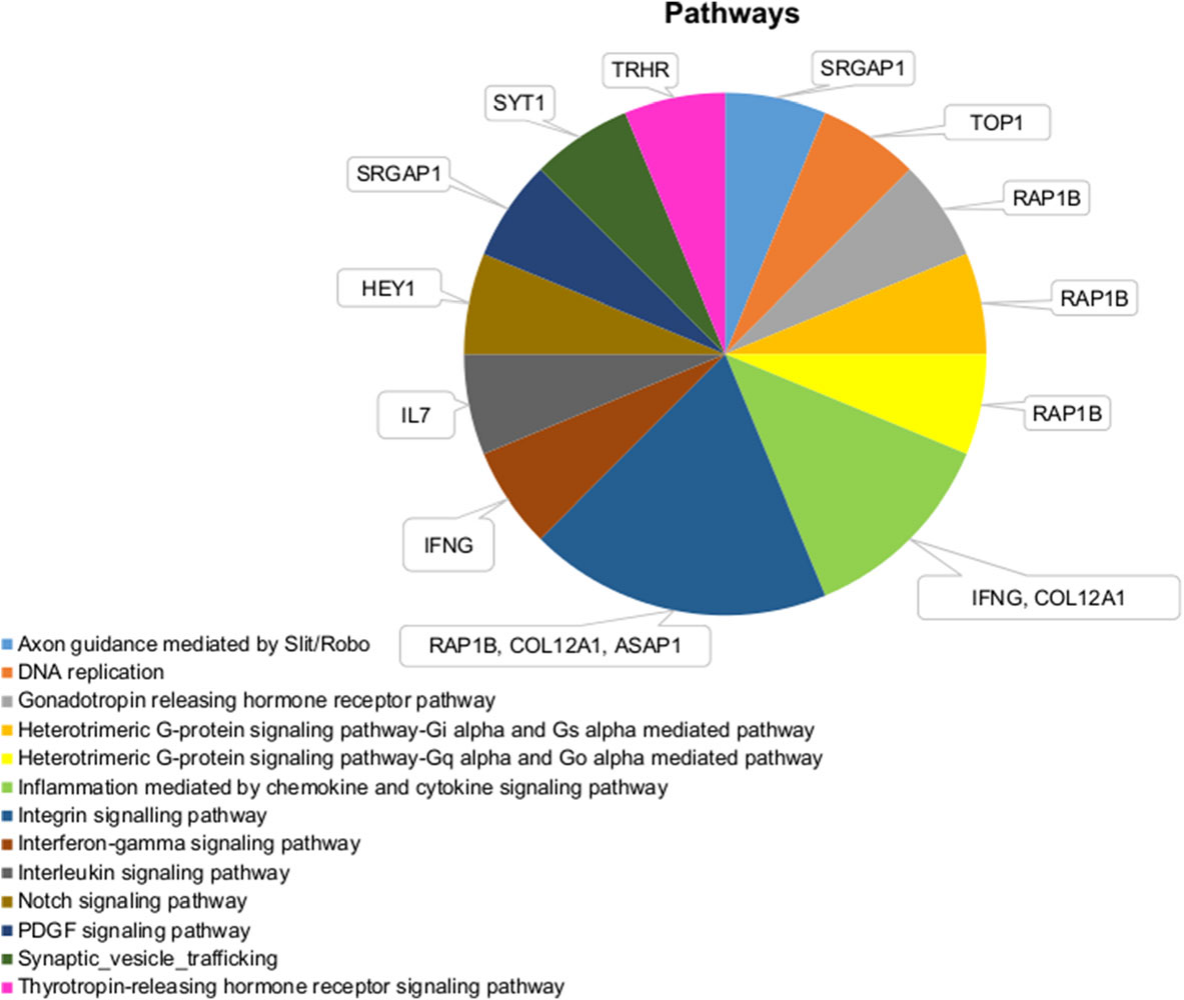
Pathways associations for scrotal circumference at 420 d of age

For SC420, biological processes and pathway associations are presented in Figs. 3 and 4, respectively. PANTHER tool was able to account for 62 genes over-represented among pathways when using *Bos taurus* as background. Most of the biological processes were involved in metabolic (32 genes), cellular (22 genes), developmental (14 genes), biological regulation (13 genes), and localization (11 genes) processes. Biological processes were observed for the immune system (seven genes), multicellular organismal process (seven genes), response to stimulus (seven genes), apoptotic process (six genes), cellular component organization or biogenesis (six genes), biological adhesion (four genes), and reproduction (one gene). Pathway analysis showed that the *RAP1B* gene is involved in four pathways, while the *IFNG*, *COL12A1*, and *SRGAP1* genes are involved in two pathways.

The average linkage disequilibrium for each trait by chromosome was equal to 0.70 (AFC - BTA27), 0.94 (SC210 – BTA20), 0.89 (SC210 – BTA28), 0.64 (SC420 – BTA5), 0.69 (SC420 – BTA9), 0.90 (SC420 – BTA13), 0.56 (SC420 – BTA14), 0.85 (SC420 – BTA18), and 0.99 (SC420 – BTA21). Some significant or suggestive regions presented r2 equal to zero. We highlighted the SC420 trait due to the higher number of regions presenting significant or suggestive associations (Additional file 4: Figs. S1, S2, S3 and S4).

## Discussion

The suggestive associations observed for SC210, AFC, and ASC, could provide an insight over potential regions responsible for the genetic variability of the traits. The main reason to consider FDR 10% was to study and describe, in a broader point of view, the biological pathways to aid in the comprehension of these complex traits. Furthermore, due to the polygenic inheritance of these traits, it was expected that many loci with small effects were responsible for expressing the phenotype (or EBV). In some cases, we observed the lack of information regarding the SNP or gene identified.

We observed high average linkage disequilibrium among significantly or suggestively associated SNPs for most of the traits studied; therefore, there is evidence of direct or indirect associations that could be affecting the traits. Mokry et al. [27], when studying this Canchim population, observed that the linkage disequilibrium estimated could be used for genomic selection and GWAS (minimum r^2^ varying from 0.33 to 0.40 up to 2.5 kb).

For SC210, we have found that the *SMIM23* gene is located inside QTL regions associated with the rate of non-return to estrus in Swedish Red and Swedish Holstein breed animals [32]. In this same region, McClure et al. [33] found QTL for scrotal circumference in American Angus cattle. Studies reported a QTL in the *PAPD7*, *ICE1*, and *LOC101907249* gene regions associated with percentage abnormal sperm in Holstein bulls [34] and calving ease in Angus breed cows [33]. The *EDARADD* gene participates in cell differentiation and mutations in this gene can result in abnormal development of tissues and organs of ectodermal origin [35]. In this region, a QTL associated with pregnancy rate trait have been reported by Boichard et al. [36]. No specific biological process for *SMIM23*, *ICE1*, and *LOC101907249* genes was observed in the literature.

Few suggestive associations were observed for SC210 and none of the SNPs associated with SC210 presented pleiotropic effect with SNP associated with SC420 (discussed below) or other studied traits (discussed as follows), which could be due to low genetic correlation among EBVs for these traits in our dataset.

Regarding the SC420 trait, we have identified the STAU2 gene, which participates in multiple biological processes (Table 2 and Fig. 3), highlighting reproduction, developmental and immune system. This gene was involved in muscle development [37] in *Bos taurus indicus*, *Bos taurus taurus*, and *Bos taurus taurus* x *Bos taurus indicus*. According to Ramayo-Caldas et al. [37], the *STAU2* gene was present in two main networks of *PPARGC1A* and *HNF4G* genes, which acts as transcription factors that activate a variety of hormone receptors [38] and binding to fatty acids [39]. Peddinti et al. [40] observed, through genetic network analysis, that one of the main biological networks for bovine germinal vesicle and bovine cumulus granulosa cell proteomes contain the *SRGAP1* and *TOP1* genes, respectively.

The *RAP1B* gene is involved in four pathways (Fig. 4), including gonadotropin-releasing hormone receptor pathway. This gene was also observed associated with cell-to-cell signaling network (integrin, ephrin receptor, and mitogen-activated protein kinase signaling network) [40]. The *MED30* and *TRHR* genes are strongly related to thyroid hormone, triggering hormonal processes associated with reproductive systems in males and females [41, 42].

Genes related to fatty acid, cholesterol, and triacylglycerol, such as *FABP5* [43], *FABP12* [44], *PEX2* [45], and *MED30* [46] are critical for energy and hormone production in males and females. Cholesterol is a precursor of steroid hormone production, such as testosterone, and therefore it is involved in male growth and reproductive development.

Important genome-wide associations on chromosome 14 for scrotal circumference were observed by Fortes et al. [5], demonstrating that this chromosome presents regions of interest that could be explored to identify sexually precocious animals. These authors observed associations with age at first corpus luteum in the same regions as scrotal circumference and have attributed their results to the genetic correlation between these traits, thus genes and SNPs associated with puberty in heifers were likely to be relevant for puberty in bulls, and vice versa. On chromosome 14, a large number of SNPs associated with puberty were identified in both bulls and heifers. Urbinati et al. [47] found important selection signatures in Canchim cattle on chromosomes 5 and 14, which were related to pigmentation (strongly selected trait in Charolais and Canchim), productive and reproductive traits.

Reports of QTL associated with reproductive traits of interest may give support to the results found in our study; however, most of the QTL were reported for female traits. On chromosome 5, QTL associated with the concentration of follicle-stimulating hormone in Brahman and Hereford crosses [48], have been described. The QTL were observed as associated with interval to first estrus after calving [7] and dystocia in dairy cattle [49] on chromosome 13. On chromosome 14, QTL associated with gestation period [50], number of inseminations per conception [51], and ovulation rate [52] have been reported.

As favorably correlated responses between scrotal circumference measures and age at first calving traits are expected through selection, whereas the genes previously reported could be highlighted as a candidate to, directly and indirectly, improve the reproductive performance of males and females, respectively. Moreover, fat deposition in cattle could be reflected in sexual precocity and carcass finishing, traits that have become the main concern of beef cattle breeders.

Few genes were observed across the positions of significant SNPs for AFC (Table 1 and Fig. 1c). According to Blaschek et al. [53], an association of the SNP rs110984522 (178.73 kb apart from the SNP rs133411648) was observed for sire fertility in Holstein breed. The *EXOC4* gene plays a role in insulin processing, metabolism of proteins, and peptide hormone metabolism pathways. Reports of QTL associated with scrotal circumference [33] have been described in this region. The *ZMAT4* gene, located on chromosome 27, participates in the apoptotic, biological, developmental, and metabolic processes. QTL associated with dystocia [49] and calving ease [54] in Holstein cattle and non-return rate [55] in Angus cattle have been described.

Significant SNPs for ASC are presented in Table 1 and Fig. 1d. The *FMN1* gene located on chromosome 10 participates in actin cytoskeleton organization and is of great importance for cell and muscle movements [56]. QTL associated with calving ease have been observed in this region [33].

A QTL associated with scrotal circumference was observed in the region of the *TMEM182* gene [33]. No function or biological processes have been described for the *LOC790871* and *TMEM182* genes in the literature or databases consulted. In the region comprising the *LOC790871* gene, QTL regions have been reported associated with the following traits: scrotal circumference [33], subcutaneous fat [57], and sperm motility [34] in Angus, Holstein, and Charolais × Holstein crossbred cattle, respectively.

It has been verified that the *UBQLN3* gene is expressed in the testes, acts in spermatogenesis in humans and rats [58] and is conserved in mammals (*Homo sapiens*, *Mus musculus*, *Rattus norvegicus*, *Canis lupus familiaris*, and *Bos taurus*). This gene is involved in the protein processing in endoplasmic reticulum pathway. A QTL associated with weight and carcass traits has been described [33] in the region in which the UBQLN3 gene is located.

## Conclusions

Few associations were observed for SC210, AFC, and ASC, reinforcing their polygenic inheritance and the complexity of understanding the genetic architecture of reproductive traits. Finding many associations for SC420 in various regions of the Canchim genome also reveals the difficulty of targeting specific candidate genes that could act on fertility; nonetheless, the high linkage disequilibrium between loci herein estimated could aid to overcome this issue. Therefore, all relevant information about genomic regions influencing reproductive traits may contribute to target candidate genes for further investigation of causal mutations and aid in future genomic studies in Canchim cattle to improve the breeding program.

## Additional files

Additional file 1: Significantly associated single nucleotide polymorphism (SNP) with scrotal circumference at 420 days of age (SC420) after Bonferroni (B) and false discovery rate (FDR) correction at a chromosome-wise (CW) and genome-wide (GW) level. (DOCX 95 kb)

Additional file 2: Genome-wide (in bold) and chromosome-wise associations for scrotal circumference at 420 days of age. Gene symbols, SNP reference number, and chromosomes (Chr) and positions (Pos, in megabase) were obtained from NCBI website. Distances to gene (kilobase) are presented from 5′ to gene and 3′ to gene directions. If distance equals zero (0.00), the SNP is on intragenic region. *P*-values are presented as the minimum (Min) and maximum (Max) significance obtained from the generalized quasi-likelihood method. (DOCX 34 kb)

Additional file 3: Candidate genes identified for scrotal circumference at 420 days of age. (DOCX 26 kb)

Additional file 4: Fig. S1. Linkage disequilibrium for scrotal circumference at 420 d of age on chromosome 5. **Fig. S2**. Linkage disequilibrium for scrotal circumference at 420 d of age on chromosome 9. **Fig. S3.** Linkage disequilibrium for scrotal circumference at 420 d of age on chromosome 13. **Fig. S4.** Linkage disequilibrium for scrotal circumference at 420 d of age on chromosome 14. **Fig. S5.** Linkage disequilibrium for scrotal circumference at 420 d of age on chromosome 18. **Fig. S6.** Linkage disequilibrium for scrotal circumference at 420 d of age on chromosome 21. (ZIP 1985 kb)

## Abbreviations

AFC: Age at first calving
ASC: Age at second calving
BTA: *Bos taurus* chromosome
EBV: Estimated breeding value
FDR: False discovery rate
GQLS: Generalized Quasi-Likelihood Score
GWAS: Genome-wide association study
QC: Quality control
QTL: Quantitative trait loci
r^2^: Linkage disequilibrium measure
SC210: Scrotal circumference at 210 days of age
SC420: Scrotal circumference at 420 days of age
SNP: Single nucleotide polymorphism
