# Supplementary material for: Candidate genes for male and female reproductive traits in Canchim beef cattle
Source: J Anim Sci Biotechnol. 2017 Aug 23;8:67. doi: 10.1186/s40104-017-0199-8 (PMC5569548; doi:10.1186/s40104-017-0199-8)
Supplement: Supplementary file 1 — Significantly associated single nucleotide polymorphism (SNP) with scrotal circumference at 420 days of age (SC420) after Bonferroni (B) and false discovery rate (FDR) correction at a chromosome-wise (CW) and genome-wide (GW) level. (DOCX 95 kb) [file 40104_2017_199_MOESM1_ESM.docx]

**Additional files**

**Additional file 1.** Significantly associated single nucleotide polymorphism (SNP) with scrotal circumference at 420 days of age (SC420) after Bonferroni (B) and false discovery rate (FDR) correction at a chromosome-wise (CW) and genome-wide (GW) level.

| **SNP Name** | **BTA** | **Position** | **SNP Reference** | **MAF** | ***P*-value** | **B_CW** | **B_GW** | **FDR_CW** | **FDR_GW** |
| --- | --- | --- | --- | --- | --- | --- | --- | --- | --- |
| BovineHD0500002567 | 5 | 8986354 | [rs43429788](http://www.ncbi.nlm.nih.gov/projects/SNP/snp_ref.cgi?rs=43429788) | 0.695153 | 9.45E-05 | ns | ns | s5 | ns |
| BovineHD0500010587 | 5 | 36934565 | [rs43434300](http://www.ncbi.nlm.nih.gov/projects/SNP/snp_ref.cgi?rs=43434300) | 0.544643 | 1.01E-04 | ns | ns | s5 | ns |
| BovineHD0500012069 | 5 | 42218286 | [rs133344101](http://www.ncbi.nlm.nih.gov/projects/SNP/snp_ref.cgi?rs=133344101) | 0.341432 | 3.60E-04 | ns | ns | s10 | ns |
| BovineHD0500012080 | 5 | 42255407 | [rs137418499](http://www.ncbi.nlm.nih.gov/projects/SNP/snp_ref.cgi?rs=137418499) | 0.66199 | 2.05E-04 | ns | ns | s10 | ns |
| BovineHD0500012081 | 5 | 42256503 | [rs132691450](http://www.ncbi.nlm.nih.gov/projects/SNP/snp_ref.cgi?rs=132691450) | 0.33801 | 2.05E-04 | ns | ns | s10 | ns |
| BovineHD0500012082 | 5 | 42257376 | [rs134381968](http://www.ncbi.nlm.nih.gov/projects/SNP/snp_ref.cgi?rs=134381968) | 0.662404 | 1.86E-04 | ns | ns | s10 | ns |
| BovineHD0500012083 | 5 | 42258892 | [rs135563280](http://www.ncbi.nlm.nih.gov/projects/SNP/snp_ref.cgi?rs=135563280) | 0.66199 | 2.05E-04 | ns | ns | s10 | ns |
| BovineHD0500012083 | 5 | 42265135 | [rs135563280](http://www.ncbi.nlm.nih.gov/projects/SNP/snp_ref.cgi?rs=135563280) | 0.693095 | 3.31E-04 | ns | ns | s10 | ns |
| BovineHD0500012089 | 5 | 42266360 | [rs110587794](http://www.ncbi.nlm.nih.gov/projects/SNP/snp_ref.cgi?rs=110587794) | 0.305627 | 3.30E-04 | ns | ns | s10 | ns |
| BovineHD0500012962 | 5 | 45102460 | [rs134557648](http://www.ncbi.nlm.nih.gov/projects/SNP/snp_ref.cgi?rs=134557648) | 0.163683 | 1.65E-05 | ns | ns | s5 | ns |
| BovineHD0500012963 | 5 | 45104529 | [rs136126069](http://www.ncbi.nlm.nih.gov/projects/SNP/snp_ref.cgi?rs=136126069) | 0.126276 | 2.18E-04 | ns | ns | s10 | ns |
| BovineHD0500013016 | 5 | 45247118 | [rs136466664](http://www.ncbi.nlm.nih.gov/projects/SNP/snp_ref.cgi?rs=136466664) | 0.381378 | 2.91E-04 | ns | ns | s10 | ns |
| BovineHD0500013018 | 5 | 45249863 | [rs134969150](http://www.ncbi.nlm.nih.gov/projects/SNP/snp_ref.cgi?rs=134969150) | 0.741071 | 1.49E-04 | ns | ns | s10 | ns |
| BovineHD0500013019 | 5 | 45250721 | [rs136251215](http://www.ncbi.nlm.nih.gov/projects/SNP/snp_ref.cgi?rs=136251215) | 0.617347 | 3.01E-04 | ns | ns | s10 | ns |
| BovineHD0500013020 | 5 | 45252150 | [rs136974465](http://www.ncbi.nlm.nih.gov/projects/SNP/snp_ref.cgi?rs=136974465) | 0.258929 | 1.49E-04 | ns | ns | s10 | ns |
| BovineHD0500013021 | 5 | 45253044 | [rs134653913](http://www.ncbi.nlm.nih.gov/projects/SNP/snp_ref.cgi?rs=134653913) | 0.610966 | 5.67E-05 | ns | ns | s5 | ns |
| BovineHD0500013022 | 5 | 45254535 | [rs137721339](http://www.ncbi.nlm.nih.gov/projects/SNP/snp_ref.cgi?rs=137721339) | 0.382653 | 3.01E-04 | ns | ns | s10 | ns |
| BovineHD0500013023 | 5 | 45255801 | [rs133985145](http://www.ncbi.nlm.nih.gov/projects/SNP/snp_ref.cgi?rs=133985145) | 0.382051 | 1.97E-04 | ns | ns | s10 | ns |
| BovineHD0500013024 | 5 | 45257171 | [rs135251296](http://www.ncbi.nlm.nih.gov/projects/SNP/snp_ref.cgi?rs=135251296) | 0.618622 | 2.91E-04 | ns | ns | s10 | ns |
| BovineHD0500013025 | 5 | 45258841 | [rs132874802](http://www.ncbi.nlm.nih.gov/projects/SNP/snp_ref.cgi?rs=132874802) | 0.429847 | 8.17E-06 | ns | ns | s1 | s10 |
| BovineHD0500013026 | 5 | 45260223 | [rs109156482](http://www.ncbi.nlm.nih.gov/projects/SNP/snp_ref.cgi?rs=109156482) | 0.429847 | 8.17E-06 | ns | ns | s1 | s10 |
| BovineHD0500013028 | 5 | 45267821 | [rs110291739](http://www.ncbi.nlm.nih.gov/projects/SNP/snp_ref.cgi?rs=110291739) | 0.178571 | 6.37E-05 | ns | ns | s5 | ns |
| BovineHD0500013029 | 5 | 45274417 | [rs110572792](http://www.ncbi.nlm.nih.gov/projects/SNP/snp_ref.cgi?rs=110572792) | 0.178571 | 6.37E-05 | ns | ns | s5 | ns |
| BovineHD0500013031 | 5 | 45282131 | [rs110698350](http://www.ncbi.nlm.nih.gov/projects/SNP/snp_ref.cgi?rs=110698350) | 0.156888 | 3.89E-04 | ns | ns | s10 | ns |
| BovineHD0500013032 | 5 | 45283704 | [rs134370690](http://www.ncbi.nlm.nih.gov/projects/SNP/snp_ref.cgi?rs=134370690) | 0.714286 | 9.24E-05 | ns | ns | s5 | ns |
| BovineHD0500013033 | 5 | 45289852 | [rs109284796](http://www.ncbi.nlm.nih.gov/projects/SNP/snp_ref.cgi?rs=109284796) | 0.695312 | 5.66E-06 | ns | ns | s1 | s10 |
| BovineHD0500013034 | 5 | 45293077 | [rs110852214](http://www.ncbi.nlm.nih.gov/projects/SNP/snp_ref.cgi?rs=110852214) | 0.843112 | 3.89E-04 | ns | ns | s10 | ns |
| BovineHD0500013036 | 5 | 45297244 | [rs137552662](http://www.ncbi.nlm.nih.gov/projects/SNP/snp_ref.cgi?rs=137552662) | 0.651786 | 7.33E-05 | ns | ns | s5 | ns |
| BovineHD0500013037 | 5 | 45305082 | [rs108983599](http://www.ncbi.nlm.nih.gov/projects/SNP/snp_ref.cgi?rs=108983599) | 0.691327 | 5.59E-06 | ns | ns | s1 | s10 |
| Hapmap43728-BTA-94000 | 5 | 45306060 | [rs41595547](http://www.ncbi.nlm.nih.gov/projects/SNP/snp_ref.cgi?rs=41595547) | 0.653453 | 9.06E-05 | ns | ns | s5 | ns |
| BovineHD0500013038 | 5 | 45308609 | [rs137318696](http://www.ncbi.nlm.nih.gov/projects/SNP/snp_ref.cgi?rs=137318696) | 0.346753 | 8.12E-05 | ns | ns | s5 | ns |
| BovineHD0500013042 | 5 | 45321266 | [rs134463406](http://www.ncbi.nlm.nih.gov/projects/SNP/snp_ref.cgi?rs=134463406) | 0.651786 | 7.33E-05 | ns | ns | s5 | ns |
| BovineHD0500013045 | 5 | 45331558 | [rs133818805](http://www.ncbi.nlm.nih.gov/projects/SNP/snp_ref.cgi?rs=133818805) | 0.706941 | 1.16E-04 | ns | ns | s5 | ns |
| BovineHD0500013048 | 5 | 45346713 | [rs110520377](http://www.ncbi.nlm.nih.gov/projects/SNP/snp_ref.cgi?rs=110520377) | 0.589286 | 3.18E-06 | ns | ns | s1 | s5 |
| BovineHD0500013049 | 5 | 45352128 | [rs133990240](http://www.ncbi.nlm.nih.gov/projects/SNP/snp_ref.cgi?rs=133990240) | 0.670918 | 1.42E-06 | s5 | ns | s1 | s5 |
| BovineHD0500013050 | 5 | 45352728 | [rs109547215](http://www.ncbi.nlm.nih.gov/projects/SNP/snp_ref.cgi?rs=109547215) | 0.364796 | 1.81E-05 | ns | ns | s5 | ns |
| BovineHD0500013051 | 5 | 45354729 | [rs110261691](http://www.ncbi.nlm.nih.gov/projects/SNP/snp_ref.cgi?rs=110261691) | 0.670918 | 1.42E-06 | s5 | ns | s1 | s5 |
| BovineHD0500013052 | 5 | 45356833 | [rs110160018](http://www.ncbi.nlm.nih.gov/projects/SNP/snp_ref.cgi?rs=110160018) | 0.373724 | 2.47E-07 | s1 | ns | s1 | s5 |
| BovineHD0500013053 | 5 | 45357517 | [rs109099268](http://www.ncbi.nlm.nih.gov/projects/SNP/snp_ref.cgi?rs=109099268) | 0.670918 | 1.42E-06 | s5 | ns | s1 | s5 |
| BovineHD0500013055 | 5 | 45359590 | [rs133124963](http://www.ncbi.nlm.nih.gov/projects/SNP/snp_ref.cgi?rs=133124963) | 0.589514 | 2.98E-06 | s10 | ns | s1 | s5 |
| BovineHD0500013056 | 5 | 45360390 | [rs109023687](http://www.ncbi.nlm.nih.gov/projects/SNP/snp_ref.cgi?rs=109023687) | 0.586563 | 6.24E-06 | ns | ns | s1 | s10 |
| BovineHD0500013057 | 5 | 45364205 | [rs110034677](http://www.ncbi.nlm.nih.gov/projects/SNP/snp_ref.cgi?rs=110034677) | 0.329082 | 1.42E-06 | s5 | ns | s1 | s5 |
| BovineHD0500013058 | 5 | 45365062 | [rs110091099](http://www.ncbi.nlm.nih.gov/projects/SNP/snp_ref.cgi?rs=110091099) | 0.670918 | 1.42E-06 | s5 | ns | s1 | s5 |
| BovineHD0500013059 | 5 | 45365842 | [rs109950552](http://www.ncbi.nlm.nih.gov/projects/SNP/snp_ref.cgi?rs=109950552) | 0.659439 | 5.68E-07 | s5 | ns | s1 | s5 |
| BovineHD0500013060 | 5 | 45367028 | [rs137658592](http://www.ncbi.nlm.nih.gov/projects/SNP/snp_ref.cgi?rs=137658592) | 0.329082 | 1.42E-06 | s5 | ns | s1 | s5 |
| BovineHD0500013061 | 5 | 45368209 | [rs133340933](http://www.ncbi.nlm.nih.gov/projects/SNP/snp_ref.cgi?rs=133340933) | 0.622449 | 3.81E-05 | ns | ns | s5 | ns |
| BovineHD0500013062 | 5 | 45368789 | [rs134626455](http://www.ncbi.nlm.nih.gov/projects/SNP/snp_ref.cgi?rs=134626455) | 0.753827 | 2.76E-05 | ns | ns | s5 | ns |
| BovineHD0500013063 | 5 | 45371857 | [rs110001336](http://www.ncbi.nlm.nih.gov/projects/SNP/snp_ref.cgi?rs=110001336) | 0.434949 | 1.16E-05 | ns | ns | s5 | ns |
| BovineHD0500013064 | 5 | 45373404 | [rs109288126](http://www.ncbi.nlm.nih.gov/projects/SNP/snp_ref.cgi?rs=109288126) | 0.670918 | 1.42E-06 | s5 | ns | s1 | s5 |
| BovineHD0500013065 | 5 | 45374099 | [rs137319832](http://www.ncbi.nlm.nih.gov/projects/SNP/snp_ref.cgi?rs=137319832) | 0.622449 | 3.81E-05 | ns | ns | s5 | ns |
| BovineHD0500013066 | 5 | 45374760 | [rs109506571](http://www.ncbi.nlm.nih.gov/projects/SNP/snp_ref.cgi?rs=109506571) | 0.670918 | 1.42E-06 | s5 | ns | s1 | s5 |
| BovineHD0500013067 | 5 | 45377693 | [rs109248631](http://www.ncbi.nlm.nih.gov/projects/SNP/snp_ref.cgi?rs=109248631) | 0.589286 | 3.18E-06 | ns | ns | s1 | s5 |
| BovineHD0500013069 | 5 | 45380271 | [rs110027103](http://www.ncbi.nlm.nih.gov/projects/SNP/snp_ref.cgi?rs=110027103) | 0.329082 | 1.42E-06 | s5 | ns | s1 | s5 |
| BovineHD0500013070 | 5 | 45381502 | [rs109561643](http://www.ncbi.nlm.nih.gov/projects/SNP/snp_ref.cgi?rs=109561643) | 0.589286 | 3.18E-06 | ns | ns | s1 | s5 |
| BovineHD0500013071 | 5 | 45382190 | [rs109210079](http://www.ncbi.nlm.nih.gov/projects/SNP/snp_ref.cgi?rs=109210079) | 0.589286 | 3.18E-06 | ns | ns | s1 | s5 |
| BovineHD0500013072 | 5 | 45383111 | [rs110160918](http://www.ncbi.nlm.nih.gov/projects/SNP/snp_ref.cgi?rs=110160918) | 0.692308 | 2.70E-06 | s10 | ns | s1 | s5 |
| BovineHD0500013073 | 5 | 45384721 | [rs134366426](http://www.ncbi.nlm.nih.gov/projects/SNP/snp_ref.cgi?rs=134366426) | 0.27551 | 7.64E-05 | ns | ns | s5 | ns |
| BovineHD0500013074 | 5 | 45386660 | [rs109273768](http://www.ncbi.nlm.nih.gov/projects/SNP/snp_ref.cgi?rs=109273768) | 0.27551 | 7.64E-05 | ns | ns | s5 | ns |
| BovineHD0500013075 | 5 | 45387612 | [rs109024096](http://www.ncbi.nlm.nih.gov/projects/SNP/snp_ref.cgi?rs=109024096) | 0.72449 | 7.64E-05 | ns | ns | s5 | ns |
| BovineHD0500013076 | 5 | 45390164 | [rs110798702](http://www.ncbi.nlm.nih.gov/projects/SNP/snp_ref.cgi?rs=110798702) | 0.320153 | 2.98E-05 | ns | ns | s5 | ns |
| BovineHD0500013077 | 5 | 45391371 | [rs110625630](http://www.ncbi.nlm.nih.gov/projects/SNP/snp_ref.cgi?rs=110625630) | 0.457908 | 1.06E-06 | s5 | ns | s1 | s5 |
| BovineHD0500013078 | 5 | 45392301 | [rs110219262](http://www.ncbi.nlm.nih.gov/projects/SNP/snp_ref.cgi?rs=110219262) | 0.542092 | 1.06E-06 | s5 | ns | s1 | s5 |
| BovineHD0500013079 | 5 | 45393775 | [rs134311132](http://www.ncbi.nlm.nih.gov/projects/SNP/snp_ref.cgi?rs=134311132) | 0.453846 | 4.80E-07 | s5 | ns | s1 | s5 |
| BovineHD0500013080 | 5 | 45394458 | [rs135497432](http://www.ncbi.nlm.nih.gov/projects/SNP/snp_ref.cgi?rs=135497432) | 0.546036 | 5.80E-07 | s5 | ns | s1 | s5 |
| BovineHD0500013081 | 5 | 45395358 | [rs133173059](http://www.ncbi.nlm.nih.gov/projects/SNP/snp_ref.cgi?rs=133173059) | 0.453846 | 7.25E-07 | s5 | ns | s1 | s5 |
| BovineHD0500013199 | 5 | 45880424 | [rs109941119](http://www.ncbi.nlm.nih.gov/projects/SNP/snp_ref.cgi?rs=109941119) | 0.71301 | 1.73E-04 | ns | ns | s10 | ns |
| BovineHD0500013201 | 5 | 45897449 | [rs110443290](http://www.ncbi.nlm.nih.gov/projects/SNP/snp_ref.cgi?rs=110443290) | 0.71301 | 1.73E-04 | ns | ns | s10 | ns |
| BovineHD0500013202 | 5 | 45899169 | [rs110739783](http://www.ncbi.nlm.nih.gov/projects/SNP/snp_ref.cgi?rs=110739783) | 0.28699 | 1.73E-04 | ns | ns | s10 | ns |
| BovineHD0500013203 | 5 | 45901967 | [rs133006321](http://www.ncbi.nlm.nih.gov/projects/SNP/snp_ref.cgi?rs=133006321) | 0.28699 | 1.73E-04 | ns | ns | s10 | ns |
| BovineHD0500013206 | 5 | 45921555 | [rs136589101](http://www.ncbi.nlm.nih.gov/projects/SNP/snp_ref.cgi?rs=136589101) | 0.28699 | 1.73E-04 | ns | ns | s10 | ns |
| Hapmap38983-BTA-73489 | 5 | 45926002 | [rs41590069](http://www.ncbi.nlm.nih.gov/projects/SNP/snp_ref.cgi?rs=41590069) | 0.715026 | 4.09E-04 | ns | ns | s10 | ns |
| BovineHD0500013209 | 5 | 45940813 | [rs109635265](http://www.ncbi.nlm.nih.gov/projects/SNP/snp_ref.cgi?rs=109635265) | 0.71301 | 1.73E-04 | ns | ns | s10 | ns |
| BovineHD0500013210 | 5 | 45943818 | [rs109461229](http://www.ncbi.nlm.nih.gov/projects/SNP/snp_ref.cgi?rs=109461229) | 0.71301 | 1.73E-04 | ns | ns | s10 | ns |
| BovineHD0500013215 | 5 | 45969746 | [rs110502555](http://www.ncbi.nlm.nih.gov/projects/SNP/snp_ref.cgi?rs=110502555) | 0.71301 | 1.73E-04 | ns | ns | s10 | ns |
| BovineHD0500013219 | 5 | 45988592 | [rs109042529](http://www.ncbi.nlm.nih.gov/projects/SNP/snp_ref.cgi?rs=109042529) | 0.71301 | 1.73E-04 | ns | ns | s10 | ns |
| BovineHD0500013855 | 5 | 47921750 | [rs137227247](http://www.ncbi.nlm.nih.gov/projects/SNP/snp_ref.cgi?rs=137227247) | 0.110969 | 2.85E-05 | ns | ns | s5 | ns |
| BovineHD0500014093 | 5 | 48927617 | [rs135293584](http://www.ncbi.nlm.nih.gov/projects/SNP/snp_ref.cgi?rs=135293584) | 0.517903 | 2.12E-04 | ns | ns | s10 | ns |
| BovineHD0500014121 | 5 | 49038495 | [rs134846637](http://www.ncbi.nlm.nih.gov/projects/SNP/snp_ref.cgi?rs=134846637) | 0.507653 | 2.08E-04 | ns | ns | s10 | ns |
| BovineHD0500014141 | 5 | 49100257 | [rs109818155](http://www.ncbi.nlm.nih.gov/projects/SNP/snp_ref.cgi?rs=109818155) | 0.645408 | 3.78E-04 | ns | ns | s10 | ns |
| BovineHD0500014142 | 5 | 49102470 | [rs109388550](http://www.ncbi.nlm.nih.gov/projects/SNP/snp_ref.cgi?rs=109388550) | 0.354592 | 3.78E-04 | ns | ns | s10 | ns |
| BovineHD0500014383 | 5 | 49978817 | [rs110268648](http://www.ncbi.nlm.nih.gov/projects/SNP/snp_ref.cgi?rs=110268648) | 0.774235 | 3.13E-06 | s10 | ns | s1 | s5 |
| BovineHD0500014384 | 5 | 49979883 | [rs109748105](http://www.ncbi.nlm.nih.gov/projects/SNP/snp_ref.cgi?rs=109748105) | 0.752551 | 1.88E-06 | s10 | ns | s1 | s5 |
| BovineHD0500014386 | 5 | 49984175 | [rs134621421](http://www.ncbi.nlm.nih.gov/projects/SNP/snp_ref.cgi?rs=134621421) | 0.825255 | 6.55E-06 | ns | ns | s1 | s10 |
| BovineHD0500014470 | 5 | 50321156 | [rs137166674](http://www.ncbi.nlm.nih.gov/projects/SNP/snp_ref.cgi?rs=137166674) | 0.792526 | 4.50E-05 | ns | ns | s5 | ns |
| BovineHD0500014473 | 5 | 50327258 | [rs134596971](http://www.ncbi.nlm.nih.gov/projects/SNP/snp_ref.cgi?rs=134596971) | 0.885204 | 1.09E-04 | ns | ns | s5 | ns |
| BovineHD0500014476 | 5 | 50336207 | [rs109404421](http://www.ncbi.nlm.nih.gov/projects/SNP/snp_ref.cgi?rs=109404421) | 0.118622 | 2.07E-04 | ns | ns | s10 | ns |
| BovineHD0500014477 | 5 | 50344640 | [rs137735429](http://www.ncbi.nlm.nih.gov/projects/SNP/snp_ref.cgi?rs=137735429) | 0.21301 | 4.10E-04 | ns | ns | s10 | ns |
| BovineHD0500014479 | 5 | 50348439 | [rs137317884](http://www.ncbi.nlm.nih.gov/projects/SNP/snp_ref.cgi?rs=137317884) | 0.26148 | 1.46E-04 | ns | ns | s10 | ns |
| BovineHD0500014481 | 5 | 50351917 | [rs136285326](http://www.ncbi.nlm.nih.gov/projects/SNP/snp_ref.cgi?rs=136285326) | 0.262148 | 1.45E-04 | ns | ns | s10 | ns |
| BovineHD0500014482 | 5 | 50352942 | [rs133604447](http://www.ncbi.nlm.nih.gov/projects/SNP/snp_ref.cgi?rs=133604447) | 0.78699 | 4.10E-04 | ns | ns | s10 | ns |
| BovineHD0500014594 | 5 | 50810912 | [rs110034898](http://www.ncbi.nlm.nih.gov/projects/SNP/snp_ref.cgi?rs=110034898) | 0.753866 | 2.88E-04 | ns | ns | s10 | ns |
| BovineHD0500015038 | 5 | 52354830 | [rs133712981](http://www.ncbi.nlm.nih.gov/projects/SNP/snp_ref.cgi?rs=133712981) | 0.792092 | 1.87E-04 | ns | ns | s10 | ns |
| BovineHD0500015049 | 5 | 52399389 | [rs133468722](http://www.ncbi.nlm.nih.gov/projects/SNP/snp_ref.cgi?rs=133468722) | 0.792092 | 1.87E-04 | ns | ns | s10 | ns |
| BovineHD0500015055 | 5 | 52421840 | [rs108942252](http://www.ncbi.nlm.nih.gov/projects/SNP/snp_ref.cgi?rs=108942252) | 0.207908 | 1.87E-04 | ns | ns | s10 | ns |
| BovineHD0500015073 | 5 | 52493709 | [rs110821453](http://www.ncbi.nlm.nih.gov/projects/SNP/snp_ref.cgi?rs=110821453) | 0.107417 | 4.64E-05 | ns | ns | s5 | ns |
| BovineHD0500015089 | 5 | 52570949 | [rs137102880](http://www.ncbi.nlm.nih.gov/projects/SNP/snp_ref.cgi?rs=137102880) | 0.515345 | 3.62E-04 | ns | ns | s10 | ns |
| BovineHD0500015106 | 5 | 52636182 | [rs133590688](http://www.ncbi.nlm.nih.gov/projects/SNP/snp_ref.cgi?rs=133590688) | 0.869898 | 4.35E-06 | ns | ns | s1 | s10 |
| BovineHD0500015107 | 5 | 52643407 | [rs109729395](http://www.ncbi.nlm.nih.gov/projects/SNP/snp_ref.cgi?rs=109729395) | 0.816327 | 6.71E-05 | ns | ns | s5 | ns |
| BovineHD0500015108 | 5 | 52646959 | [rs109494203](http://www.ncbi.nlm.nih.gov/projects/SNP/snp_ref.cgi?rs=109494203) | 0.814433 | 5.19E-05 | ns | ns | s5 | ns |
| BovineHD0500015109 | 5 | 52648467 | [rs109103723](http://www.ncbi.nlm.nih.gov/projects/SNP/snp_ref.cgi?rs=109103723) | 0.816327 | 6.71E-05 | ns | ns | s5 | ns |
| BovineHD0500015110 | 5 | 52651133 | [rs109036488](http://www.ncbi.nlm.nih.gov/projects/SNP/snp_ref.cgi?rs=109036488) | 0.182051 | 6.37E-05 | ns | ns | s5 | ns |
| BovineHD0500015111 | 5 | 52653581 | [rs132720145](http://www.ncbi.nlm.nih.gov/projects/SNP/snp_ref.cgi?rs=132720145) | 0.183673 | 6.71E-05 | ns | ns | s5 | ns |
| BovineHD0500015113 | 5 | 52656379 | [rs135762369](http://www.ncbi.nlm.nih.gov/projects/SNP/snp_ref.cgi?rs=135762369) | 0.816327 | 6.71E-05 | ns | ns | s5 | ns |
| BovineHD0500015130 | 5 | 52755751 | [rs110654445](http://www.ncbi.nlm.nih.gov/projects/SNP/snp_ref.cgi?rs=110654445) | 0.575255 | 4.84E-05 | ns | ns | s5 | ns |
| BovineHD0500015131 | 5 | 52756327 | [rs132843988](http://www.ncbi.nlm.nih.gov/projects/SNP/snp_ref.cgi?rs=132843988) | 0.517857 | 2.45E-06 | s10 | ns | s1 | s5 |
| BovineHD0500015132 | 5 | 52759020 | [rs134781821](http://www.ncbi.nlm.nih.gov/projects/SNP/snp_ref.cgi?rs=134781821) | 0.517857 | 2.45E-06 | s10 | ns | s1 | s5 |
| BovineHD0500015133 | 5 | 52759987 | [rs110197988](http://www.ncbi.nlm.nih.gov/projects/SNP/snp_ref.cgi?rs=110197988) | 0.575448 | 4.50E-05 | ns | ns | s5 | ns |
| BovineHD0500015135 | 5 | 52765006 | [rs110906264](http://www.ncbi.nlm.nih.gov/projects/SNP/snp_ref.cgi?rs=110906264) | 0.517857 | 2.45E-06 | s10 | ns | s1 | s5 |
| ARS-BFGL-NGS-78314 | 5 | 52771740 | [rs109124151](http://www.ncbi.nlm.nih.gov/projects/SNP/snp_ref.cgi?rs=109124151) | 0.405928 | 1.40E-04 | ns | ns | s10 | ns |
| BovineHD0500015137 | 5 | 52775407 | [rs110767141](http://www.ncbi.nlm.nih.gov/projects/SNP/snp_ref.cgi?rs=110767141) | 0.46301 | 3.36E-05 | ns | ns | s5 | ns |
| BovineHD0500015138 | 5 | 52777150 | [rs109572573](http://www.ncbi.nlm.nih.gov/projects/SNP/snp_ref.cgi?rs=109572573) | 0.537275 | 2.65E-05 | ns | ns | s5 | ns |
| BovineHD0500015140 | 5 | 52797774 | [rs110762622](http://www.ncbi.nlm.nih.gov/projects/SNP/snp_ref.cgi?rs=110762622) | 0.46301 | 3.36E-05 | ns | ns | s5 | ns |
| BovineHD0500015328 | 5 | 53730923 | [rs137139553](http://www.ncbi.nlm.nih.gov/projects/SNP/snp_ref.cgi?rs=137139553) | 0.676471 | 2.69E-04 | ns | ns | s10 | ns |
| BovineHD0500015332 | 5 | 53739452 | [rs135522177](http://www.ncbi.nlm.nih.gov/projects/SNP/snp_ref.cgi?rs=135522177) | 0.32398 | 2.71E-04 | ns | ns | s10 | ns |
| BovineHD0500015335 | 5 | 53796209 | [rs109062985](http://www.ncbi.nlm.nih.gov/projects/SNP/snp_ref.cgi?rs=109062985) | 0.322251 | 2.56E-04 | ns | ns | s10 | ns |
| BovineHD0500015336 | 5 | 53797927 | [rs110673933](http://www.ncbi.nlm.nih.gov/projects/SNP/snp_ref.cgi?rs=110673933) | 0.677296 | 2.43E-04 | ns | ns | s10 | ns |
| BovineHD0500015337 | 5 | 53806708 | [rs133769998](http://www.ncbi.nlm.nih.gov/projects/SNP/snp_ref.cgi?rs=133769998) | 0.677296 | 2.43E-04 | ns | ns | s10 | ns |
| BovineHD0500015416 | 5 | 54123443 | [rs134919998](http://www.ncbi.nlm.nih.gov/projects/SNP/snp_ref.cgi?rs=134919998) | 0.732143 | 8.52E-05 | ns | ns | s5 | ns |
| BovineHD0500015417 | 5 | 54126389 | [rs109455016](http://www.ncbi.nlm.nih.gov/projects/SNP/snp_ref.cgi?rs=109455016) | 0.246787 | 4.18E-04 | ns | ns | s10 | ns |
| BovineHD0500015468 | 5 | 54305408 | [rs110845453](http://www.ncbi.nlm.nih.gov/projects/SNP/snp_ref.cgi?rs=110845453) | 0.189258 | 3.97E-04 | ns | ns | s10 | ns |
| ARS-BFGL-NGS-30772 | 5 | 54613068 | [rs110311658](http://www.ncbi.nlm.nih.gov/projects/SNP/snp_ref.cgi?rs=110311658) | 0.287724 | 3.98E-04 | ns | ns | s10 | ns |
| BovineHD0500015616 | 5 | 54743792 | [rs109035507](http://www.ncbi.nlm.nih.gov/projects/SNP/snp_ref.cgi?rs=109035507) | 0.348214 | 2.03E-04 | ns | ns | s10 | ns |
| BovineHD0500015619 | 5 | 54751685 | [rs135645960](http://www.ncbi.nlm.nih.gov/projects/SNP/snp_ref.cgi?rs=135645960) | 0.644133 | 3.18E-04 | ns | ns | s10 | ns |
| BovineHD0500015621 | 5 | 54754759 | [rs133048773](http://www.ncbi.nlm.nih.gov/projects/SNP/snp_ref.cgi?rs=133048773) | 0.359335 | 3.57E-04 | ns | ns | s10 | ns |
| BovineHD0500015622 | 5 | 54756688 | [rs137246199](http://www.ncbi.nlm.nih.gov/projects/SNP/snp_ref.cgi?rs=137246199) | 0.624031 | 1.13E-04 | ns | ns | s5 | ns |
| BovineHD0500015623 | 5 | 54760249 | [rs136147741](http://www.ncbi.nlm.nih.gov/projects/SNP/snp_ref.cgi?rs=136147741) | 0.584399 | 3.64E-04 | ns | ns | s10 | ns |
| BovineHD0500018169 | 5 | 64946171 | [rs132893231](http://www.ncbi.nlm.nih.gov/projects/SNP/snp_ref.cgi?rs=132893231) | 0.202806 | 1.29E-04 | ns | ns | s10 | ns |
| BovineHD0500019587 | 5 | 69947321 | [rs134969556](http://www.ncbi.nlm.nih.gov/projects/SNP/snp_ref.cgi?rs=134969556) | 0.124359 | 1.81E-04 | ns | ns | s10 | ns |
| BovineHD0500024388 | 5 | 86091862 | [rs43445896](http://www.ncbi.nlm.nih.gov/projects/SNP/snp_ref.cgi?rs=43445896) | 0.675192 | 1.77E-04 | ns | ns | s10 | ns |
| BovineHD0500027311 | 5 | 96184216 | [rs109408384](http://www.ncbi.nlm.nih.gov/projects/SNP/snp_ref.cgi?rs=109408384) | 0.936224 | 6.79E-05 | ns | ns | s5 | ns |
| BovineHD0500028013 | 5 | 98265736 | [rs136476913](http://www.ncbi.nlm.nih.gov/projects/SNP/snp_ref.cgi?rs=136476913) | 0.777494 | 2.78E-04 | ns | ns | s10 | ns |
| BovineHD0500028848 | 5 | 100742852 | [rs110460517](http://www.ncbi.nlm.nih.gov/projects/SNP/snp_ref.cgi?rs=110460517) | 0.225806 | 1.13E-04 | ns | ns | s5 | ns |
| BovineHD0500036252 | 5 | 107391947 | [rs135705262](http://www.ncbi.nlm.nih.gov/projects/SNP/snp_ref.cgi?rs=135705262) | 0.580357 | 4.00E-04 | ns | ns | s10 | ns |
| BovineHD0900003930 | 9 | 15010015 | [rs43582369](http://www.ncbi.nlm.nih.gov/projects/SNP/snp_ref.cgi?rs=43582369) | 0.631378 | 8.11E-06 | ns | ns | s5 | s10 |
| BovineHD0900004240 | 9 | 15877491 | [rs110130556](http://www.ncbi.nlm.nih.gov/projects/SNP/snp_ref.cgi?rs=110130556) | 0.639386 | 9.07E-07 | s5 | ns | s5 | s5 |
| BovineHD0900004242 | 9 | 15880619 | [rs43582288](http://www.ncbi.nlm.nih.gov/projects/SNP/snp_ref.cgi?rs=43582288) | 0.631378 | 6.66E-07 | s5 | ns | s5 | s5 |
| BovineHD0900004243 | 9 | 15881203 | [rs43582290](http://www.ncbi.nlm.nih.gov/projects/SNP/snp_ref.cgi?rs=43582290) | 0.363171 | 1.72E-06 | s5 | ns | s5 | s5 |
| BovineHD0900005569 | 9 | 20508034 | [rs43587922](http://www.ncbi.nlm.nih.gov/projects/SNP/snp_ref.cgi?rs=43587922) | 0.0739796 | 4.26E-06 | ns | ns | s5 | s10 |
| BovineHD0900005571 | 9 | 20510296 | [rs110633796](http://www.ncbi.nlm.nih.gov/projects/SNP/snp_ref.cgi?rs=110633796) | 0.0739796 | 4.26E-06 | ns | ns | s5 | s10 |
| BovineHD0900007743 | 9 | 28601347 | [rs42242832](http://www.ncbi.nlm.nih.gov/projects/SNP/snp_ref.cgi?rs=42242832) | 0.938776 | 2.58E-05 | ns | ns | s10 | ns |
| BovineHD0900026520 | 9 | 93702202 | [rs137387359](http://www.ncbi.nlm.nih.gov/projects/SNP/snp_ref.cgi?rs=137387359) | 0.563776 | 1.78E-05 | ns | ns | s10 | ns |
| BovineHD0900026522 | 9 | 93704329 | [rs137153440](http://www.ncbi.nlm.nih.gov/projects/SNP/snp_ref.cgi?rs=137153440) | 0.556995 | 2.94E-05 | ns | ns | s10 | ns |
| BovineHD0900026523 | 9 | 93705281 | [rs135164150](http://www.ncbi.nlm.nih.gov/projects/SNP/snp_ref.cgi?rs=135164150) | 0.689744 | 2.94E-06 | s10 | ns | s5 | s5 |
| BovineHD0900026524 | 9 | 93708507 | [rs132757037](http://www.ncbi.nlm.nih.gov/projects/SNP/snp_ref.cgi?rs=132757037) | 0.4375 | 1.99E-05 | ns | ns | s10 | ns |
| BovineHD1300019853 | 13 | 69505036 | [rs134336444](http://www.ncbi.nlm.nih.gov/projects/SNP/snp_ref.cgi?rs=134336444) | 0.644133 | 5.89E-06 | ns | ns | s5 | s10 |
| BovineHD1300020104 | 13 | 70387545 | [rs134822694](http://www.ncbi.nlm.nih.gov/projects/SNP/snp_ref.cgi?rs=134822694) | 0.798469 | 3.86E-06 | s10 | ns | s5 | s10 |
| BovineHD1300020107 | 13 | 70398426 | [rs135287766](http://www.ncbi.nlm.nih.gov/projects/SNP/snp_ref.cgi?rs=135287766) | 0.798469 | 3.86E-06 | s10 | ns | s5 | s10 |
| BovineHD1300020124 | 13 | 70485807 | [rs133273718](http://www.ncbi.nlm.nih.gov/projects/SNP/snp_ref.cgi?rs=133273718) | 0.769133 | 2.11E-05 | ns | ns | s10 | ns |
| BovineHD1300020278 | 13 | 70929605 | [rs137564189](http://www.ncbi.nlm.nih.gov/projects/SNP/snp_ref.cgi?rs=137564189) | 0.498724 | 2.35E-05 | ns | ns | s10 | ns |
| Hapmap30381-BTC-005750 | 14 | 1463676 | [rs110090404](http://www.ncbi.nlm.nih.gov/projects/SNP/snp_ref.cgi?rs=110090404) | 0.733418 | 1.02E-03 | ns | ns | s10 | ns |
| BovineHD1400002448 | 14 | 8863266 | [rs133748371](http://www.ncbi.nlm.nih.gov/projects/SNP/snp_ref.cgi?rs=133748371) | 0.950255 | 3.46E-05 | ns | ns | s5 | ns |
| BovineHD1400002457 | 14 | 8907569 | [rs109480281](http://www.ncbi.nlm.nih.gov/projects/SNP/snp_ref.cgi?rs=109480281) | 0.905612 | 6.53E-04 | ns | ns | s10 | ns |
| BovineHD1400003189 | 14 | 11330635 | [rs110751858](http://www.ncbi.nlm.nih.gov/projects/SNP/snp_ref.cgi?rs=110751858) | 0.696429 | 1.55E-04 | ns | ns | s5 | ns |
| BovineHD1400005283 | 14 | 18417472 | [rs133961677](http://www.ncbi.nlm.nih.gov/projects/SNP/snp_ref.cgi?rs=133961677) | 0.493622 | 1.06E-03 | ns | ns | s10 | ns |
| BovineHD1400005300 | 14 | 18464165 | [s109618600](http://www.ncbi.nlm.nih.gov/projects/SNP/snp_ref.cgi?rs=109618600) | 0.448849 | 4.03E-04 | ns | ns | s10 | ns |
| BovineHD1400005301 | 14 | 18467034 | [rs111000599](http://www.ncbi.nlm.nih.gov/projects/SNP/snp_ref.cgi?rs=111000599) | 0.521684 | 1.79E-04 | ns | ns | s5 | ns |
| BovineHD1400008918 | 14 | 30872791 | [rs134424688](http://www.ncbi.nlm.nih.gov/projects/SNP/snp_ref.cgi?rs=134424688) | 0.678571 | 9.25E-04 | ns | ns | s10 | ns |
| BovineHD1400008980 | 14 | 31103722 | [rs133066519](http://www.ncbi.nlm.nih.gov/projects/SNP/snp_ref.cgi?rs=133066519) | 0.658163 | 8.37E-04 | ns | ns | s10 | ns |
| BovineHD1400010029 | 14 | 34762775 | [rs132764742](http://www.ncbi.nlm.nih.gov/projects/SNP/snp_ref.cgi?rs=132764742) | 0.269821 | 1.12E-03 | ns | ns | s10 | ns |
| BovineHD1400010491 | 14 | 36445956 | [rs135075316](http://www.ncbi.nlm.nih.gov/projects/SNP/snp_ref.cgi?rs=135075316) | 0.860614 | 2.66E-04 | ns | ns | s5 | ns |
| BovineHD1400010538 | 14 | 36694209 | [rs136664344](http://www.ncbi.nlm.nih.gov/projects/SNP/snp_ref.cgi?rs=136664344) | 0.858974 | 1.49E-04 | ns | ns | s5 | ns |
| BovineHD1400010572 | 14 | 36825730 | [rs134745604](http://www.ncbi.nlm.nih.gov/projects/SNP/snp_ref.cgi?rs=134745604) | 0.758108 | 1.07E-04 | ns | ns | s5 | ns |
| BovineHD1400010573 | 14 | 36827753 | [rs137743952](http://www.ncbi.nlm.nih.gov/projects/SNP/snp_ref.cgi?rs=137743952) | 0.755102 | 3.31E-04 | ns | ns | s5 | ns |
| BovineHD1400010580 | 14 | 36847073 | [rs134361509](http://www.ncbi.nlm.nih.gov/projects/SNP/snp_ref.cgi?rs=134361509) | 0.826531 | 1.04E-03 | ns | ns | s10 | ns |
| BovineHD1400010582 | 14 | 36853296 | [rs136612113](http://www.ncbi.nlm.nih.gov/projects/SNP/snp_ref.cgi?rs=136612113) | 0.289541 | 1.13E-03 | ns | ns | s10 | ns |
| BovineHD1400010583 | 14 | 36855513 | [rs134103447](http://www.ncbi.nlm.nih.gov/projects/SNP/snp_ref.cgi?rs=134103447) | 0.309463 | 2.52E-04 | ns | ns | s5 | ns |
| BovineHD1400010584 | 14 | 36859907 | [rs137681879](http://www.ncbi.nlm.nih.gov/projects/SNP/snp_ref.cgi?rs=137681879) | 0.308673 | 3.79E-04 | ns | ns | s10 | ns |
| BovineHD1400010698 | 14 | 37341625 | [rs110656764](http://www.ncbi.nlm.nih.gov/projects/SNP/snp_ref.cgi?rs=110656764) | 0.431122 | 9.45E-04 | ns | ns | s10 | ns |
| BovineHD1400010707 | 14 | 37386478 | [rs133726980](http://www.ncbi.nlm.nih.gov/projects/SNP/snp_ref.cgi?rs=133726980) | 0.303571 | 9.91E-04 | ns | ns | s10 | ns |
| BovineHD1400010722 | 14 | 37468074 | [rs137187252](http://www.ncbi.nlm.nih.gov/projects/SNP/snp_ref.cgi?rs=137187252) | 0.129738 | 1.15E-03 | ns | ns | s10 | ns |
| BovineHD1400010733 | 14 | 37508921 | [rs134037510](http://www.ncbi.nlm.nih.gov/projects/SNP/snp_ref.cgi?rs=134037510) | 0.260204 | 1.14E-03 | ns | ns | s10 | ns |
| BovineHD1400010734 | 14 | 37511658 | [rs135657472](http://www.ncbi.nlm.nih.gov/projects/SNP/snp_ref.cgi?rs=135657472) | 0.260204 | 1.14E-03 | ns | ns | s10 | ns |
| BovineHD1400010739 | 14 | 37528030 | [rs134109294](http://www.ncbi.nlm.nih.gov/projects/SNP/snp_ref.cgi?rs=134109294) | 0.265306 | 6.09E-04 | ns | ns | s10 | ns |
| BovineHD1400010743 | 14 | 37546712 | [rs135448359](http://www.ncbi.nlm.nih.gov/projects/SNP/snp_ref.cgi?rs=135448359) | 0.25 | 7.47E-04 | ns | ns | s10 | ns |
| BovineHD1400010745 | 14 | 37552403 | [rs133171963](http://www.ncbi.nlm.nih.gov/projects/SNP/snp_ref.cgi?rs=133171963) | 0.25 | 7.47E-04 | ns | ns | s10 | ns |
| BovineHD1400010753 | 14 | 37598625 | [rs135238827](http://www.ncbi.nlm.nih.gov/projects/SNP/snp_ref.cgi?rs=135238827) | 0.653061 | 1.20E-03 | ns | ns | s10 | ns |
| BovineHD1400010826 | 14 | 37856080 | [rs132804124](http://www.ncbi.nlm.nih.gov/projects/SNP/snp_ref.cgi?rs=132804124) | 0.798469 | 5.73E-05 | ns | ns | s5 | ns |
| BovineHD1400010827 | 14 | 37859817 | [rs137073386](http://www.ncbi.nlm.nih.gov/projects/SNP/snp_ref.cgi?rs=137073386) | 0.839489 | 1.57E-05 | ns | ns | s5 | ns |
| BovineHD1400010828 | 14 | 37862175 | [rs135938474](http://www.ncbi.nlm.nih.gov/projects/SNP/snp_ref.cgi?rs=135938474) | 0.806122 | 6.99E-05 | ns | ns | s5 | ns |
| BovineHD1400010852 | 14 | 37945078 | [rs137070738](http://www.ncbi.nlm.nih.gov/projects/SNP/snp_ref.cgi?rs=137070738) | 0.209184 | 6.04E-05 | ns | ns | s5 | ns |
| BovineHD1400011155 | 14 | 38889349 | [rs137465376](http://www.ncbi.nlm.nih.gov/projects/SNP/snp_ref.cgi?rs=137465376) | 0.795918 | 8.66E-05 | ns | ns | s5 | ns |
| BovineHD1400011162 | 14 | 38913780 | [rs134711539](http://www.ncbi.nlm.nih.gov/projects/SNP/snp_ref.cgi?rs=134711539) | 0.836364 | 2.33E-04 | ns | ns | s5 | ns |
| BovineHD1400011176 | 14 | 38969389 | [rs137821036](http://www.ncbi.nlm.nih.gov/projects/SNP/snp_ref.cgi?rs=137821036) | 0.102041 | 5.00E-04 | ns | ns | s10 | ns |
| BovineHD1400011451 | 14 | 39912946 | [rs109995322](http://www.ncbi.nlm.nih.gov/projects/SNP/snp_ref.cgi?rs=109995322) | 0.306905 | 1.10E-03 | ns | ns | s10 | ns |
| BovineHD1400011955 | 14 | 41979867 | [rs134984970](http://www.ncbi.nlm.nih.gov/projects/SNP/snp_ref.cgi?rs=134984970) | 0.877551 | 7.23E-04 | ns | ns | s10 | ns |
| BovineHD1400011956 | 14 | 41985381 | [rs42142739](http://www.ncbi.nlm.nih.gov/projects/SNP/snp_ref.cgi?rs=42142739) | 0.352041 | 1.19E-03 | ns | ns | s10 | ns |
| BovineHD1400011958 | 14 | 41993797 | [rs42142629](http://www.ncbi.nlm.nih.gov/projects/SNP/snp_ref.cgi?rs=42142629) | 0.330357 | 7.55E-04 | ns | ns | s10 | ns |
| BovineHD1400011972 | 14 | 42051798 | [rs134584620](http://www.ncbi.nlm.nih.gov/projects/SNP/snp_ref.cgi?rs=134584620) | 0.873724 | 2.91E-04 | ns | ns | s5 | ns |
| BovineHD1400012016 | 14 | 42210135 | [rs134053575](http://www.ncbi.nlm.nih.gov/projects/SNP/snp_ref.cgi?rs=134053575) | 0.247449 | 7.34E-04 | ns | ns | s10 | ns |
| BovineHD1400012053 | 14 | 42330263 | [rs137442228](http://www.ncbi.nlm.nih.gov/projects/SNP/snp_ref.cgi?rs=137442228) | 0.790816 | 8.39E-04 | ns | ns | s10 | ns |
| Hapmap32993-BTA-129125 | 14 | 42363191 | [rs110035827](http://www.ncbi.nlm.nih.gov/projects/SNP/snp_ref.cgi?rs=110035827) | 0.789541 | 4.10E-04 | ns | ns | s10 | ns |
| BovineHD1400012065 | 14 | 42371165 | [s41730291](http://www.ncbi.nlm.nih.gov/projects/SNP/snp_ref.cgi?rs=41730291) | 0.646684 | 9.80E-04 | ns | ns | s10 | ns |
| BovineHD1400012185 | 14 | 42858774 | [rs137101443](http://www.ncbi.nlm.nih.gov/projects/SNP/snp_ref.cgi?rs=137101443) | 0.262755 | 4.16E-04 | ns | ns | s10 | ns |
| BovineHD1400012235 | 14 | 43086421 | [rs136174931](http://www.ncbi.nlm.nih.gov/projects/SNP/snp_ref.cgi?rs=136174931) | 0.825255 | 8.97E-04 | ns | ns | s10 | ns |
| BovineHD1400012236 | 14 | 43088007 | [rs42365139](http://www.ncbi.nlm.nih.gov/projects/SNP/snp_ref.cgi?rs=42365139) | 0.756378 | 3.49E-04 | ns | ns | s10 | ns |
| BovineHD1400012240 | 14 | 43104229 | [rs137216648](http://www.ncbi.nlm.nih.gov/projects/SNP/snp_ref.cgi?rs=137216648) | 0.728316 | 2.14E-04 | ns | ns | s5 | ns |
| BovineHD1400012241 | 14 | 43107811 | [rs42856291](http://www.ncbi.nlm.nih.gov/projects/SNP/snp_ref.cgi?rs=42856291) | 0.184143 | 2.56E-04 | ns | ns | s5 | ns |
| BovineHD1400012242 | 14 | 43111886 | [rs42856280](http://www.ncbi.nlm.nih.gov/projects/SNP/snp_ref.cgi?rs=42856280) | 0.307398 | 3.56E-04 | ns | ns | s10 | ns |
| BovineHD1400012243 | 14 | 43117014 | [rs42856277](http://www.ncbi.nlm.nih.gov/projects/SNP/snp_ref.cgi?rs=42856277) | 0.307398 | 3.56E-04 | ns | ns | s10 | ns |
| BovineHD1400012245 | 14 | 43124052 | [rs133558145](http://www.ncbi.nlm.nih.gov/projects/SNP/snp_ref.cgi?rs=133558145) | 0.785714 | 2.43E-04 | ns | ns | s5 | ns |
| BovineHD1400012250 | 14 | 43144741 | [rs137391994](http://www.ncbi.nlm.nih.gov/projects/SNP/snp_ref.cgi?rs=137391994) | 0.274936 | 1.12E-03 | ns | ns | s10 | ns |
| BovineHD1400012269 | 14 | 43301940 | [rs135129301](http://www.ncbi.nlm.nih.gov/projects/SNP/snp_ref.cgi?rs=135129301) | 0.821429 | 6.04E-04 | ns | ns | s10 | ns |
| BovineHD1400012296 | 14 | 43412378 | [rs136589835](http://www.ncbi.nlm.nih.gov/projects/SNP/snp_ref.cgi?rs=136589835) | 0.233516 | 1.01E-03 | ns | ns | s10 | ns |
| BovineHD1400012302 | 14 | 43442658 | [rs42856277](http://www.ncbi.nlm.nih.gov/projects/SNP/snp_ref.cgi?rs=42856277) | 0.607417 | 5.52E-04 | ns | ns | s10 | ns |
| BovineHD1400012334 | 14 | 43558498 | [rs133394363](http://www.ncbi.nlm.nih.gov/projects/SNP/snp_ref.cgi?rs=133394363) | 0.719072 | 5.30E-04 | ns | ns | s10 | ns |
| BovineHD1400012405 | 14 | 43821466 | [rs133748595](http://www.ncbi.nlm.nih.gov/projects/SNP/snp_ref.cgi?rs=133748595) | 0.929847 | 3.63E-04 | ns | ns | s10 | ns |
| BovineHD1400012412 | 14 | 43866070 | [rs137048645](http://www.ncbi.nlm.nih.gov/projects/SNP/snp_ref.cgi?rs=137048645) | 0.209184 | 1.04E-03 | ns | ns | s10 | ns |
| BovineHD1400012413 | 14 | 43872068 | [rs135901170](http://www.ncbi.nlm.nih.gov/projects/SNP/snp_ref.cgi?rs=135901170) | 0.876276 | 8.11E-04 | ns | ns | s10 | ns |
| BovineHD1400012414 | 14 | 43874123 | [rs136656767](http://www.ncbi.nlm.nih.gov/projects/SNP/snp_ref.cgi?rs=136656767) | 0.123724 | 8.11E-04 | ns | ns | s10 | ns |
| BovineHD1400012500 | 14 | 44164645 | [rs135173508](http://www.ncbi.nlm.nih.gov/projects/SNP/snp_ref.cgi?rs=135173508) | 0.798469 | 7.73E-04 | ns | ns | s10 | ns |
| BovineHD1400012534 | 14 | 44274052 | [rs135983396](http://www.ncbi.nlm.nih.gov/projects/SNP/snp_ref.cgi?rs=135983396) | 0.719388 | 6.39E-04 | ns | ns | s10 | ns |
| BovineHD1400012541 | 14 | 44304976 | [rs136887798](http://www.ncbi.nlm.nih.gov/projects/SNP/snp_ref.cgi?rs=136887798) | 0.280612 | 6.39E-04 | ns | ns | s10 | ns |
| BovineHD1400012542 | 14 | 44308751 | [rs135984324](http://www.ncbi.nlm.nih.gov/projects/SNP/snp_ref.cgi?rs=135984324) | 0.720513 | 7.07E-04 | ns | ns | s10 | ns |
| BovineHD1400012546 | 14 | 44324462 | [rs136708459](http://www.ncbi.nlm.nih.gov/projects/SNP/snp_ref.cgi?rs=136708459) | 0.280612 | 6.39E-04 | ns | ns | s10 | ns |
| BovineHD1400012552 | 14 | 44361488 | [rs133383682](http://www.ncbi.nlm.nih.gov/projects/SNP/snp_ref.cgi?rs=133383682) | 0.720663 | 1.05E-03 | ns | ns | s10 | ns |
| BovineHD1400012570 | 14 | 44462427 | [rs132630954](http://www.ncbi.nlm.nih.gov/projects/SNP/snp_ref.cgi?rs=132630954) | 0.728205 | 9.43E-04 | ns | ns | s10 | ns |
| BovineHD1400012623 | 14 | 44684721 | [rs133243977](http://www.ncbi.nlm.nih.gov/projects/SNP/snp_ref.cgi?rs=133243977) | 0.233418 | 3.87E-04 | ns | ns | s10 | ns |
| BovineHD1400012628 | 14 | 44699949 | [rs137737620](http://www.ncbi.nlm.nih.gov/projects/SNP/snp_ref.cgi?rs=137737620) | 0.270408 | 1.07E-03 | ns | ns | s10 | ns |
| BovineHD1400012766 | 14 | 45122378 | [rs132803686](http://www.ncbi.nlm.nih.gov/projects/SNP/snp_ref.cgi?rs=132803686) | 0.717391 | 2.95E-04 | ns | ns | s5 | ns |
| BovineHD1400012768 | 14 | 45127916 | [rs136891270](http://www.ncbi.nlm.nih.gov/projects/SNP/snp_ref.cgi?rs=136891270) | 0.281888 | 2.83E-04 | ns | ns | s5 | ns |
| BovineHD1400012779 | 14 | 45159242 | [rs134354120](http://www.ncbi.nlm.nih.gov/projects/SNP/snp_ref.cgi?rs=134354120) | 0.718112 | 2.83E-04 | ns | ns | s5 | ns |
| BovineHD1400012808 | 14 | 45269368 | [rs133901332](http://www.ncbi.nlm.nih.gov/projects/SNP/snp_ref.cgi?rs=133901332) | 0.285714 | 1.45E-04 | ns | ns | s5 | ns |
| BovineHD1400012811 | 14 | 45291493 | [rs134064937](http://www.ncbi.nlm.nih.gov/projects/SNP/snp_ref.cgi?rs=134064937) | 0.714286 | 1.45E-04 | ns | ns | s5 | ns |
| BovineHD1400012815 | 14 | 45304557 | [rs135297170](http://www.ncbi.nlm.nih.gov/projects/SNP/snp_ref.cgi?rs=135297170) | 0.304847 | 9.26E-05 | ns | ns | s5 | ns |
| BovineHD1400012816 | 14 | 45306542 | [rs136659909](http://www.ncbi.nlm.nih.gov/projects/SNP/snp_ref.cgi?rs=136659909) | 0.159847 | 1.44E-05 | ns | ns | s5 | ns |
| BovineHD1400012817 | 14 | 45308555 | [rs134211847](http://www.ncbi.nlm.nih.gov/projects/SNP/snp_ref.cgi?rs=134211847) | 0.695153 | 9.26E-05 | ns | ns | s5 | ns |
| BovineHD1400012818 | 14 | 45312933 | [rs137570609](http://www.ncbi.nlm.nih.gov/projects/SNP/snp_ref.cgi?rs=137570609) | 0.695153 | 9.26E-05 | ns | ns | s5 | ns |
| BovineHD1400012822 | 14 | 45330616 | [rs133401075](http://www.ncbi.nlm.nih.gov/projects/SNP/snp_ref.cgi?rs=133401075) | 0.130102 | 1.17E-04 | ns | ns | s5 | ns |
| BovineHD1400012823 | 14 | 45333851 | [rs110113985](http://www.ncbi.nlm.nih.gov/projects/SNP/snp_ref.cgi?rs=110113985) | 0.109694 | 7.96E-04 | ns | ns | s10 | ns |
| BovineHD1400012830 | 14 | 45358998 | [rs110112317](http://www.ncbi.nlm.nih.gov/projects/SNP/snp_ref.cgi?rs=110112317) | 0.782609 | 7.30E-04 | ns | ns | s10 | ns |
| BovineHD1400012831 | 14 | 45363612 | [rs133715055](http://www.ncbi.nlm.nih.gov/projects/SNP/snp_ref.cgi?rs=133715055) | 0.638107 | 6.56E-06 | ns | ns | s5 | s10 |
| BovineHD1400012832 | 14 | 45366221 | [rs109442520](http://www.ncbi.nlm.nih.gov/projects/SNP/snp_ref.cgi?rs=109442520) | 0.635204 | 1.02E-06 | s5 | ns | s5 | s5 |
| BovineHD1400012836 | 14 | 45375492 | [rs136608009](http://www.ncbi.nlm.nih.gov/projects/SNP/snp_ref.cgi?rs=136608009) | 0.361893 | 1.19E-06 | s5 | ns | s5 | s5 |
| BovineHD1400012838 | 14 | 45383712 | [rs136372877](http://www.ncbi.nlm.nih.gov/projects/SNP/snp_ref.cgi?rs=136372877) | 0.210452 | 2.50E-05 | ns | ns | s5 | ns |
| BovineHD1400012839 | 14 | 45387715 | [rs110643403](http://www.ncbi.nlm.nih.gov/projects/SNP/snp_ref.cgi?rs=110643403) | 0.390026 | 1.09E-05 | ns | ns | s5 | ns |
| BovineHD1400012840 | 14 | 45391498 | [rs134638942](http://www.ncbi.nlm.nih.gov/projects/SNP/snp_ref.cgi?rs=134638942) | 0.771684 | 7.61E-04 | ns | ns | s10 | ns |
| BovineHD1400012841 | 14 | 45395111 | [rs137572973](http://www.ncbi.nlm.nih.gov/projects/SNP/snp_ref.cgi?rs=137572973) | 0.278646 | 6.94E-05 | ns | ns | s5 | ns |
| BovineHD1400012842 | 14 | 45399460 | [rs133174611](http://www.ncbi.nlm.nih.gov/projects/SNP/snp_ref.cgi?rs=133174611) | 0.718112 | 6.71E-05 | ns | ns | s5 | ns |
| BovineHD1400012844 | 14 | 45406866 | [rs137339424](http://www.ncbi.nlm.nih.gov/projects/SNP/snp_ref.cgi?rs=137339424) | 0.608418 | 1.09E-03 | ns | ns | s10 | ns |
| BovineHD1400012845 | 14 | 45410649 | [rs135782413](http://www.ncbi.nlm.nih.gov/projects/SNP/snp_ref.cgi?rs=135782413) | 0.145408 | 1.26E-05 | ns | ns | s5 | ns |
| BovineHD1400012847 | 14 | 45417177 | [rs134521313](http://www.ncbi.nlm.nih.gov/projects/SNP/snp_ref.cgi?rs=134521313) | 0.150895 | 3.46E-04 | ns | ns | s10 | ns |
| BovineHD1400012848 | 14 | 45418904 | [rs136159423](http://www.ncbi.nlm.nih.gov/projects/SNP/snp_ref.cgi?rs=136159423) | 0.151786 | 4.14E-04 | ns | ns | s10 | ns |
| BovineHD1400012849 | 14 | 45423941 | [rs133881536](http://www.ncbi.nlm.nih.gov/projects/SNP/snp_ref.cgi?rs=133881536) | 0.337596 | 3.14E-04 | ns | ns | s5 | ns |
| BovineHD1400012850 | 14 | 45427353 | [rs134722896](http://www.ncbi.nlm.nih.gov/projects/SNP/snp_ref.cgi?rs=134722896) | 0.337596 | 3.14E-04 | ns | ns | s5 | ns |
| BovineHD1400012851 | 14 | 45430253 | [rs137744254](http://www.ncbi.nlm.nih.gov/projects/SNP/snp_ref.cgi?rs=137744254) | 0.283163 | 7.14E-05 | ns | ns | s5 | ns |
| BovineHD1400012853 | 14 | 45435372 | [rs137510178](http://www.ncbi.nlm.nih.gov/projects/SNP/snp_ref.cgi?rs=137510178) | 0.280612 | 8.01E-05 | ns | ns | s5 | ns |
| BovineHD1400012854 | 14 | 45439004 | [rs133002930](http://www.ncbi.nlm.nih.gov/projects/SNP/snp_ref.cgi?rs=133002930) | 0.719388 | 8.01E-05 | ns | ns | s5 | ns |
| BovineHD1400012855 | 14 | 45441865 | [rs134041698](http://www.ncbi.nlm.nih.gov/projects/SNP/snp_ref.cgi?rs=134041698) | 0.797194 | 4.78E-04 | ns | ns | s10 | ns |
| BovineHD1400012856 | 14 | 45444327 | [rs135639560](http://www.ncbi.nlm.nih.gov/projects/SNP/snp_ref.cgi?rs=135639560) | 0.719388 | 8.01E-05 | ns | ns | s5 | ns |
| BovineHD1400012857 | 14 | 45451260 | [rs132639160](http://www.ncbi.nlm.nih.gov/projects/SNP/snp_ref.cgi?rs=132639160) | 0.264031 | 5.95E-04 | ns | ns | s10 | ns |
| BovineHD1400012859 | 14 | 45457772 | [rs135760404](http://www.ncbi.nlm.nih.gov/projects/SNP/snp_ref.cgi?rs=135760404) | 0.798469 | 4.46E-04 | ns | ns | s10 | ns |
| BovineHD1400012861 | 14 | 45462775 | [rs134062262](http://www.ncbi.nlm.nih.gov/projects/SNP/snp_ref.cgi?rs=134062262) | 0.716837 | 7.14E-05 | ns | ns | s5 | ns |
| BovineHD1400012862 | 14 | 45464854 | [rs137679118](http://www.ncbi.nlm.nih.gov/projects/SNP/snp_ref.cgi?rs=137679118) | 0.283163 | 7.14E-05 | ns | ns | s5 | ns |
| BovineHD1400012863 | 14 | 45466986 | [rs133399056](http://www.ncbi.nlm.nih.gov/projects/SNP/snp_ref.cgi?rs=133399056) | 0.716837 | 7.14E-05 | ns | ns | s5 | ns |
| BovineHD1400012866 | 14 | 45474794 | [rs133080568](http://www.ncbi.nlm.nih.gov/projects/SNP/snp_ref.cgi?rs=133080568) | 0.608418 | 1.09E-03 | ns | ns | s10 | ns |
| BovineHD1400012867 | 14 | 45478315 | [rs137341841](http://www.ncbi.nlm.nih.gov/projects/SNP/snp_ref.cgi?rs=137341841) | 0.332481 | 4.22E-04 | ns | ns | s10 | ns |
| BovineHD1400012869 | 14 | 45490190 | [rs136942595](http://www.ncbi.nlm.nih.gov/projects/SNP/snp_ref.cgi?rs=136942595) | 0.869898 | 1.17E-04 | ns | ns | s5 | ns |
| BovineHD1400012870 | 14 | 45495995 | [rs134553723](http://www.ncbi.nlm.nih.gov/projects/SNP/snp_ref.cgi?rs=134553723) | 0.367347 | 1.21E-04 | ns | ns | s5 | ns |
| BovineHD1400012871 | 14 | 45498366 | [rs136546448](http://www.ncbi.nlm.nih.gov/projects/SNP/snp_ref.cgi?rs=136546448) | 0.632653 | 1.21E-04 | ns | ns | s5 | ns |
| BovineHD1400012874 | 14 | 45506244 | [rs134233283](http://www.ncbi.nlm.nih.gov/projects/SNP/snp_ref.cgi?rs=134233283) | 0.373402 | 8.27E-05 | ns | ns | s5 | ns |
| BovineHD1400012876 | 14 | 45510891 | [rs136442964](http://www.ncbi.nlm.nih.gov/projects/SNP/snp_ref.cgi?rs=136442964) | 0.396419 | 1.19E-03 | ns | ns | s10 | ns |
| BovineHD1400012878 | 14 | 45519212 | [rs133649827](http://www.ncbi.nlm.nih.gov/projects/SNP/snp_ref.cgi?rs=133649827) | 0.372449 | 8.79E-05 | ns | ns | s5 | ns |
| BovineHD1400012879 | 14 | 45521484 | [rs137512750](http://www.ncbi.nlm.nih.gov/projects/SNP/snp_ref.cgi?rs=137512750) | 0.846939 | 4.18E-04 | ns | ns | s10 | ns |
| BovineHD1400012881 | 14 | 45525481 | [rs137113350](http://www.ncbi.nlm.nih.gov/projects/SNP/snp_ref.cgi?rs=137113350) | 0.794643 | 9.93E-05 | ns | ns | s5 | ns |
| BovineHD1400012882 | 14 | 45530596 | [rs135047163](http://www.ncbi.nlm.nih.gov/projects/SNP/snp_ref.cgi?rs=135047163) | 0.706633 | 2.46E-04 | ns | ns | s5 | ns |
| BovineHD1400012883 | 14 | 45534903 | [rs110246732](http://www.ncbi.nlm.nih.gov/projects/SNP/snp_ref.cgi?rs=110246732) | 0.608696 | 1.82E-05 | ns | ns | s5 | ns |
| BovineHD1400012886 | 14 | 45547871 | [rs110610723](http://www.ncbi.nlm.nih.gov/projects/SNP/snp_ref.cgi?rs=110610723) | 0.387468 | 2.15E-06 | s5 | ns | s5 | s5 |
| BovineHD1400012891 | 14 | 45570200 | [rs136434991](http://www.ncbi.nlm.nih.gov/projects/SNP/snp_ref.cgi?rs=136434991) | 0.80102 | 7.71E-04 | ns | ns | s10 | ns |
| BovineHD1400012898 | 14 | 45612132 | [rs137690798](http://www.ncbi.nlm.nih.gov/projects/SNP/snp_ref.cgi?rs=137690798) | 0.321429 | 2.25E-04 | ns | ns | s5 | ns |
| BovineHD1400012901 | 14 | 45621339 | [rs132812346](http://www.ncbi.nlm.nih.gov/projects/SNP/snp_ref.cgi?rs=132812346) | 0.285714 | 7.66E-05 | ns | ns | s5 | ns |
| BovineHD1400012903 | 14 | 45628864 | [rs135301640](http://www.ncbi.nlm.nih.gov/projects/SNP/snp_ref.cgi?rs=135301640) | 0.285714 | 7.66E-05 | ns | ns | s5 | ns |
| BovineHD1400012906 | 14 | 45640628 | [rs135609702](http://www.ncbi.nlm.nih.gov/projects/SNP/snp_ref.cgi?rs=135609702) | 0.285714 | 7.66E-05 | ns | ns | s5 | ns |
| BovineHD1400012907 | 14 | 45647802 | [rs136719533](http://www.ncbi.nlm.nih.gov/projects/SNP/snp_ref.cgi?rs=136719533) | 0.193878 | 6.46E-04 | ns | ns | s10 | ns |
| BovineHD1400012909 | 14 | 45655468 | [rs136324758](http://www.ncbi.nlm.nih.gov/projects/SNP/snp_ref.cgi?rs=136324758) | 0.77551 | 1.11E-03 | ns | ns | s10 | ns |
| BovineHD1400012913 | 14 | 45679733 | [rs133469839](http://www.ncbi.nlm.nih.gov/projects/SNP/snp_ref.cgi?rs=133469839) | 0.264031 | 1.22E-04 | ns | ns | s5 | ns |
| BovineHD1400012914 | 14 | 45685588 | [rs137287756](http://www.ncbi.nlm.nih.gov/projects/SNP/snp_ref.cgi?rs=137287756) | 0.732143 | 6.41E-05 | ns | ns | s5 | ns |
| BovineHD1400012919 | 14 | 45704225 | [rs133719470](http://www.ncbi.nlm.nih.gov/projects/SNP/snp_ref.cgi?rs=133719470) | 0.200255 | 4.17E-04 | ns | ns | s10 | ns |
| BovineHD1400012923 | 14 | 45728192 | [rs137458332](http://www.ncbi.nlm.nih.gov/projects/SNP/snp_ref.cgi?rs=137458332) | 0.307398 | 7.13E-05 | ns | ns | s5 | ns |
| BovineHD1400012924 | 14 | 45732686 | [rs132804447](http://www.ncbi.nlm.nih.gov/projects/SNP/snp_ref.cgi?rs=132804447) | 0.309949 | 8.84E-05 | ns | ns | s5 | ns |
| BovineHD1400012982 | 14 | 45967520 | [rs135426196](http://www.ncbi.nlm.nih.gov/projects/SNP/snp_ref.cgi?rs=135426196) | 0.38648 | 5.24E-04 | ns | ns | s10 | ns |
| BovineHD1400012983 | 14 | 45971745 | [rs136495820](http://www.ncbi.nlm.nih.gov/projects/SNP/snp_ref.cgi?rs=136495820) | 0.248724 | 6.52E-04 | ns | ns | s10 | ns |
| BovineHD1400012985 | 14 | 45980054 | [rs133781069](http://www.ncbi.nlm.nih.gov/projects/SNP/snp_ref.cgi?rs=133781069) | 0.258929 | 6.32E-04 | ns | ns | s10 | ns |
| BovineHD1400012986 | 14 | 45982039 | [rs137400994](http://www.ncbi.nlm.nih.gov/projects/SNP/snp_ref.cgi?rs=137400994) | 0.258929 | 6.32E-04 | ns | ns | s10 | ns |
| BovineHD1400012987 | 14 | 45987797 | [rs135967036](http://www.ncbi.nlm.nih.gov/projects/SNP/snp_ref.cgi?rs=135967036) | 0.741071 | 6.32E-04 | ns | ns | s10 | ns |
| BovineHD1400013027 | 14 | 46113167 | [rs137747048](http://www.ncbi.nlm.nih.gov/projects/SNP/snp_ref.cgi?rs=137747048) | 0.300512 | 4.83E-05 | ns | ns | s5 | ns |
| BovineHD1400013035 | 14 | 46156400 | [rs109754076](http://www.ncbi.nlm.nih.gov/projects/SNP/snp_ref.cgi?rs=109754076) | 0.455357 | 2.19E-04 | ns | ns | s5 | ns |
| BovineHD1400013036 | 14 | 46158688 | [rs136612230](http://www.ncbi.nlm.nih.gov/projects/SNP/snp_ref.cgi?rs=136612230) | 0.276215 | 6.27E-04 | ns | ns | s10 | ns |
| BovineHD1400013039 | 14 | 46175095 | [rs110667294](http://www.ncbi.nlm.nih.gov/projects/SNP/snp_ref.cgi?rs=110667294) | 0.577806 | 1.09E-03 | ns | ns | s10 | ns |
| BovineHD1400013040 | 14 | 46177807 | [rs134236615](http://www.ncbi.nlm.nih.gov/projects/SNP/snp_ref.cgi?rs=134236615) | 0.322704 | 4.63E-05 | ns | ns | s5 | ns |
| BovineHD1400013042 | 14 | 46183165 | [rs133086073](http://www.ncbi.nlm.nih.gov/projects/SNP/snp_ref.cgi?rs=133086073) | 0.330357 | 4.62E-05 | ns | ns | s5 | ns |
| BovineHD1400013048 | 14 | 46197108 | [rs134247697](http://www.ncbi.nlm.nih.gov/projects/SNP/snp_ref.cgi?rs=134247697) | 0.427296 | 9.80E-04 | ns | ns | s10 | ns |
| BovineHD1400013050 | 14 | 46202477 | [rs136445928](http://www.ncbi.nlm.nih.gov/projects/SNP/snp_ref.cgi?rs=136445928) | 0.873402 | 1.10E-03 | ns | ns | s10 | ns |
| ARS-BFGL-NGS-91939 | 14 | 46236763 | [rs109900017](http://www.ncbi.nlm.nih.gov/projects/SNP/snp_ref.cgi?rs=109900017) | 0.522959 | 5.29E-04 | ns | ns | s10 | ns |
| BovineHD1400013069 | 14 | 46244107 | [rs136496010](http://www.ncbi.nlm.nih.gov/projects/SNP/snp_ref.cgi?rs=136496010) | 0.310256 | 1.25E-04 | ns | ns | s5 | ns |
| BovineHD1400013070 | 14 | 46246619 | [rs134044871](http://www.ncbi.nlm.nih.gov/projects/SNP/snp_ref.cgi?rs=134044871) | 0.688776 | 1.44E-04 | ns | ns | s5 | ns |
| BovineHD1400013072 | 14 | 46255387 | [rs137695047](http://www.ncbi.nlm.nih.gov/projects/SNP/snp_ref.cgi?rs=137695047) | 0.34949 | 8.18E-04 | ns | ns | s10 | ns |
| BovineHD1400013073 | 14 | 46259505 | [rs110036121](http://www.ncbi.nlm.nih.gov/projects/SNP/snp_ref.cgi?rs=110036121) | 0.612245 | 1.75E-04 | ns | ns | s5 | ns |
| Hapmap29973-BTA-129162 | 14 | 46264806 | [rs109344263](http://www.ncbi.nlm.nih.gov/projects/SNP/snp_ref.cgi?rs=109344263) | 0.34949 | 8.18E-04 | ns | ns | s10 | ns |
| BovineHD1400013074 | 14 | 46268019 | [rs137459798](http://www.ncbi.nlm.nih.gov/projects/SNP/snp_ref.cgi?rs=137459798) | 0.311224 | 1.44E-04 | ns | ns | s5 | ns |
| BovineHD1400013077 | 14 | 46287009 | [rs135311524](http://www.ncbi.nlm.nih.gov/projects/SNP/snp_ref.cgi?rs=135311524) | 0.34949 | 8.18E-04 | ns | ns | s10 | ns |
| BovineHD1400013078 | 14 | 46289854 | [rs132944088](http://www.ncbi.nlm.nih.gov/projects/SNP/snp_ref.cgi?rs=132944088) | 0.309949 | 1.78E-04 | ns | ns | s5 | ns |
| BovineHD1400013079 | 14 | 46293135 | [rs137120971](http://www.ncbi.nlm.nih.gov/projects/SNP/snp_ref.cgi?rs=137120971) | 0.308673 | 2.33E-04 | ns | ns | s5 | ns |
| BovineHD1400013082 | 14 | 46311807 | [rs134407664](http://www.ncbi.nlm.nih.gov/projects/SNP/snp_ref.cgi?rs=134407664) | 0.177749 | 7.07E-04 | ns | ns | s10 | ns |
| BovineHD1400013083 | 14 | 46316147 | [rs137631985](http://www.ncbi.nlm.nih.gov/projects/SNP/snp_ref.cgi?rs=137631985) | 0.755102 | 2.63E-05 | ns | ns | s5 | ns |
| BovineHD1400013085 | 14 | 46321590 | [rs135035289](http://www.ncbi.nlm.nih.gov/projects/SNP/snp_ref.cgi?rs=135035289) | 0.76148 | 2.40E-04 | ns | ns | s5 | ns |
| BovineHD1400013086 | 14 | 46324091 | [rs136384647](http://www.ncbi.nlm.nih.gov/projects/SNP/snp_ref.cgi?rs=136384647) | 0.303571 | 1.42E-04 | ns | ns | s5 | ns |
| BovineHD1400013087 | 14 | 46329798 | [rs133634325](http://www.ncbi.nlm.nih.gov/projects/SNP/snp_ref.cgi?rs=133634325) | 0.303069 | 1.26E-04 | ns | ns | s5 | ns |
| BovineHD1400013088 | 14 | 46332532 | [rs137291182](http://www.ncbi.nlm.nih.gov/projects/SNP/snp_ref.cgi?rs=137291182) | 0.77551 | 1.64E-06 | s5 | ns | s5 | s5 |
| BovineHD1400013091 | 14 | 46340788 | [rs136894433](http://www.ncbi.nlm.nih.gov/projects/SNP/snp_ref.cgi?rs=136894433) | 0.299472 | 2.87E-04 | ns | ns | s5 | ns |
| BovineHD1400013092 | 14 | 46345442 | [rs134868727](http://www.ncbi.nlm.nih.gov/projects/SNP/snp_ref.cgi?rs=134868727) | 0.236573 | 3.66E-05 | ns | ns | s5 | ns |
| BovineHD1400013100 | 14 | 46366180 | [rs137462920](http://www.ncbi.nlm.nih.gov/projects/SNP/snp_ref.cgi?rs=137462920) | 0.30102 | 7.82E-05 | ns | ns | s5 | ns |
| BovineHD1400013103 | 14 | 46374694 | [rs137066271](http://www.ncbi.nlm.nih.gov/projects/SNP/snp_ref.cgi?rs=137066271) | 0.272494 | 1.29E-04 | ns | ns | s5 | ns |
| BovineHD1400013106 | 14 | 46384950 | [rs134665936](http://www.ncbi.nlm.nih.gov/projects/SNP/snp_ref.cgi?rs=134665936) | 0.637755 | 4.94E-06 | ns | ns | s5 | s10 |
| BovineHD1400013112 | 14 | 46402369 | [rs133332165](http://www.ncbi.nlm.nih.gov/projects/SNP/snp_ref.cgi?rs=133332165) | 0.420366 | 7.77E-04 | ns | ns | s10 | ns |
| BovineHD1400013114 | 14 | 46407572 | [rs135721856](http://www.ncbi.nlm.nih.gov/projects/SNP/snp_ref.cgi?rs=135721856) | 0.295918 | 5.66E-05 | ns | ns | s5 | ns |
| BovineHD1400013124 | 14 | 46432530 | [rs133794575](http://www.ncbi.nlm.nih.gov/projects/SNP/snp_ref.cgi?rs=133794575) | 0.558673 | 4.31E-05 | ns | ns | s5 | ns |
| BovineHD1400013125 | 14 | 46433863 | [rs135664659](http://www.ncbi.nlm.nih.gov/projects/SNP/snp_ref.cgi?rs=135664659) | 0.438776 | 2.88E-05 | ns | ns | s5 | ns |
| BovineHD1400013126 | 14 | 46436028 | [rs132652656](http://www.ncbi.nlm.nih.gov/projects/SNP/snp_ref.cgi?rs=132652656) | 0.22372 | 7.38E-04 | ns | ns | s10 | ns |
| BovineHD1400013128 | 14 | 46440530 | [rs135858520](http://www.ncbi.nlm.nih.gov/projects/SNP/snp_ref.cgi?rs=135858520) | 0.441026 | 3.08E-05 | ns | ns | s5 | ns |
| BovineHD1400013129 | 14 | 46441293 | [rs132821440](http://www.ncbi.nlm.nih.gov/projects/SNP/snp_ref.cgi?rs=132821440) | 0.561224 | 2.88E-05 | ns | ns | s5 | ns |
| BovineHD1400013130 | 14 | 46444557 | [rs137068943](http://www.ncbi.nlm.nih.gov/projects/SNP/snp_ref.cgi?rs=137068943) | 0.438776 | 2.88E-05 | ns | ns | s5 | ns |
| BovineHD1400013136 | 14 | 46460824 | [rs133091332](http://www.ncbi.nlm.nih.gov/projects/SNP/snp_ref.cgi?rs=133091332) | 0.556122 | 7.54E-05 | ns | ns | s5 | ns |
| BovineHD4100011510 | 14 | 46462511 | [rs41629829](http://www.ncbi.nlm.nih.gov/projects/SNP/snp_ref.cgi?rs=41629829) | 0.496173 | 3.28E-05 | ns | ns | s5 | ns |
| BovineHD1400013138 | 14 | 46463699 | [rs109185265](http://www.ncbi.nlm.nih.gov/projects/SNP/snp_ref.cgi?rs=109185265) | 0.605867 | 8.75E-04 | ns | ns | s10 | ns |
| ARS-BFGL-NGS-34212 | 14 | 46465704 | [rs110094547](http://www.ncbi.nlm.nih.gov/projects/SNP/snp_ref.cgi?rs=110094547) | 0.605867 | 8.75E-04 | ns | ns | s10 | ns |
| BovineHD1400013142 | 14 | 46473422 | [rs137006524](http://www.ncbi.nlm.nih.gov/projects/SNP/snp_ref.cgi?rs=137006524) | 0.503827 | 3.28E-05 | ns | ns | s5 | ns |
| BovineHD1400013151 | 14 | 46495330 | [rs136665695](http://www.ncbi.nlm.nih.gov/projects/SNP/snp_ref.cgi?rs=136665695) | 0.704082 | 5.66E-05 | ns | ns | s5 | ns |
| BovineHD1400013152 | 14 | 46501195 | [rs110270638](http://www.ncbi.nlm.nih.gov/projects/SNP/snp_ref.cgi?rs=110270638) | 0.516582 | 2.21E-04 | ns | ns | s5 | ns |
| BovineHD1400013155 | 14 | 46509108 | [rs134364376](http://www.ncbi.nlm.nih.gov/projects/SNP/snp_ref.cgi?rs=134364376) | 0.295918 | 5.66E-05 | ns | ns | s5 | ns |
| BovineHD1400013159 | 14 | 46521962 | [rs110881709](http://www.ncbi.nlm.nih.gov/projects/SNP/snp_ref.cgi?rs=110881709) | 0.548469 | 1.71E-05 | ns | ns | s5 | ns |
| BovineHD1400013162 | 14 | 46530797 | [rs134723944](http://www.ncbi.nlm.nih.gov/projects/SNP/snp_ref.cgi?rs=134723944) | 0.284211 | 1.92E-04 | ns | ns | s5 | ns |
| BovineHD1400013173 | 14 | 46573734 | [rs136611166](http://www.ncbi.nlm.nih.gov/projects/SNP/snp_ref.cgi?rs=136611166) | 0.419643 | 1.30E-05 | ns | ns | s5 | ns |
| BovineHD1400013176 | 14 | 46583429 | [rs132947389](http://www.ncbi.nlm.nih.gov/projects/SNP/snp_ref.cgi?rs=132947389) | 0.704082 | 5.66E-05 | ns | ns | s5 | ns |
| BovineHD1400013177 | 14 | 46590211 | [rs134235216](http://www.ncbi.nlm.nih.gov/projects/SNP/snp_ref.cgi?rs=134235216) | 0.294643 | 5.87E-05 | ns | ns | s5 | ns |
| BovineHD1400013179 | 14 | 46595412 | [rs132689414](http://www.ncbi.nlm.nih.gov/projects/SNP/snp_ref.cgi?rs=132689414) | 0.503827 | 2.60E-04 | ns | ns | s5 | ns |
| BovineHD1400013180 | 14 | 46597569 | [rs110990981](http://www.ncbi.nlm.nih.gov/projects/SNP/snp_ref.cgi?rs=110990981) | 0.496173 | 2.60E-04 | ns | ns | s5 | ns |
| BovineHD1400013181 | 14 | 46600052 | [rs135796162](http://www.ncbi.nlm.nih.gov/projects/SNP/snp_ref.cgi?rs=135796162) | 0.294643 | 5.87E-05 | ns | ns | s5 | ns |
| BovineHD1400013183 | 14 | 46604889 | [rs135040737](http://www.ncbi.nlm.nih.gov/projects/SNP/snp_ref.cgi?rs=135040737) | 0.28699 | 2.05E-05 | ns | ns | s5 | ns |
| BovineHD1400013184 | 14 | 46608266 | [rs136062214](http://www.ncbi.nlm.nih.gov/projects/SNP/snp_ref.cgi?rs=136062214) | 0.294344 | 1.38E-04 | ns | ns | s5 | ns |
| BovineHD1400013189 | 14 | 46629080 | [rs109480456](http://www.ncbi.nlm.nih.gov/projects/SNP/snp_ref.cgi?rs=109480456) | 0.533163 | 2.04E-04 | ns | ns | s5 | ns |
| BovineHD1400013190 | 14 | 46633644 | [rs136045797](http://www.ncbi.nlm.nih.gov/projects/SNP/snp_ref.cgi?rs=136045797) | 0.670918 | 2.45E-05 | ns | ns | s5 | ns |
| BovineHD1400013194 | 14 | 46643037 | [rs133930486](http://www.ncbi.nlm.nih.gov/projects/SNP/snp_ref.cgi?rs=133930486) | 0.793814 | 3.72E-04 | ns | ns | s10 | ns |
| BovineHD1400013196 | 14 | 46648332 | [rs136613853](http://www.ncbi.nlm.nih.gov/projects/SNP/snp_ref.cgi?rs=136613853) | 0.147959 | 1.52E-04 | ns | ns | s5 | ns |
| BovineHD1400013198 | 14 | 46653900 | [rs137684819](http://www.ncbi.nlm.nih.gov/projects/SNP/snp_ref.cgi?rs=137684819) | 0.754476 | 1.33E-04 | ns | ns | s5 | ns |
| BovineHD1400013199 | 14 | 46658037 | [rs133054550](http://www.ncbi.nlm.nih.gov/projects/SNP/snp_ref.cgi?rs=133054550) | 0.71301 | 2.05E-05 | ns | ns | s5 | ns |
| BovineHD1400024493 | 14 | 46660003 | [rs133483556](http://www.ncbi.nlm.nih.gov/projects/SNP/snp_ref.cgi?rs=133483556) | 0.147959 | 1.52E-04 | ns | ns | s5 | ns |
| BovineHD1400013201 | 14 | 46672794 | [rs136236059](http://www.ncbi.nlm.nih.gov/projects/SNP/snp_ref.cgi?rs=136236059) | 0.155612 | 1.66E-04 | ns | ns | s5 | ns |
| BovineHD1400013202 | 14 | 46674846 | [rs136236059](http://www.ncbi.nlm.nih.gov/projects/SNP/snp_ref.cgi?rs=136236059) | 0.19898 | 2.50E-04 | ns | ns | s5 | ns |
| BovineHD1400013203 | 14 | 46679439 | [rs137344980](http://www.ncbi.nlm.nih.gov/projects/SNP/snp_ref.cgi?rs=137344980) | 0.154731 | 1.36E-04 | ns | ns | s5 | ns |
| BovineHD1400013204 | 14 | 46681580 | [rs135804214](http://www.ncbi.nlm.nih.gov/projects/SNP/snp_ref.cgi?rs=135804214) | 0.844388 | 1.66E-04 | ns | ns | s5 | ns |
| BovineHD1400013205 | 14 | 46687966 | [rs136785030](http://www.ncbi.nlm.nih.gov/projects/SNP/snp_ref.cgi?rs=136785030) | 0.844388 | 1.66E-04 | ns | ns | s5 | ns |
| BovineHD1400013207 | 14 | 46693159 | [rs135727060](http://www.ncbi.nlm.nih.gov/projects/SNP/snp_ref.cgi?rs=135727060) | 0.695153 | 1.19E-04 | ns | ns | s5 | ns |
| BovineHD1400013208 | 14 | 46728235 | [rs133436244](http://www.ncbi.nlm.nih.gov/projects/SNP/snp_ref.cgi?rs=133436244) | 0.665816 | 1.55E-05 | ns | ns | s5 | ns |
| BovineHD1400013209 | 14 | 46730713 | [rs134708967](http://www.ncbi.nlm.nih.gov/projects/SNP/snp_ref.cgi?rs=134708967) | 0.336735 | 3.75E-05 | ns | ns | s5 | ns |
| BovineHD1400013210 | 14 | 46733933 | [rs43103204](http://www.ncbi.nlm.nih.gov/projects/SNP/snp_ref.cgi?rs=43103204) | 0.493622 | 1.16E-03 | ns | ns | s10 | ns |
| BovineHD1400013227 | 14 | 46766501 | [rs43116786](http://www.ncbi.nlm.nih.gov/projects/SNP/snp_ref.cgi?rs=43116786) | 0.484694 | 2.05E-04 | ns | ns | s5 | ns |
| BovineHD1400013229 | 14 | 46770241 | [rs134573179](http://www.ncbi.nlm.nih.gov/projects/SNP/snp_ref.cgi?rs=134573179) | 0.688776 | 2.50E-04 | ns | ns | s5 | ns |
| BovineHD1400013269 | 14 | 46889106 | [rs43765470](http://www.ncbi.nlm.nih.gov/projects/SNP/snp_ref.cgi?rs=43765470) | 0.629243 | 2.88E-04 | ns | ns | s5 | ns |
| BovineHD1400013271 | 14 | 46896298 | [rs43765465](http://www.ncbi.nlm.nih.gov/projects/SNP/snp_ref.cgi?rs=43765465) | 0.522959 | 7.68E-05 | ns | ns | s5 | ns |
| BovineHD1400013276 | 14 | 46921191 | [rs41730924](http://www.ncbi.nlm.nih.gov/projects/SNP/snp_ref.cgi?rs=41730924) | 0.528061 | 3.48E-05 | ns | ns | s5 | ns |
| BovineHD1400013284 | 14 | 46954326 | [rs132921952](http://www.ncbi.nlm.nih.gov/projects/SNP/snp_ref.cgi?rs=132921952) | 0.71447 | 2.63E-04 | ns | ns | s5 | ns |
| BovineHD1400013286 | 14 | 46958693 | [rs41731666](http://www.ncbi.nlm.nih.gov/projects/SNP/snp_ref.cgi?rs=41731666) | 0.596939 | 9.46E-04 | ns | ns | s10 | ns |
| BovineHD1400013300 | 14 | 47006975 | [rs135988903](http://www.ncbi.nlm.nih.gov/projects/SNP/snp_ref.cgi?rs=135988903) | 0.193095 | 1.60E-04 | ns | ns | s5 | ns |
| BovineHD1400013306 | 14 | 47022598 | [rs137476333](http://www.ncbi.nlm.nih.gov/projects/SNP/snp_ref.cgi?rs=137476333) | 0.303069 | 2.00E-04 | ns | ns | s5 | ns |
| BovineHD1400013308 | 14 | 47030501 | [rs134435281](http://www.ncbi.nlm.nih.gov/projects/SNP/snp_ref.cgi?rs=134435281) | 0.702046 | 1.93E-04 | ns | ns | s5 | ns |
| BovineHD1400013364 | 14 | 47255027 | [rs136379991](http://www.ncbi.nlm.nih.gov/projects/SNP/snp_ref.cgi?rs=136379991) | 0.80102 | 8.73E-05 | ns | ns | s5 | ns |
| BovineHD1400013398 | 14 | 47373483 | [rs110193675](http://www.ncbi.nlm.nih.gov/projects/SNP/snp_ref.cgi?rs=110193675) | 0.340561 | 4.60E-04 | ns | ns | s10 | ns |
| BovineHD1400013405 | 14 | 47399899 | [rs137686505](http://www.ncbi.nlm.nih.gov/projects/SNP/snp_ref.cgi?rs=137686505) | 0.705357 | 1.93E-04 | ns | ns | s5 | ns |
| BovineHD1400013407 | 14 | 47411991 | [rs137104743](http://www.ncbi.nlm.nih.gov/projects/SNP/snp_ref.cgi?rs=137104743) | 0.26087 | 1.27E-04 | ns | ns | s5 | ns |
| BovineHD1400013409 | 14 | 47417815 | [rs133459595](http://www.ncbi.nlm.nih.gov/projects/SNP/snp_ref.cgi?rs=133459595) | 0.692602 | 2.27E-04 | ns | ns | s5 | ns |
| BovineHD1400013412 | 14 | 47427893 | [rs133292782](http://www.ncbi.nlm.nih.gov/projects/SNP/snp_ref.cgi?rs=133292782) | 0.72449 | 2.57E-04 | ns | ns | s5 | ns |
| BovineHD1400013414 | 14 | 47433566 | [rs137842279](http://www.ncbi.nlm.nih.gov/projects/SNP/snp_ref.cgi?rs=137842279) | 0.729275 | 2.28E-04 | ns | ns | s5 | ns |
| BovineHD1400013415 | 14 | 47435978 | [rs133657412](http://www.ncbi.nlm.nih.gov/projects/SNP/snp_ref.cgi?rs=133657412) | 0.307398 | 2.27E-04 | ns | ns | s5 | ns |
| BovineHD1400013416 | 14 | 47441598 | [rs137422799](http://www.ncbi.nlm.nih.gov/projects/SNP/snp_ref.cgi?rs=137422799) | 0.307398 | 2.27E-04 | ns | ns | s5 | ns |
| BovineHD1400013417 | 14 | 47444099 | [rs136481210](http://www.ncbi.nlm.nih.gov/projects/SNP/snp_ref.cgi?rs=136481210) | 0.307398 | 2.27E-04 | ns | ns | s5 | ns |
| BovineHD1400013422 | 14 | 47456582 | [rs135237919](http://www.ncbi.nlm.nih.gov/projects/SNP/snp_ref.cgi?rs=135237919) | 0.0765306 | 3.19E-04 | ns | ns | s5 | ns |
| BovineHD1400013429 | 14 | 47484800 | [rs135706622](http://www.ncbi.nlm.nih.gov/projects/SNP/snp_ref.cgi?rs=135706622) | 0.298956 | 2.03E-04 | ns | ns | s5 | ns |
| BovineHD1400013436 | 14 | 47518889 | [rs134059325](http://www.ncbi.nlm.nih.gov/projects/SNP/snp_ref.cgi?rs=134059325) | 0.0905612 | 3.26E-04 | ns | ns | s5 | ns |
| BovineHD1400013440 | 14 | 47529603 | [rs137421198](http://www.ncbi.nlm.nih.gov/projects/SNP/snp_ref.cgi?rs=137421198) | 0.69171 | 4.37E-04 | ns | ns | s10 | ns |
| BovineHD1400013445 | 14 | 47554232 | [rs134918760](http://www.ncbi.nlm.nih.gov/projects/SNP/snp_ref.cgi?rs=134918760) | 0.0905612 | 2.86E-04 | ns | ns | s5 | ns |
| BovineHD1400013506 | 14 | 47744774 | [rs136897411](http://www.ncbi.nlm.nih.gov/projects/SNP/snp_ref.cgi?rs=136897411) | 0.756378 | 5.96E-04 | ns | ns | s10 | ns |
| BovineHD1400013522 | 14 | 47794979 | [rs137626055](http://www.ncbi.nlm.nih.gov/projects/SNP/snp_ref.cgi?rs=137626055) | 0.501276 | 5.92E-04 | ns | ns | s10 | ns |
| BovineHD1400013528 | 14 | 47811939 | [rs135308676](http://www.ncbi.nlm.nih.gov/projects/SNP/snp_ref.cgi?rs=135308676) | 0.850765 | 4.01E-04 | ns | ns | s10 | ns |
| BovineHD1400013535 | 14 | 47830208 | [rs133901737](http://www.ncbi.nlm.nih.gov/projects/SNP/snp_ref.cgi?rs=133901737) | 0.315722 | 9.23E-04 | ns | ns | s10 | ns |
| BovineHD1400013556 | 14 | 47879309 | [rs136129377](http://www.ncbi.nlm.nih.gov/projects/SNP/snp_ref.cgi?rs=136129377) | 0.318878 | 7.00E-04 | ns | ns | s10 | ns |
| BovineHD1400013561 | 14 | 47894552 | [rs134361703](http://www.ncbi.nlm.nih.gov/projects/SNP/snp_ref.cgi?rs=134361703) | 0.318878 | 7.00E-04 | ns | ns | s10 | ns |
| BovineHD1400013567 | 14 | 47913038 | [rs136323892](http://www.ncbi.nlm.nih.gov/projects/SNP/snp_ref.cgi?rs=136323892) | 0.711735 | 1.01E-04 | ns | ns | s5 | ns |
| BovineHD1400013568 | 14 | 47915284 | [rs133677985](http://www.ncbi.nlm.nih.gov/projects/SNP/snp_ref.cgi?rs=133677985) | 0.112245 | 3.54E-04 | ns | ns | s10 | ns |
| BovineHD1400013572 | 14 | 47932233 | [rs134780330](http://www.ncbi.nlm.nih.gov/projects/SNP/snp_ref.cgi?rs=134780330) | 0.683673 | 4.22E-04 | ns | ns | s10 | ns |
| BovineHD1400013573 | 14 | 47935011 | [rs137841290](http://www.ncbi.nlm.nih.gov/projects/SNP/snp_ref.cgi?rs=137841290) | 0.317481 | 7.19E-04 | ns | ns | s10 | ns |
| BovineHD4100011520 | 14 | 47999462 | [rs29024079](http://www.ncbi.nlm.nih.gov/projects/SNP/snp_ref.cgi?rs=29024079) | 0.632653 | 6.77E-04 | ns | ns | s10 | ns |
| BovineHD1400013635 | 14 | 48118128 | [rs110326714](http://www.ncbi.nlm.nih.gov/projects/SNP/snp_ref.cgi?rs=110326714) | 0.621173 | 1.20E-03 | ns | ns | s10 | ns |
| BovineHD1400013789 | 14 | 48490212 | [rs41730687](http://www.ncbi.nlm.nih.gov/projects/SNP/snp_ref.cgi?rs=41730687) | 0.441327 | 7.94E-05 | ns | ns | s5 | ns |
| BovineHD1400013790 | 14 | 48491992 | [rs136187291](http://www.ncbi.nlm.nih.gov/projects/SNP/snp_ref.cgi?rs=136187291) | 0.441327 | 7.94E-05 | ns | ns | s5 | ns |
| BovineHD1400013791 | 14 | 48494388 | [rs41730662](http://www.ncbi.nlm.nih.gov/projects/SNP/snp_ref.cgi?rs=41730662) | 0.441327 | 7.94E-05 | ns | ns | s5 | ns |
| BovineHD1400013792 | 14 | 48498279 | [rs109174870](http://www.ncbi.nlm.nih.gov/projects/SNP/snp_ref.cgi?rs=109174870) | 0.441327 | 7.94E-05 | ns | ns | s5 | ns |
| BovineHD1400013885 | 14 | 48765477 | [rs134605857](http://www.ncbi.nlm.nih.gov/projects/SNP/snp_ref.cgi?rs=134605857) | 0.378827 | 4.71E-04 | ns | ns | s10 | ns |
| BovineHD1400013935 | 14 | 48936058 | [rs135065691](http://www.ncbi.nlm.nih.gov/projects/SNP/snp_ref.cgi?rs=135065691) | 0.197704 | 8.27E-04 | ns | ns | s10 | ns |
| BovineHD1400013939 | 14 | 48949695 | [rs41734435](http://www.ncbi.nlm.nih.gov/projects/SNP/snp_ref.cgi?rs=41734435) | 0.539788 | 9.48E-04 | ns | ns | s10 | ns |
| BovineHD1400013946 | 14 | 48968947 | [rs135292147](http://www.ncbi.nlm.nih.gov/projects/SNP/snp_ref.cgi?rs=135292147) | 0.823077 | 9.18E-04 | ns | ns | s10 | ns |
| BovineHD1400014135 | 14 | 49776551 | [rs134885647](http://www.ncbi.nlm.nih.gov/projects/SNP/snp_ref.cgi?rs=134885647) | 0.222222 | 6.45E-04 | ns | ns | s10 | ns |
| BovineHD1400014138 | 14 | 49782421 | [rs135196057](http://www.ncbi.nlm.nih.gov/projects/SNP/snp_ref.cgi?rs=135196057) | 0.742627 | 1.03E-03 | ns | ns | s10 | ns |
| BovineHD1400014142 | 14 | 49798506 | [rs132844337](http://www.ncbi.nlm.nih.gov/projects/SNP/snp_ref.cgi?rs=132844337) | 0.770513 | 4.32E-04 | ns | ns | s10 | ns |
| BovineHD1400014233 | 14 | 50166203 | [rs134994711](http://www.ncbi.nlm.nih.gov/projects/SNP/snp_ref.cgi?rs=134994711) | 0.725765 | 4.85E-04 | ns | ns | s10 | ns |
| BovineHD1400014234 | 14 | 50169555 | [rs136618636](http://www.ncbi.nlm.nih.gov/projects/SNP/snp_ref.cgi?rs=136618636) | 0.273077 | 5.28E-04 | ns | ns | s10 | ns |
| BovineHD1400014284 | 14 | 50304203 | [rs110259226](http://www.ncbi.nlm.nih.gov/projects/SNP/snp_ref.cgi?rs=110259226) | 0.297194 | 1.20E-03 | ns | ns | s10 | ns |
| BovineHD1400014315 | 14 | 50411927 | [rs136823095](http://www.ncbi.nlm.nih.gov/projects/SNP/snp_ref.cgi?rs=136823095) | 0.227513 | 3.76E-04 | ns | ns | s10 | ns |
| BovineHD1400014325 | 14 | 50461710 | [rs135380467](http://www.ncbi.nlm.nih.gov/projects/SNP/snp_ref.cgi?rs=135380467) | 0.77551 | 2.76E-04 | ns | ns | s5 | ns |
| BovineHD1400014370 | 14 | 50605946 | [rs137450084](http://www.ncbi.nlm.nih.gov/projects/SNP/snp_ref.cgi?rs=137450084) | 0.814103 | 1.73E-04 | ns | ns | s5 | ns |
| BovineHD1400014489 | 14 | 51021072 | [rs110259226](http://www.ncbi.nlm.nih.gov/projects/SNP/snp_ref.cgi?rs=110259226) | 0.752551 | 1.09E-03 | ns | ns | s10 | ns |
| BovineHD1400014521 | 14 | 51121965 | [rs137452104](http://www.ncbi.nlm.nih.gov/projects/SNP/snp_ref.cgi?rs=137452104) | 0.273657 | 3.72E-04 | ns | ns | s10 | ns |
| BovineHD1400014527 | 14 | 51144199 | [rs134705032](http://www.ncbi.nlm.nih.gov/projects/SNP/snp_ref.cgi?rs=134705032) | 0.771684 | 1.02E-03 | ns | ns | s10 | ns |
| BovineHD1400014531 | 14 | 51157693 | [rs137604610](http://www.ncbi.nlm.nih.gov/projects/SNP/snp_ref.cgi?rs=137604610) | 0.196429 | 3.26E-05 | ns | ns | s5 | ns |
| BTB-00571421 | 14 | 53749131 | [rs41737198](http://www.ncbi.nlm.nih.gov/projects/SNP/snp_ref.cgi?rs=41737198) | 0.252551 | 8.47E-04 | ns | ns | s10 | ns |
| BovineHD1400015122 | 14 | 53754300 | [rs111015451](http://www.ncbi.nlm.nih.gov/projects/SNP/snp_ref.cgi?rs=111015451) | 0.747449 | 8.47E-04 | ns | ns | s10 | ns |
| BovineHD1400015125 | 14 | 53772426 | [rs136124553](http://www.ncbi.nlm.nih.gov/projects/SNP/snp_ref.cgi?rs=136124553) | 0.252551 | 8.47E-04 | ns | ns | s10 | ns |
| BovineHD1400015136 | 14 | 53814543 | [rs43727549](http://www.ncbi.nlm.nih.gov/projects/SNP/snp_ref.cgi?rs=43727549) | 0.251276 | 6.95E-04 | ns | ns | s10 | ns |
| BovineHD4100011554 | 14 | 55755269 | [rs110134306](http://www.ncbi.nlm.nih.gov/projects/SNP/snp_ref.cgi?rs=110134306) | 0.871173 | 9.34E-04 | ns | ns | s10 | ns |
| BovineHD4100011639 | 14 | 57524221 | [rs133457508](http://www.ncbi.nlm.nih.gov/projects/SNP/snp_ref.cgi?rs=133457508) | 0.560102 | 2.27E-04 | ns | ns | s5 | ns |
| BovineHD1400016619 | 14 | 59969985 | [rs136790655](http://www.ncbi.nlm.nih.gov/projects/SNP/snp_ref.cgi?rs=136790655) | 0.841026 | 2.52E-04 | ns | ns | s5 | ns |
| BovineHD1400022035 | 14 | 78593987 | [rs134287222](http://www.ncbi.nlm.nih.gov/projects/SNP/snp_ref.cgi?rs=134287222) | 0.675192 | 8.74E-04 | ns | ns | s10 | ns |
| BovineHD1800006143 | 18 | 19853034 | [rs134971286](http://www.ncbi.nlm.nih.gov/projects/SNP/snp_ref.cgi?rs=134971286) | 0.474425 | 3.36E-06 | s10 | ns | s1 | s5 |
| BovineHD1800006144 | 18 | 19853948 | [rs132817573](http://www.ncbi.nlm.nih.gov/projects/SNP/snp_ref.cgi?rs=132817573) | 0.474425 | 3.36E-06 | s10 | ns | s1 | s5 |
| BovineHD1800006145 | 18 | 19859849 | [rs137143517](http://www.ncbi.nlm.nih.gov/projects/SNP/snp_ref.cgi?rs=137143517) | 0.603846 | 1.57E-06 | s5 | ns | s1 | s5 |
| BovineHD1800006149 | 18 | 19876065 | [rs136209413](http://www.ncbi.nlm.nih.gov/projects/SNP/snp_ref.cgi?rs=136209413) | 0.609974 | 2.30E-06 | s5 | ns | s1 | s5 |
| BovineHD1800006150 | 18 | 19877247 | [rs133504320](http://www.ncbi.nlm.nih.gov/projects/SNP/snp_ref.cgi?rs=133504320) | 0.556266 | 5.55E-07 | s1 | ns | s1 | s5 |
| BovineHD1800006152 | 18 | 19879636 | [rs134860120](http://www.ncbi.nlm.nih.gov/projects/SNP/snp_ref.cgi?rs=134860120) | 0.566496 | 7.84E-07 | s5 | ns | s1 | s5 |
| BovineHD4100013512 | 18 | 19880724 | [rs41660719](http://www.ncbi.nlm.nih.gov/projects/SNP/snp_ref.cgi?rs=41660719) | 0.442308 | 3.53E-07 | s1 | ns | s1 | s5 |
| BovineHD1800006153 | 18 | 19881483 | [rs136144630](http://www.ncbi.nlm.nih.gov/projects/SNP/snp_ref.cgi?rs=136144630) | 0.431034 | 7.18E-07 | s5 | ns | s1 | s5 |
| BovineHD1800006154 | 18 | 19882167 | [rs133323595](http://www.ncbi.nlm.nih.gov/projects/SNP/snp_ref.cgi?rs=133323595) | 0.433504 | 7.84E-07 | s5 | ns | s1 | s5 |
| BovineHD1800006156 | 18 | 19883888 | [rs137316303](http://www.ncbi.nlm.nih.gov/projects/SNP/snp_ref.cgi?rs=137316303) | 0.556266 | 5.55E-07 | s1 | ns | s1 | s5 |
| BovineHD1800006157 | 18 | 19884993 | [rs110127001](http://www.ncbi.nlm.nih.gov/projects/SNP/snp_ref.cgi?rs=110127001) | 0.443734 | 5.55E-07 | s1 | ns | s1 | s5 |
| BovineHD2100017853 | 21 | 61415689 | [rs109144916](http://www.ncbi.nlm.nih.gov/projects/SNP/snp_ref.cgi?rs=109144916) | 0.115681 | 1.99E-06 | s5 | ns | s5 | s5 |
| BovineHD4100015309 | 21 | 61423440 | [rs29012774](http://www.ncbi.nlm.nih.gov/projects/SNP/snp_ref.cgi?rs=29012774) | 0.882653 | 2.45E-06 | s5 | ns | s5 | s5 |
| BovineHD2100017857 | 21 | 61431358 | [rs110875395](http://www.ncbi.nlm.nih.gov/projects/SNP/snp_ref.cgi?rs=110875395) | 0.882653 | 2.45E-06 | s5 | ns | s5 | s5 |
| BovineHD2100017858 | 21 | 61437160 | [rs109446125](http://www.ncbi.nlm.nih.gov/projects/SNP/snp_ref.cgi?rs=109446125) | 0.116368 | 1.78E-06 | s5 | ns | s5 | s5 |

MAF = minor allele frequency.

s10 = 10% significance level.

s5 = 5% significance level.

ns = not significantly different from zero.
