# Supplementary material for: Candidate genes for male and female reproductive traits in Canchim beef cattle
Source: J Anim Sci Biotechnol. 2017 Aug 23;8:67. doi: 10.1186/s40104-017-0199-8 (PMC5569548; doi:10.1186/s40104-017-0199-8)
Supplement: Supplementary file 2 — Genome-wide (in bold) and chromosome-wise associations for scrotal circumference at 420 days of age. Gene symbols, SNP reference number, and chromosomes (Chr) and positions (Pos, in megabase) were obtained from NCBI website. Distances to gene (kilobase) are presented from 5′ to gene and 3′ to gene directions. If distance equals zero (0.00), the SNP is on intragenic region. P-values are presented as the minimum (Min) and maximum (Max) significance obtained from the generalized quasi-likelihood method. (DOCX 34 kb) [file 40104_2017_199_MOESM2_ESM.docx]

**Additional file 2.** Genome-wide (in bold) and chromosome-wise associations for scrotal circumference at 420 days of age. Gene symbols, SNP reference number, and chromosomes (Chr) and positions (Pos, in megabase) were obtained from NCBI website. Distances to gene (kilobase) are presented from 5’ to gene and 3’ to gene directions. If distance equals zero (0.00), the SNP is on intragenic region. P-values are presented as the minimum (Min) and maximum (Max) significance obtained from the generalized quasi-likelihood method.

| Symbol | Category | SNP Reference | Chr:Pos | Distances to gene | P-value (Min - Max) |
| --- | --- | --- | --- | --- | --- |
| *SYT1* | Gene | rs43429788^1^ | 5:8.99 | 0.00 | 9.45E-05 |
| *PUS7L* | Gene | rs43434300^2^ | 5:36.93 | 7.54 | 1.01E-04 |
| *LOC617654* | Pseudogene | rs133344101^2^, rs137418499^2^, rs132691450^2^, rs134381968^2^, rs135563280^2^, rs135563280^2^, rs110587794^2^ | 5:42.22..42.27 | 7.13..55.21 | 3.60E-04 - 1.86E-04 |
| *CPM* | Gene | rs134557648^1^, rs136126069^1^ | 5:45.10..45.10 | 0.00 | 1.65E-05 - 2.18E-04 |
| *SLC35E3* | Gene | rs136466664^3^, rs134969150^1^, rs136251215^1^, rs136974465^1^, rs134653913^1^, rs137721339^1^, rs133985145^1^, rs135251296^1^, **rs132874802**^1^, **rs109156482**^1^, rs110291739^4^ | 5:45.25..45.27 | 0.00..4.40 | 8.17E-06 - 3.01E-04 |
| *NUP107* | Gene | rs110572792^5^, rs110698350^1^, rs134370690^1^, **rs109284796**^1^, rs110852214^1^, rs137552662^1^, **rs108983599**^1^, rs41595547^1^, rs137318696^1^, rs134463406^1^, rs133818805^2^ | 5:45.27..45.33 | 11.00..0.00..7.75 | 5.59E-06 - 3.89E-04 |
| *RAP1B* | Gene | **rs110520377**^2^, **rs133990240**^2^, rs109547215^2^, **rs110261691**^5^, **rs110160018**^1^, **rs109099268**^1^, **rs133124963**^1^, **rs109023687**^1^, **rs110034677**^1^, **rs110091099**^1^, **rs109950552**^1^, **rs137658592**^1^, rs133340933^1^, rs134626455^1^, rs110001336^1^, **rs109288126**^1^, rs137319832^1^, **rs109506571**^1^, **rs109248631**^1^, **rs110027103**^1^, **rs109561643**^1^**, rs109210079**^1^, **rs110160918**^1^, rs134366426^1^, rs109273768^1^, rs109024096^1^, rs110798702^1^, **rs110625630**^1^, **rs110219262**^1^, **rs134311132**^1^, **rs135497432**^1^, **rs133173059**^1^ | 5:45.35..45.40 | 8.58..0.00 | 2.47E-07 - 7.64E-05 |
| *IFNG* | Gene | rs109941119^2^, rs110443290^2^, rs110739783^2^, rs133006321^2^, rs136589101^2^, rs41590069^2^, rs109635265^2^, rs109461229^2^, rs110502555^2^, rs109042529^2^ | 5:45.88..45.99 | 45.44..153.61 | 1.73E-04 - 4.09E-04 |
| *LLPH* | Gene | rs137227247^2^ | 5:47.92 | 16.14 | 2.85E-05 |
| *WIF1* | Gene | rs135293584^1^, rs134846637^2^ | 5:48.93..49.04 | 0.00..29.03 | 2.08E-04 - 2.12E-04 |
| *TBC1D30* | Gene | rs109818155^2^, rs109388550^2^ | 5:49.10..49.10 | 78.62..76.41 | 3.78E-04 |
| *SRGAP1* | Gene | **rs110268648**^1^, **rs109748105**^1^, **rs134621421**^1^ | 5:49.98..49.98 | 0.00 | 1.88E-06 - 6.55E-06 |
| *TMEM5* | Gene | rs137166674^2^, rs134596971^2^, rs109404421^2^, rs137735429^2^, rs137317884^2^, rs136285326^2^, rs133604447^2^ | 5:50.32..50.35 | 146.14..177.93 | 4.50E-05 - 4.10E-04 |
| *PPM1H* | Gene | rs110034898^2^ | 5:50.81 | 50.32 | 2.88E-04 |
| *FAM19A2* | Gene | rs133712981^2^, rs133468722^2^, rs108942252^2^, rs110821453^2^, rs137102880^2^, **rs133590688**^2^, rs109729395^2^, rs109494203^2^, rs109103723^2^, rs109036488^2^, rs132720145^2^, rs135762369^2^ | 5:52.35..52.66 | 188.78..490.33 | 4.35E-06 - 3.62E-04 |
| *LOC788961* | Pseudogene | rs110654445^2^, **rs132843988**^2^, **rs134781821**^2^, rs110197988^2^, **rs110906264**^2^, rs109124151^2^, rs110767141^2^, rs109572573^2^, rs110762622^2^ | 5:52.76..52.80 | 453.63..411.61 | 2.45E-06 - 1.40E-04 |
| *SLC16A7* | Gene | rs137139553^2^, rs135522177^2^, rs109062985^2^, rs110673933^2^, rs133769998^2^, rs134919998^1^, rs109455016^1^ | 5:53.73..54.13 | 256.97.. 0.00 | 8.52E-05 - 4.18E-04 |
| *LOC101907520* | Non-coding RNA | rs110845453^2^ | 5:54.31 | 66.16 | 3.97E-04 |
| *LOC785078* | Pseudogene | rs110311658^2^, rs109035507^2^, rs135645960^2^, rs133048773^2^, rs137246199^2^, rs136147741^2^ | 5:54.61..54.76 | 244.76..97.58 | 1.13E-04 - 3.98E-04 |
| *ANO4* | Gene | rs132893231^2^ | 5:64.95 | 8.98 | 1.29E-04 |
| *CKAP4* | Gene | rs134969556^2^ | 5:69.95 | 23.81 | 1.81E-04 |
| *SOX5* | Gene | rs43445896^1^ | 5:86.09 | 0.00 | 1.77E-04 |
| *LOC100139060* | Pseudogene | rs109408384^2^ | 5:96.18 | 29.35 | 6.79E-05 |
| *BCL2L14* | Gene | rs136476913^2^ | 5:98.27 | 32.19 | 2.78E-04 |
| *CD69* | Gene | rs110460517^1^ | 5:100.74 | 0.00 | 1.13E-04 |
| *FOXM1* | Gene | rs135705262^1^ | 5:107.39 | 0.00 | 4.00E-04 |
| *COL12A1* | Gene | **rs43582369**^2^ | 9:15.01 | 22.34 | 8.11E-06 |
| *IMPG1* | Gene | **rs1101305560**^1^, **rs43582288**^6^, **rs43582290**^1^ | 9:15.88..15.88 | 0.00 | 6.66E-07 - 1.72E-06 |
| *BCKDHB* | Gene | **rs43587922**^2^, **rs110633796**^2^ | 9:20.51..20.51 | 172.87..175.14 | 4.26E-06 |
| *CLVS2* | Gene | rs42242832^2^ | 9:28.60 | 16.88 | 2.58E-05 |
| *LOC104970694* | Gene | rs137387359^2^, rs137153440^2^, **rs135164150**^2^, rs132757037^2^ | 9:93.70..93.71 | 121.11..114.81 | 2.94E-06 - 2.94E-05 |
| *TRNAR-UCU* | Transfer RNA | **rs134336444**^2^ | 13:69.51 | 42.30 | 5.89E-06 |
| *TOP1* | Gene | **rs134822694**^1^, **rs135287766**^1^ | 13:70.39..70.40 | 0.00 | 3.86E-06 |
| *LOC101907320* | Non-coding RNA | rs133273718^1^ | 13:70.49 | 0.00 | 2.11E-05 |
| *CHD6* | Gene | rs137564189^2^ | 13:70.93 | 3.04 | 2.35E-05 |
| *ZNF16* | Gene | rs110090404^2^ | 14:1.46 | 4.19 | 1.02E-03 |
| *ST3GAL1* | Gene | rs133748371^2^, rs109480281^1^ | 14:8.86..8.91 | 35.34..0.00 | 3.46E-05 - 6.53E-04 |
| *ASAP1* | Gene | rs110751858^1^ | 14:11.33 | 0.00 | 1.55E-04 |
| *ZHX2* | Gene | rs133961677^1^, r s109618600^1^, rs111000599^1^ | 14:18.42..18.47 | 0.00 | 1.79E-04 - 1.06E-03 |
| *MIR124A-2* | Non-coding RNA | rs134424688^2^ | 14:30.87 | 57.27 | 9.25E-04 |
| *CYP7B1* | Gene | rs133066519^1^ | 14:31.10 | 0.00 | 8.37E-04 |
| *C14H8orf34* | Gene | rs132764742^1^ | 14:34.76 | 0.00 | 1.12E-03 |
| *LACTB2* | Gene | rs135075316^1^ | 14:36.45 | 0.00 | 2.66E-04 |
| *LOC783431* | Pseudogene | rs136664344^2^ | 14:36.69 | 66.80 | 1.49E-04 |
| *EYA1* | Gene | rs134745604^2^, rs137743952^2^, rs134361509^2^, rs136612113^2^, rs134103447^2^, rs137681879^2^ | 14:36.83..36.86 | 70.43..36.25 | 1.07E-04 - 1.13E-03 |
| *TRNAC-ACA* | Transfer RNA | rs110656764^2^, rs133726980^2^ | 14:37.34..37.39 | 6.18..38.60 | 9.45E-04..9.91E-04 |
| *MSC* | Gene | rs137187252^2^, rs134037510^2^, rs135657472^2^, rs134109294^2^, rs135448359^2^, rs133171963^4^ | 14:37.47..37.55 | 81.27..0.00 | 6.09E-04 - 1.15E-03 |
| *LOC101903752* | Non-coding RNA | rs135238827^2^ | 14:37.60 | 38.37 | 1.20E-03 |
| *TRPA1* | Gene | rs132804124^2^, rs137073386^2^, rs135938474^2^, rs137070738^2^ | 14:37.86..37.86 | 11.96..18.05 | 1.57E-05 - 6.99E-05 |
| *STAU2* | Gene | rs137465376^1^, rs134711539^1^, rs137821036^2^ | 14:38.89..38.97 | 0.00..14.40 | 8.66E-05 - 5.00E-04 |
| *GDAP1* | Gene | rs109995322^2^ | 14:39.91 | 136.91 | 1.10E-03 |
| *ZFHX4* | Gene | rs134984970^2^, rs42142739^4^, rs42142629^1^, rs134584620^1^, rs134053575^2^ | 14:41.98..42.21 | 7.68..0.00..17.60 | 2.91E-04 - 1.19E-03 |
| *PEX2* | Gene | rs137442228^7^, rs110035827^2^, rs41730291^2^ | 14:42.33..42.37 | 0.00..40.77 | 4.10E-04 - 9.80E-04 |
| *LOC782385* | Non-coding RNA | rs137101443^2^, rs136174931^2^, rs42365139^2^, rs137216648^2^, rs42856291^2^, rs42856280^2^, rs42856277^2^, rs133558145^2^, rs137391994^2^, rs135129301^2^, rs136589835^2^, rs42856277^2^, rs133394363^2^ | 14:42.86..43.14 | 100.40.. 158.72 | 2.14E-04 - 1.12E-03 |
| *LOC780982* | Pseudogene | rs133748595^2^ | 14:43.82 | 38.43 | 3.63E-04 |
| *PKIA* | Gene | rs137048645^2^, rs135901170^2^, rs136656767^2^ | 14:43.87..43.87 | 14.40..6.35 | 8.11E-04 - 1.04E-03 |
| *IL7* | Gene | rs135173508^1^, rs135983396^2^, rs136887798^2^, rs135984324^2^, rs136708459^2^, rs133383682^2^, rs132630954^2^ | 14:44.16..44.46 | 0.00.. 274.51 | 6.39E-04 - 1.05E-03 |
| *STMN2* | Gene | rs133243977^2^, rs137737620^2^ | 14:44.68..44.70 | 284.28..269.05 | 3.87E-04 - 1.07E-03 |
| *HEY1* | Gene | rs132803686^5^, rs136891270^6^, rs134354120^2^ | 14:45.12..45.13 | 2.82..0.00 | 2.83E-04 - 2.95E-04 |
| *MRPS28* | Gene | rs133901332^2^, rs134064937^1^, rs135297170^1^, rs136659909^1^, rs134211847^1^, rs137570609^1^, rs133401075^1^, rs110113985^1^, rs110112317^1^, **rs133715055**^1^, **rs109442520**^1^, **rs136608009**^1^, rs136372877^1^, rs110643403^1^, rs134638942^1^, rs137572973^1^, rs133174611^1^, rs137339424^1^, rs135782413^1^, rs134521313^1^, rs136159423^1^, rs133881536^1^, rs134722896^1^, rs137744254^1^, rs137510178^1^, rs133002930^1^, rs134041698^1^, rs135639560^1^, rs132639160^1^, rs135760404^1^, rs134062262^1^, rs137679118^1^, rs133399056^1^, rs133080568^1^, rs137341841^1^, rs136942595^1^ | 14:45.27..45.49 | 9.80.. 0.00 | 1.02E-06 - 1.09E-03 |
| *TPD52* | Gene | rs134553723^5^, rs136546448^5^, rs134233283^1^, rs136442964^1^, rs133649827^1^, rs137512750^1^, rs137113350^1^, rs135047163^1^, rs110246732^1^, **rs110610723**^1^, rs136434991^2^ | 14:45.50..45.57 | 2.48..0.00..20.14 | 2.15E-06 - 1.19E-03 |
| *LOC100138499* | Pseudogene | rs137690798^2^, rs132812346^2^, rs135301640^2^, rs135609702^2^, rs136719533^2^, rs136324758^2^, rs133469839^2^, rs137287756^2^ | 14:45.61..45.69 | 46.59..3.25..26.28 | 6.41E-05 - 1.11E-03 |
| *LOC100295528* | Pseudogene | rs133719470^2^, rs137458332^2^, rs132804447^2^ | 14:45.70..45.73 | 13.93..9.72..14.22 | 7.13E-05..4.17E-04 |
| *LOC785035* | Pseudogene | rs135426196^2^, rs136495820^2^ | 14:45.97..45.97 | 21.14..25.36 | 5.24E-04 - 6.52E-04 |
| *ZNF704* | Gene | rs133781069^2^, rs137400994^2^, rs135967036^2^, rs137747048^1^, rs109754076^1^, rs136612230^1^, rs110667294^1^, rs134236615^1^, rs133086073^2^, rs134247697^2^, rs136445928^2^, rs109900017^2^, rs136496010^2^, rs134044871^2^, rs137695047^2^, rs110036121^2^ | 14:45.98..46.26 | 14.97..0.00..76.52 | 4.62E-05 - 1.10E-03 |
| *PAG1* | Gene | rs109344263^2^, rs137459798^2^, rs135311524^2^, rs132944088^2^, rs137120971^2^, rs134407664^2^, rs137631985^2^, rs135035289^2^, rs136384647^2^, rs133634325^2^, **rs137291182**^5^, rs136894433^1^, rs134868727^1^, rs137462920^2^, rs137066271^2^, **rs134665936**^2^, rs133332165^2^, rs135721856^2^, rs133794575^2^, rs135664659^2^, rs132652656^2^, rs135858520^2^, rs132821440^2^, rs137068943^2^ | 14:46.26..46.44 | 68.08..0.00..82.60 | 1.64E-06 -8.18E-04 |
| *LOC101905560* | Non-coding RNA | rs133091332^2^, rs41629829^2^, rs109185265^2^, rs110094547^2^, rs137006524^2^, rs136665695^2^, rs110270638^2^, rs134364376^2^, rs110881709^2^ | 14:46.46..46.52 | 68.94..7.81 | 1.71E-05 - 8.75E-04 |
| *LOC101905608* | Non-coding RNA | rs134723944^1^, rs136611166^1^, rs132947389^1^, rs134235216^1^, rs132689414^2^, rs110990981^2^, rs135796162^2^, rs135040737^2^, rs136062214^2^ | 14:46.53..46.61 | 0.00..15.74 | 1.30E-05 - 2.60E-04 |
| *FABP5* | Gene | rs109480456^2^, rs136045797^2^, rs133930486^4^, rs136613853^1^, rs137684819^5^, rs133054550^2^, rs133483556^2^, rs136236059^2^, rs136236059^2^, rs137344980^2^, rs135804214^2^, rs136785030^2^, rs135727060^2^, rs133436244^2^, rs134708967^2^, rs43103204^2^ | 14:46.63..46.73 | 15.48..0.00..83.31 | 1.55E-05 - 1.16E-03 |
| *PMP2* | Gene | rs43116786^2^, rs134573179^2^ | 14:46.77..46.77 | 35.27..31.53 | 2.05E-04..2.50E-04 |
| *FABP12* | Gene | rs43765470^1^, rs43765465^2^, rs41730924^2^ | 14:46.89..46.92 | 0.00..28.04 | 3.48E-05 - 2.88E-04 |
| *NOV* | Gene | rs132921952^2^, rs41731666^2^, rs135988903^6^, rs137476333^2^, rs134435281^2^ | 14:46.95..47.03 | 51.23..0.00..16.57 | 1.60E-04 - 9.46E-04 |
| *COLEC10* | Gene | rs136379991^1^ | 14:47.26 | 0.00 | 8.73E-05 |
| *TNFRSF11B* | Gene | rs110193675^2^, rs137686505^2^, rs137104743^2^, rs133459595^2^, rs133292782^2^, rs137842279^1^, rs133657412^1^, rs137422799^1^, rs136481210^1^, rs135237919^1^ | 14:47.37..47.46 | 59.42.. 0.00 | 1.27E-04 - 4.60E-04 |
| *LOC101905780* | Non-coding RNA | rs135706622^1^, rs134059325^1^, rs137421198^1^, rs134918760^1^, rs136897411^1^ | 14:47.48..47.74 | 0.00 | 2.03E-04 - 5.96E-04 |
| *SAMD12* | Gene | rs137626055^1^, rs135308676^1^, rs133901737^1^, rs136129377^1^, rs134361703^1^, rs136323892^1^, rs133677985^1^, rs134780330^1^, rs137841290^1^, rs29024079^1^, rs110326714^1^ | 14:47.79..48.12 | 0.00 | 1.01E-04 - 1.20E-03 |
| *EXT1* | Gene | rs41730687^1^, rs136187291^1^, rs41730662^1^, rs109174870^1^, rs134605857^2^ | 14:48.49..48.77 | 0.00..131.45 | 7.94E-05 - 4.71E-04 |
| *MED30* | Gene | rs135065691^5^, rs41734435^1^, rs135292147^2^ | 14:48.94..48.97 | 4.03..0.00..9.76 | 8.27E-04 - 9.48E-04 |
| *EIF3H* | Gene | rs134885647^1^, rs135196057^1^, rs132844337^1^ | 14:49.78..49.80 | 0.00 | 4.32E-04 - 1.03E-03 |
| *LOC101907479* | Pseudogene | rs134994711^2^, rs136618636^2^, rs110259226^2^ | 14:50.17..50.30 | 176.54..314.54 | 4.85E-04 - 1.20E-03 |
| *LOC781434* | Gene | rs136823095^2^, rs135380467^2^, rs137450084^2^, rs110259226^2^ | 14:50.41..50.30 | 194.12..0.10..301.84 | 1.73E-04 - 1.09E-03 |
| *TRPS1* | Gene | rs137452104^2^, rs134705032^2^ | 14:51.12..51.14 | 55.77..78.00 | 3.72E-04 - 1.02E-03 |
| *LOC100138031* | Pseudogene | rs137604610^2^ | 14:51.16 | 75.61 | 3.26E-05 |
| *LOC101905320* | Pseudogene | rs41737198^2^, rs111015451^2^, rs136124553^2^, rs43727549^2^ | 14:53.75..53.81 | 194.69.. 129.28 | 6.95E-04 - 8.47E-04 |
| *LOC101905373* | Non-coding RNA | rs110134306^2^ | 14:55.76 | 97.50 | 9.34E-04 |
| *TRHR* | Gene | rs133457508^4^ | 14:57.52 | 0.00 | 2.27E-04 |
| *LOC100141107* | Pseudogene | rs136790655^2^ | 14:59.97 | 0.31 | 2.52E-04 |
| *RMDN1* | Gene | rs134287222^2^ | 14:78.59 | 0.36 | 8.74E-04 |
| *LOC101906490* | Non-coding RNA | **rs134971286**^2^, **rs132817573**^2^, **rs137143517**^2^, **rs136209413**^2^, **rs133504320**^2^, **rs134860120**^2^, **rs41660719**^2^, **rs136144630**^2^, **rs133323595**^2^, **rs137316303**^2^, **rs110127001**^2^ | 18:19.85..19.88 | 12.23..9.53 | 3.53E-07 - 3.36E-06 |
| *DICER1* | Gene | **rs109144916**^2^, **rs29012774**^2^, **rs110875395**^2^, **rs109446125**^2^ | 21:61.42..21:61.44 | 97.29.. 75.82 | 1.78E-06 - 2.45E-06 |

^1^intron variant.

^2^intergenic region.

^3^3'UTR.

^4^upstream variant.

^5^downstream variant.

^6^synonymous variant.

^7^5'UTR.
