# Supplementary material for: Candidate genes for male and female reproductive traits in Canchim beef cattle
Source: J Anim Sci Biotechnol. 2017 Aug 23;8:67. doi: 10.1186/s40104-017-0199-8 (PMC5569548; doi:10.1186/s40104-017-0199-8)
Supplement: Supplementary file 3 — Candidate genes identified for scrotal circumference at 420 days of age. (DOCX 26 kb) [file 40104_2017_199_MOESM3_ESM.docx]

**Additional file 3.** Candidate genes identified for scrotal circumference at 420 days of age.

| **Gene Symbol** | **Official gene name** | **Gene ID** | **BTA** | **Position (UMD 3.1)** | **PANTHER Protein Class** |
| --- | --- | --- | --- | --- | --- |
| *ANO4* | *anoctamin 4* | 518739 | 5 | 64928004..65361839 | -- |
| *ASAP1* | *ArfGAP with SH3 domain, ankyrin repeat and PH domain 1* | 327705 | 14 | 11327981..11696284 | nucleic acid binding, G-protein modulator |
| *BCKDHB* | *branched chain keto acid dehydrogenase E1, beta polypeptide* | 282150 | 9 | 19963215..20335161 | transketolase, dehydrogenase, lyase |
| *BCL2L14* | *BCL2-like 14 (apoptosis facilitator)* | 508365 | 5 | 98196359..98233616 | -- |
| *C14H8orf34* | *chromosome 14 open reading frame, human C8orf34* | 615152 | 14 | 34427576..34830956 | -- |
| *CD69* | *CD69 molecule* | 281058 | 5 | 100740574..100747670 | membrane-bound signaling molecule, receptor, |
|  |  |  |  |  | defense/immunity protein |
| *CHD6* | *chromodomain helicase DNA binding protein 6* | 524776 | 13 | 70727182..70926564 | DNA helicase |
| *CKAP4* | *cytoskeleton-associated protein 4* | 515784 | 5 | 69971127..69979910 | chromatin/chromatin-binding protein, hydrolase |
| *CLVS2* | *clavesin 2* | 534359 | 9 | 28618226..28695359 | transfer/carrier protein, dehydrogenase |
| *COL12A1* | *collagen, type XII, alpha 1* | 359712 | 9 | 14868630..14987696 | receptor, extracellular matrix protein, cell adhesion molecule |
| *COLEC10* | *collectin sub-family member 10 (C-type lectin)* | 512030 | 14 | 47261010..47437196 | surfactante, defense/immunity protein |
| *CPM* | *carboxypeptidase M* | 513281 | 5 | 45081453..45162177 | metalloprotease |
| *CYP7B1* | *cytochrome P450, family 7, subfamily B, polypeptide 1* | 529552 | 14 | 30977916..31154030 | oxygenase |
| *DICER1* | *dicer 1, ribonuclease type III* | 337871 | 21 | 61508626..61583944 | endodeoxyribonuclease, nuclease |
| *EIF3H* | *eukaryotic translation initiation factor 3, subunit H* | 506873 | 14 | 49733172..49830427 | transcription factor |
| *EXT1* | *exostosin glycosyltransferase 1* | 538602 | 14 | 48318418..48634025 | glycosyltransferase |
| *EYA1* | *EYA transcriptional coactivator and phosphatase 1* | 511188 | 14 | 36896160..37268911 | -- |
| *FABP12* | *fatty acid binding protein 12* | 100300024 | 14 | 46883400..46893169 | transfer/carrier protein |
| *FABP5* | *fatty acid binding protein 5 (psoriasis-associated)* | 281760 | 14 | 46644559..46650621 | transfer/carrier protein |
| *FAM19A2* | *family with sequence similarity 19 (chemokine (C-C motif)-like), member A2* | 100297656 | 5 | 51600529..52166058 | -- |
| *FOXM1* | *forkhead box M1* | 513643 | 5 | 107384361..107398326 | transcription factor, DNA binding protein |
| *GDAP1* | *ganglioside-induced differentiation-associated protein 1* | 613472 | 14 | 39757054..39776014 | transferase, signaling molecule, reductase, translation elongation factor, epimerase/racemase, cytoskeletal protein |
| *HEY1* | *hes-related family bHLH transcription factor with YRPW motif 1* | 408005 | 14 | 45126101..45128997 | basic helix-loop-helix transcription factor, nucleic acid binding |
| *IFNG* | *interferon, gamma* | 281237 | 5 | 45830158..45834981 | interferon superfamily |
| *IL7* | *interleukin 7* | 280827 | 14 | 44095419..44187918 | interleukin superfamily, |
| *IMPG1* | *interphotoreceptor matrix proteoglycan 1* | 281866 | 9 | 15798534..15802384 | receptor, extracellular matrix glycoprotein |
| *LACTB2* | *lactamase, beta 2* | 535021 | 14 | 36438465..36470764 | hydrolase, defense/immunity protein |
| *LLPH* | *LLP homolog, long-term synaptic facilitation (Aplysia)* | 510518 | 5 | 47899635..47905608 | -- |
| *LOC101903752* | *uncharacterized LOC101903752* | 101903752 | 14 | 37551766..37562681 | -- |
| *LOC101905373* | *uncharacterized LOC101905373* | 101905373 | 14 | 55654025..55657919 | -- |
| *LOC101905560* | *uncharacterized LOC101905560* | 101905560 | 14 | 46529829..46533025 | -- |
| *LOC101905608* | *uncharacterized LOC101905608* | 101905608 | 14 | 46533126..46592661 | -- |
| *LOC101905780* | *uncharacterized LOC101905780* | 101905780 | 14 | 47437386..47779453 | -- |
| *LOC101906490* | *uncharacterized LOC101906490* | 101906490 | 18 | 19682875..19738674 | -- |
| *LOC101907320* | *uncharacterized LOC101907320* | 101907320 | 13 | 70465092..70491256 | -- |
| *LOC101907520* | *uncharacterized LOC101907520* | 101907520 | 5 | 54216851..54239268 | -- |
| *LOC104970694* | *heat shock transcription factor, Y-linked-like* | 104970694 | 9 | 93666765..93668120 | -- |
| *LOC780982* | *Acetyl-Coenzyme A acetyltransferase 2-like* | 780982 | 14 | 43781570..43783041 | -- |
| *LOC781434* | *histone H4* | 781434 | 14 | 50605995..50606386 | -- |
| *LOC782385* | *sentrin-specific protease 8* | 782385 | 14 | 42984671..42986018 | -- |
| *LOC782385* | *sentrin-specific protease 8* | 782385 | 14 | 42984671..42986018 | -- |
| *MED30* | *mediator complex subunit 30* | 613879 | 14 | 48940081..48959187 | -- |
| *MIR124A-2* | *microRNA mir-124a-2* | 100312992 | 14 | 30815411..30815521 | -- |
| *MRPS28* | *mitochondrial ribosomal protein S28* | 535290 | 14 | 45279167..45491347 | -- |
| *MSC* | *musculin* | 507407 | 14 | 37549339..37551351 | -- |
| *NOV* | *nephroblastoma overexpressed* | 505727 | 14 | 47005557..47013935 | growth factor |
| *NUP107* | *nucleoporin 107kDa* | 504823 | 5 | 45275633..45323840 | transporter |
| *PAG1* | *phosphoprotein membrane anchor with glycosphingolipid microdomains 1* | 614460 | 14 | 46335190..46362433 | -- |
| *PEX2* | *peroxisomal biogenesis factor 2* | 512677 | 14 | 42315032..42330414 | -- |
| *PKIA* | *protein kinase (cAMP-dependent, catalytic) inhibitor alpha* | 613524 | 14 | 43880469..43978275 | kinase inhibitor |
| *PMP2* | *peripheral myelin protein 2* | 506062 | 14 | 46801771..46806914 | transfer/carrier protein |
| *PPM1H* | *protein phosphatase, Mg2+/Mn2+ dependent, 1H* | 614880 | 5 | 50861212..51164478 | protein phosphatase, kinase inhibitor |
| *PUS7L* | *pseudouridylate synthase 7 homolog (S. cerevisiae)-like* | 505464 | 5 | 36905551..36927289 | -- |
| *RAP1B* | *RAP1B, member of RAS oncogene family* | 327708 | 5 | 45355213..45399173 | small GTPase |
| *RHNO1* | *RAD9-HUS1-RAD1 interacting nuclear orphan 1* | 513642 | 5 | 107374646..107384133 | -- |
| *RMDN1* | *regulator of microtubule dynamics 1* | 513788 | 14 | 78559789..78593879 | -- |
| *SAMD12* | *sterile alpha motif domain containing 12* | 783254 | 14 | 47779587..48312958 | -- |
| *SLC16A7* | *solute carrier family 16 (monocarboxylate transporter), member 7* | 614573 | 5 | 53987489..54214813 | transporter |
| *SLC35E3* | *solute carrier family 35, member E3* | 509009 | 5 | 45246215..45263395 | transporter |
| *SOX5* | *SRY (sex determining region Y)-box 5* | 533829 | 5 | 85867074..87037814 | HMG box transcription factor, nucleic acid binding |
| *SRGAP1* | *SLIT-ROBO Rho GTPase activating protein 1* | 539452 | 5 | 49807611..50119200 | G-protein modulator |
| *ST3GAL1* | *ST3 beta-galactoside alpha-2,3-sialyltransferase 1* | 282351 | 14 | 8898621..8989485 | glycosyltransferase |
| *STAU2* | *staufen double-stranded RNA binding protein 2* | 100125233 | 14 | 38983786..39192396 | DNA binding protein, RNA binding protein, deaminase, kinase activator, defense/immunity protein |
| *STMN2* | *stathmin 2* | 534991 | 14 | 44969002..45027084 | -- |
| *SYT1* | *synaptotagmin I* | 281511 | 5 | 8444602..9064806 | membrane trafficking regulatory protein |
| *TBC1D30* | *TBC1 domain family, member 30* | 541051 | 5 | 49178875..49272787 | hydrolase, G-protein modulator |
| *TMEM5* | *transmembrane protein 5* | 515147 | 5 | 50148338..50175016 | -- |
| *TNFRSF11B* | *tumor necrosis factor receptor superfamily, member 11b* | 523822 | 14 | 47432903..47461081 | tumor necrosis factor receptor |
| *TOP1* | *topoisomerase (DNA) I* | 534799 | 13 | 70383808..70481490 | DNA topoisomerase |
| *TPD52* | *tumor protein D52* | 540235 | 14 | 45498477..45550158 | -- |
| *TRHR* | *thyrotropin-releasing hormone receptor* | 281549 | 14 | 57476521..57541097 | G-protein coupled receptor |
| *TRNAC-ACA* | *transfer RNA cysteine (anticodon ACA)* | 100170901 | 14 | 37,347,803..37,347,874 | -- |
| *TRNAR-UCU* | *transfer RNA arginine (anticodon UCU)* | 100170908 | 13 | 69,462,660..69,462,732 | -- |
| *TRPA1* | *transient receptor potential cation channel, subfamily A, member 1* | 505317 | 14 | 37721460..37990162 | voltage-gated ion channel |
| *TRPS1* | *trichorhinophalangeal syndrome I* | 537652 | 14 | 50791816..51070252 | zinc finger transcription factor, nuclease |
| *WIF1* | *WNT inhibitory factor 1* | 533672 | 5 | 48917717..49009466 | -- |
| *ZFHX4* | *zinc finger homeobox 4* | 539762 | 14 | 41987711..42192539 | homeobox transcription factor, zinc finger transcription factor, structural protein, |
|  |  |  |  |  | RNA binding protein, actin family cytoskeletal protein |
| *ZHX2* | *zinc fingers and homeoboxes 2* | 515040 | 14 | 18368119..18552267 | homeobox transcription factor, zinc finger transcription factor, nucleic acid binding |
| *ZNF16* | *zinc finger protein 16* | 100294758 | 14 | 1443018..1459491 | KRAB box transcription factor |
| *ZNF704* | *zinc finger protein 704* | 513243 | 14 | 45982815..46225309 | transcription cofactor |
