# Supplementary material for: Candidate genes for male and female reproductive traits in Canchim beef cattle
Source: J Anim Sci Biotechnol. 2017 Aug 23;8:67. doi: 10.1186/s40104-017-0199-8 (PMC5569548; doi:10.1186/s40104-017-0199-8)
Supplement: Supplementary file 4 — Linkage disequilibrium for scrotal circumference at 420 d of age on chromosome 5. Fig. S2. Linkage disequilibrium for scrotal circumference at 420 d of age on chromosome 9. Fig. S3. Linkage disequilibrium for scrotal circumference at 420 d of age on chromosome 13. Fig. S4. Linkage disequilibrium for scrotal circumference at 420 d of age on chromosome 14. Fig. S5. Linkage disequilibrium for scrotal circumference at 420 d of age on chromosome 18. Fig. S6. Linkage disequilibrium for scrotal circumference at 420 d of age on chromosome 21. (ZIP 1985 kb) [file 40104_2017_199_MOESM4_ESM.zip › Additional file 4.docx]

Additional file 4 - Figure captions

Figure S1. Linkage disequilibrium for scrotal circumference at 420 days of age on chromosome 5.

Figure S2. Linkage disequilibrium for scrotal circumference at 420 days of age on chromosome 9.

Figure S3. Linkage disequilibrium for scrotal circumference at 420 days of age on chromosome 13.

Figure S4. Linkage disequilibrium for scrotal circumference at 420 days of age on chromosome 14.

Figure S5. Linkage disequilibrium for scrotal circumference at 420 days of age on chromosome 18.

Figure S6. Linkage disequilibrium for scrotal circumference at 420 days of age on chromosome 21.
