# Supplementary figures and images for: Candidate genes for male and female reproductive traits in Canchim beef cattle
Source: J Anim Sci Biotechnol. 2017 Aug 23;8:67. doi: 10.1186/s40104-017-0199-8 (PMC5569548; doi:10.1186/s40104-017-0199-8)

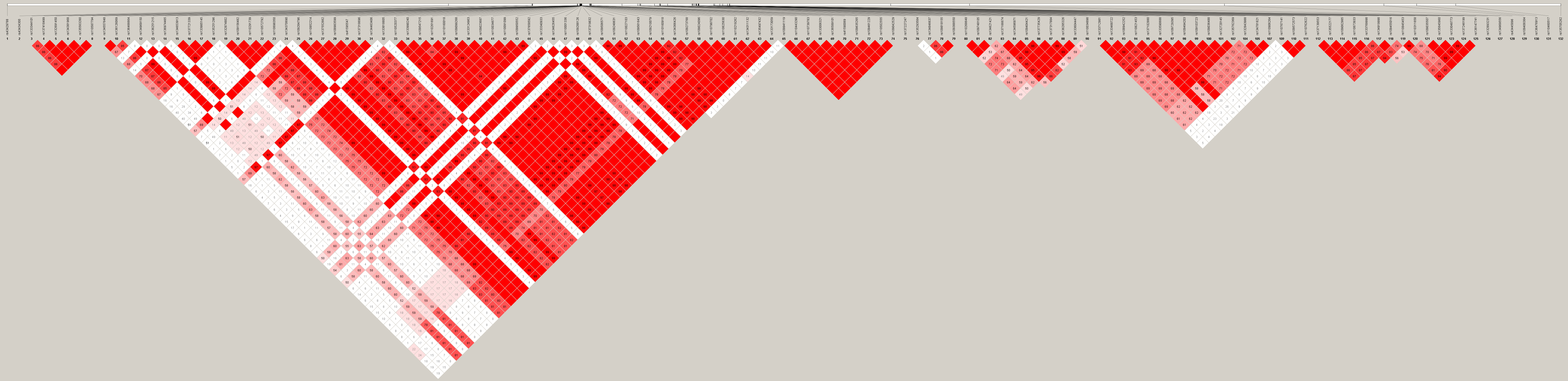

Supplement: Supplementary file 4 — Linkage disequilibrium for scrotal circumference at 420 d of age on chromosome 5. Fig. S2. Linkage disequilibrium for scrotal circumference at 420 d of age on chromosome 9. Fig. S3. Linkage disequilibrium for scrotal circumference at 420 d of age on chromosome 13. Fig. S4. Linkage disequilibrium for scrotal circumference at 420 d of age on chromosome 14. Fig. S5. Linkage disequilibrium for scrotal circumference at 420 d of age on chromosome 18. Fig. S6. Linkage disequilibrium for scrotal circumference at 420 d of age on chromosome 21. (ZIP 1985 kb) [file 40104_2017_199_MOESM4_ESM.zip › Figure S1.tiff]

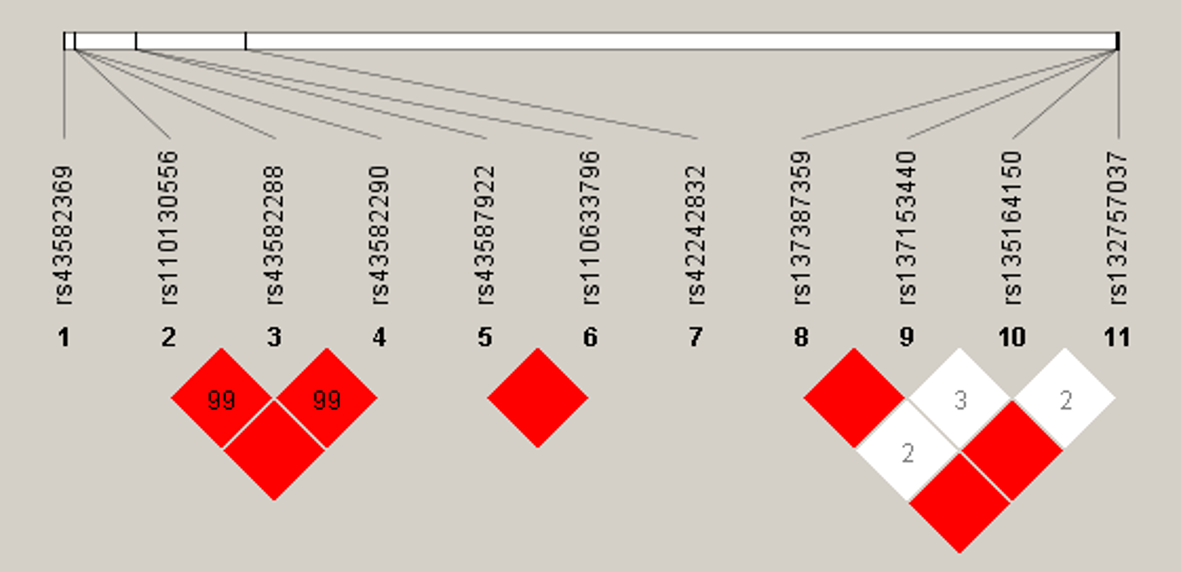

Supplement: Supplementary file 4 — Linkage disequilibrium for scrotal circumference at 420 d of age on chromosome 5. Fig. S2. Linkage disequilibrium for scrotal circumference at 420 d of age on chromosome 9. Fig. S3. Linkage disequilibrium for scrotal circumference at 420 d of age on chromosome 13. Fig. S4. Linkage disequilibrium for scrotal circumference at 420 d of age on chromosome 14. Fig. S5. Linkage disequilibrium for scrotal circumference at 420 d of age on chromosome 18. Fig. S6. Linkage disequilibrium for scrotal circumference at 420 d of age on chromosome 21. (ZIP 1985 kb) [file 40104_2017_199_MOESM4_ESM.zip › Figure S2.tiff]

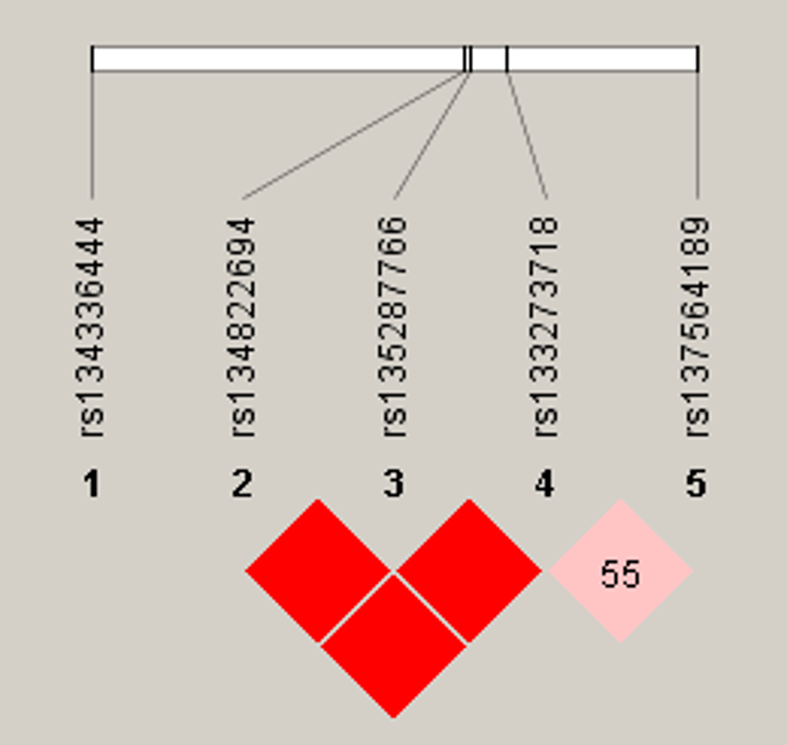

Supplement: Supplementary file 4 — Linkage disequilibrium for scrotal circumference at 420 d of age on chromosome 5. Fig. S2. Linkage disequilibrium for scrotal circumference at 420 d of age on chromosome 9. Fig. S3. Linkage disequilibrium for scrotal circumference at 420 d of age on chromosome 13. Fig. S4. Linkage disequilibrium for scrotal circumference at 420 d of age on chromosome 14. Fig. S5. Linkage disequilibrium for scrotal circumference at 420 d of age on chromosome 18. Fig. S6. Linkage disequilibrium for scrotal circumference at 420 d of age on chromosome 21. (ZIP 1985 kb) [file 40104_2017_199_MOESM4_ESM.zip › Figure S3.tiff]

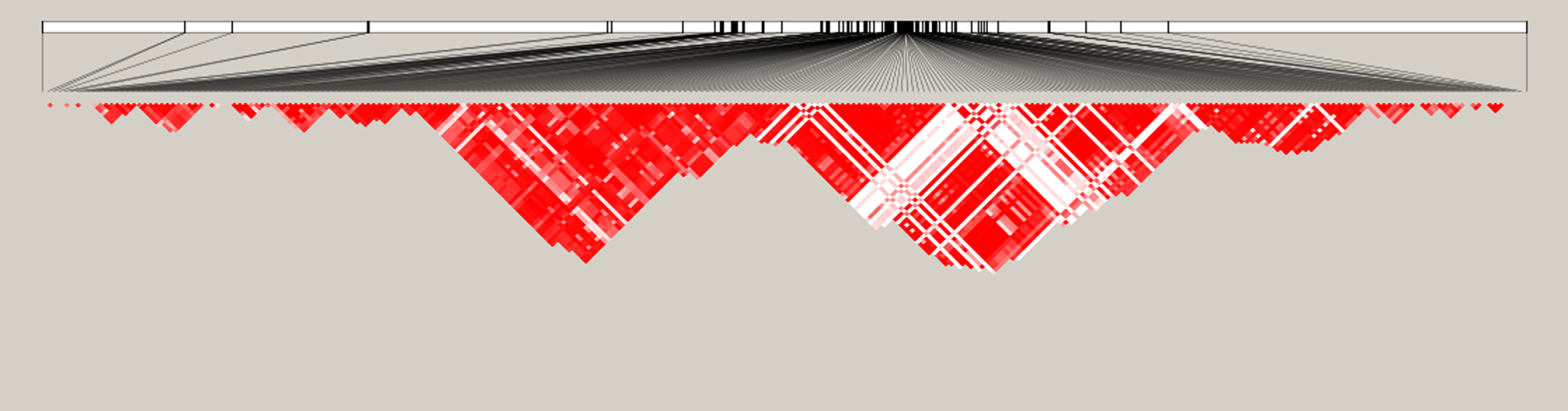

Supplement: Supplementary file 4 — Linkage disequilibrium for scrotal circumference at 420 d of age on chromosome 5. Fig. S2. Linkage disequilibrium for scrotal circumference at 420 d of age on chromosome 9. Fig. S3. Linkage disequilibrium for scrotal circumference at 420 d of age on chromosome 13. Fig. S4. Linkage disequilibrium for scrotal circumference at 420 d of age on chromosome 14. Fig. S5. Linkage disequilibrium for scrotal circumference at 420 d of age on chromosome 18. Fig. S6. Linkage disequilibrium for scrotal circumference at 420 d of age on chromosome 21. (ZIP 1985 kb) [file 40104_2017_199_MOESM4_ESM.zip › Figure S4.tiff]

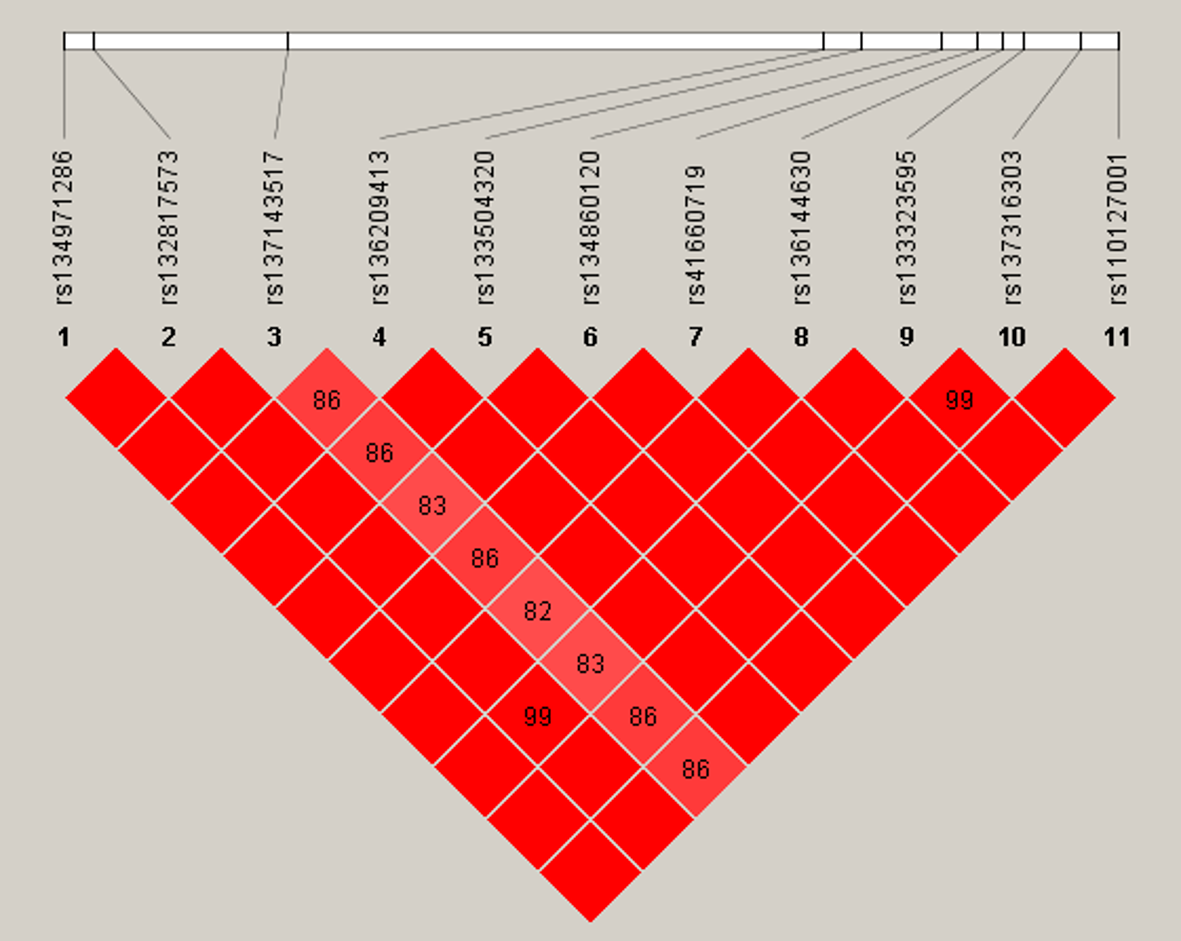

Supplement: Supplementary file 4 — Linkage disequilibrium for scrotal circumference at 420 d of age on chromosome 5. Fig. S2. Linkage disequilibrium for scrotal circumference at 420 d of age on chromosome 9. Fig. S3. Linkage disequilibrium for scrotal circumference at 420 d of age on chromosome 13. Fig. S4. Linkage disequilibrium for scrotal circumference at 420 d of age on chromosome 14. Fig. S5. Linkage disequilibrium for scrotal circumference at 420 d of age on chromosome 18. Fig. S6. Linkage disequilibrium for scrotal circumference at 420 d of age on chromosome 21. (ZIP 1985 kb) [file 40104_2017_199_MOESM4_ESM.zip › Figure S5.tiff]

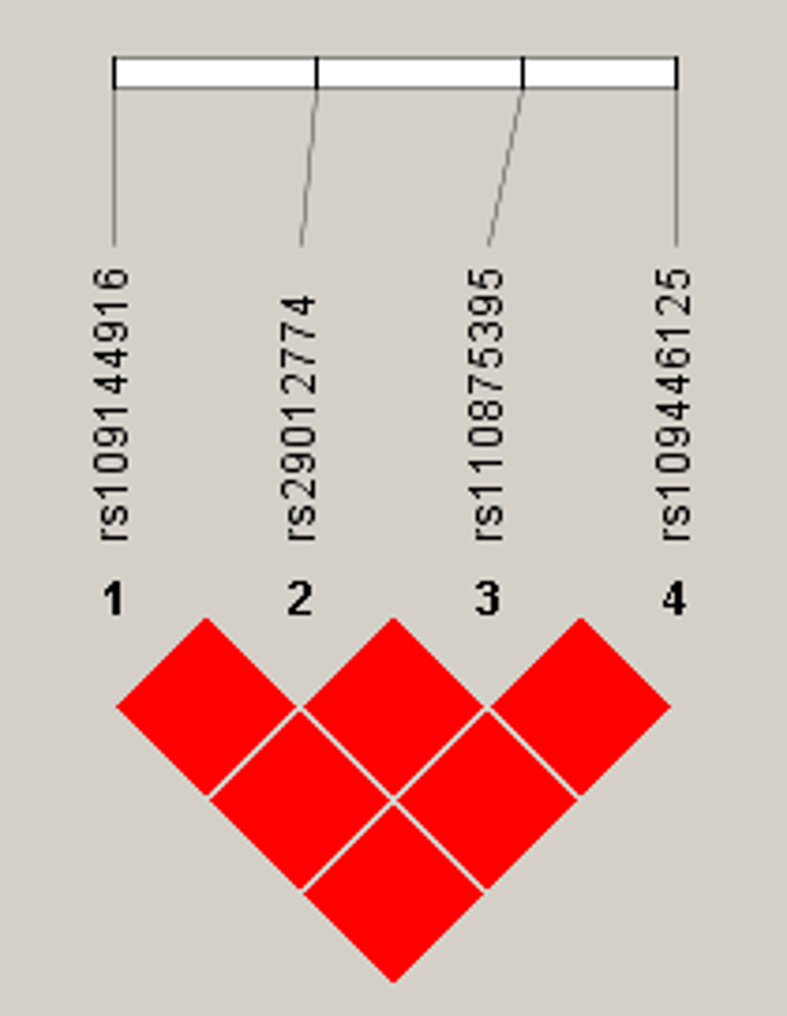

Supplement: Supplementary file 4 — Linkage disequilibrium for scrotal circumference at 420 d of age on chromosome 5. Fig. S2. Linkage disequilibrium for scrotal circumference at 420 d of age on chromosome 9. Fig. S3. Linkage disequilibrium for scrotal circumference at 420 d of age on chromosome 13. Fig. S4. Linkage disequilibrium for scrotal circumference at 420 d of age on chromosome 14. Fig. S5. Linkage disequilibrium for scrotal circumference at 420 d of age on chromosome 18. Fig. S6. Linkage disequilibrium for scrotal circumference at 420 d of age on chromosome 21. (ZIP 1985 kb) [file 40104_2017_199_MOESM4_ESM.zip › Figure S6.tiff]
